# Crop fields complement biodiversity in permanent grasslands across European landscapes

**DOI:** 10.1038/s41467-026-74356-7

**Published:** 2026-06-13

**Authors:** Fabian A. Boetzl, Giovanni Tamburini, Cristina Craioveanu, Andrei Crișan, Toshko Ljubomirov, Vlada Peneva, Laszlo Rakosy, Georg Rieland, Josef Settele, Anja Schmidt, Oliver Schweiger, Teodora Teofilova, Natalia Timuş, Martin Wiemers, Niklaus E. Zimmermann, Boyan Zlatkov, Ola Lundin, Erik Öckinger

**Affiliations:** 1 Swedish University of Agricultural Sciences, Department of Ecology, Uppsala, Sweden; 2 Department of Animal Ecology and Tropical Biology, Biocenter, University of Würzburg, Am Hubland, 97074 Würzburg, Germany; 3 Department of Soil, Plant and Food Sciences (DiSSPA – Entomology), University of Bari, Bari, Italy; 4 Babes-Bolyai University, Faculty of Biology and Geology, Department of Taxonomy and Ecology, Str. Clinicilor 5-7, 400006 Cluj-Napoca, Romania; 5 Romanian Lepidopterological Society, Str. Republicii 48, Cluj-Napoca, Romania; 6 Institute of Biodiversity and Ecosystem Research (IBER), Bulgarian Academy of Sciences (BAS), 1 Tsar Osvoboditel Blvd, 1000 Sofia, Bulgaria; 7 Institute of Biodiversity and Ecosystem Research (IBER), Bulgarian Academy of Sciences (BAS), 2 Gagarin Str., 1113 Sofia, Bulgaria; 8 Ștefan cel Mare University, Str. Universității 13, 720229 Suceava, Romania; 9 Helmholtz Centre for Environmental Research – UFZ, Department of Conservation Biology & Social-Ecological Systems, Halle & Leipzig, Germany; 10 Department for Nature Conservation and Landscape Planning, Anhalt University of Applied Sciences, Bernburg, Germany; 11 iDiv, German Centre for Integrative Biodiversity Research, Halle-Jena-Leipzig, Leipzig, Germany; 12 Helmholtz Centre for Environmental Research – UFZ, Department of Community Ecology, Halle (Saale), Germany; 13 Senckenberg - Leibniz Institution for Biodiversity and Earth System Research, Senckenberg German Entomological Institute, Müncheberg, Germany; 14 Swiss Federal Research Institute WSL, Birmensdorf, Switzerland; 15 Department of Environmental Systems Science, Eidgenössische Technische Hochschule (ETH) Zürich, Zürich, Switzerland

**Keywords:** Biodiversity, Conservation biology, Agroecology

## Abstract

Temperate agricultural landscapes are experiencing unprecedented biodiversity declines. Landscape simplification is commonly identified as a driver of species loss across taxonomic groups, but the contribution of crop and non-crop habitats to farmland biodiversity conservation is surprisingly poorly known. Using 86 paired permanent grasslands and oilseed rape fields in five European countries, we assess how habitat type shaped plant, butterfly, wild bee, and carabid assemblages and whether increasing grassland amount in surrounding landscapes fosters the spillover of grassland-associated biodiversity to oilseed rape fields. We find habitat type rather than landscape-level grassland amount determines diversity and shapes species assemblages: plants and butterflies are more diverse in grasslands, while wild bees and carabids are equally or more diverse in oilseed rape fields. Increasing landscape-level grassland amount affects species assemblage composition but only reduces turnover between habitats in wild bees. Overall, both grasslands and oilseed rape fields harbour distinct sets of species, together contributing complementarily to regional diversity. Safeguarding biodiversity in agricultural landscapes therefore requires not only the conservation of permanent semi-natural habitats but also biodiversity-friendly management of disturbed habitats such as crop fields that can contribute valuable species.

## Abstract (web summary)

Farmland biodiversity is in decline, but the role of different habitats in shaping biodiversity is unclear. This study finds that grasslands and oilseed rape fields support complementary species communities that together shape regional biodiversity.

## Introduction

Substantial changes in land-use and land management intensity over the last decades have triggered unprecedented declines in biodiversity^1,2^. These biodiversity declines jeopardise biodiversity-dependent ecosystem services such as pollination and pest regulation, and thus potentially compromise food security and agricultural sustainability^1,3^. In temperate agricultural landscapes, biodiversity declines have been linked to, among others, the loss of non-crop habitats and the homogenisation of agricultural landscapes^4^.

Non-crop habitats like permanent grasslands provide resources and shelter for many plant and animal species, are hence fundamental for conservation in agricultural landscapes and expected to provide crop fields with much of the biodiversity needed to ensure ecosystem functioning^5,6^. This expectation is known as the ‘landscape-moderated insurance hypothesis’^5^. It suggests that refuge habitat availability at the landscape-level can outweigh detrimental effects of local field management on biodiversity, thereby ensuring biodiversity-mediated ecosystem services, and has been supported by previous research^5,7^. Consequently, increasing non-crop habitat amount in agricultural landscapes is expected to increase spillover of individuals and foster biodiversity in crop fields nearby^8,9^, decreasing species turnover between non-crop habitats and crop fields.

Crop fields, however, also harbour considerable biodiversity, with many species reproducing and overwintering in them^10,11^. In agricultural landscapes, each habitat type provides unique conditions that accommodate the habitat preferences of a distinct assemblage of species, filtered from the regional species pool^7,12^. Many functionally important species, such as some bees and carabid beetles, can benefit from the resources and disturbance regimes characteristic of arable cropping^13,14^. Focussing on the conservation of species with low disturbance tolerance in permanent habitats is thus likely insufficient to halt biodiversity declines and ensure ecosystem service provision. Surprisingly, the contribution of different habitat types to the biodiversity in agricultural landscapes is little understood.

Here, we assess the contribution of semi-natural permanent grasslands and crop fields to landscape-wide species pools and investigate whether species assemblages in crop fields can be enriched by increasing landscape-wide non-crop cover across four taxonomic groups differing in foraging strategy and dispersal ability. Using a common sampling design, we compare the diversity of plants, butterflies, wild bees, and carabid beetles across 86 paired habitats in 43 landscapes in five European countries. In each landscape, a conventionally managed winter oilseed rape (*Brassica napus* L.) field and a semi-natural grassland, the predominant open non-crop habitat, were sampled repeatedly from the onset of oilseed rape flowering in late spring until after harvest. We hypothesise that: (i) due to higher continuity, more diverse resource availability and lower disturbance, species communities are more diverse in semi-natural grasslands than in oilseed rape fields, with oilseed rape fields harbouring a subset of the species assemblages in semi-natural grasslands, (ii) following the habitat amount hypothesis^15^, increasing landscape grassland amount increases biodiversity in grasslands and, via spillover, also in adjacent oilseed rape fields as predicted by the landscape-moderated insurance hypothesis^5^, and (iii) the spillover of grassland-associated species into oilseed rape fields reduces the turnover between semi-natural grasslands and oilseed rape fields as their species assemblages become more similar – a process modulated by taxon-specific movement patterns and habitat requirements.

## Results

Across all countries and habitats, we recorded a total of 837 species. We found 325 plant species and observed 8390 butterflies of 78 species, 2665 wild bees of 195 species, and 55737 carabid beetles of 239 species (Tables S10–S13). Species richness in oilseed rape fields and the paired grasslands was positively correlated in plants, butterflies and carabids (Fig. S3). In the following, we present results obtained from analyses of landscape characteristics at a 2000 m scale which had stronger support across models compared to a 1000 m scale (Tables S5–S8).

### Effects of habitat type on diversity and species assemblages

**Fig. 1:**
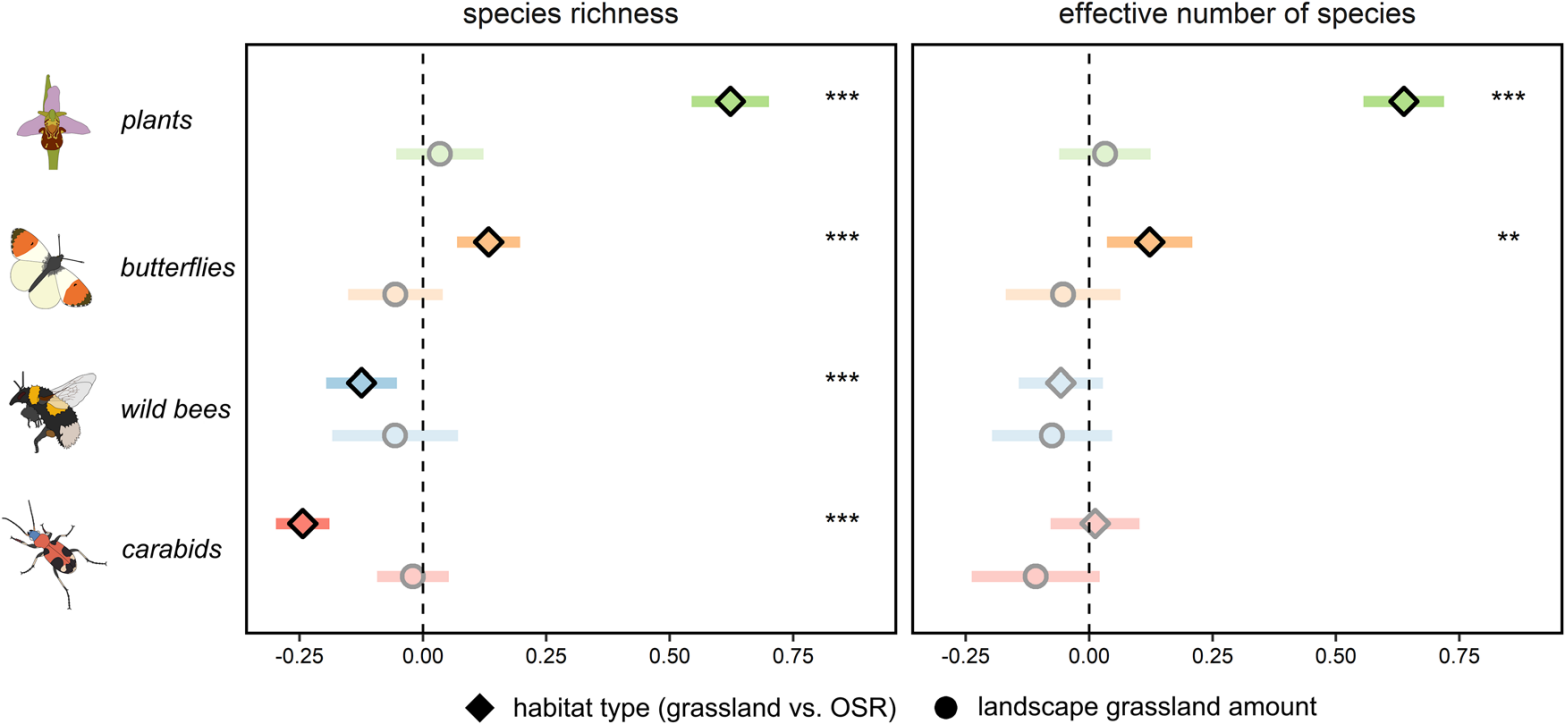
Effects of habitat type and landscape grassland amount on the diversity of plants, butterflies, wild bees, and carabids. Model coefficients for habitat type (diamonds; grassland vs. OSR; positive values indicate a higher value in grasslands) and landscape grassland amount (circles; positive values indicate a positive relationship) for the species richness and effective number of species in plants (*n* = 52; green), butterflies (*n* = 86; orange), wild bees (*n* = 86; blue), and carabids (*n* = 72; red) at the 2000 m scale (for results at the 1000 m scale, see Supplementary Note 1). Model coefficients on the log-scale taken from generalised linear mixed effects models, depicted with 95% confidence intervals. (*) indicates *p* < 0.1, * *p* < 0.05, ** *p* < 0.01, *** *p* < 0.001. For statistics, see text and Tables S5, S6 and S9. Plant and animal icons were created by F.A. Boetzl under a CC BY-SA 4.0 licence.

To better capture the effects of habitat type on local diversity, we complemented observed species richness, which is sensitive to rare species, with the effective number of species (the exponent of Shannon entropy), which accounts for species abundances and thus gives stronger weights to patterns driven by common species. The results only partially confirm our hypothesis that grasslands host more diverse communities: while both species richness and effective number of species of plants and butterflies were indeed higher in grasslands than in oilseed rape fields (Fig. 1; Tables S5 and S6), species richness of wild bees and carabids was higher in oilseed rape fields than in grasslands with no differences in the effective number of species of both taxa (Fig. 1; Tables S5 and S6). A complementary analysis standardising observed diversity by estimated sample completeness confirmed that grasslands were not consistently more diverse than oilseed rape fields across taxa. We found a higher effective number of species of plants and butterflies and a higher species richness of plants in grasslands, but no differences in species richness or effective number of species of wild bees or carabids (Supplementary Note 3). Differences in diversity between grasslands and oilseed rape fields varied across countries in all taxa (Figs. S8 and S11; Table S9).

**Fig. 2:**
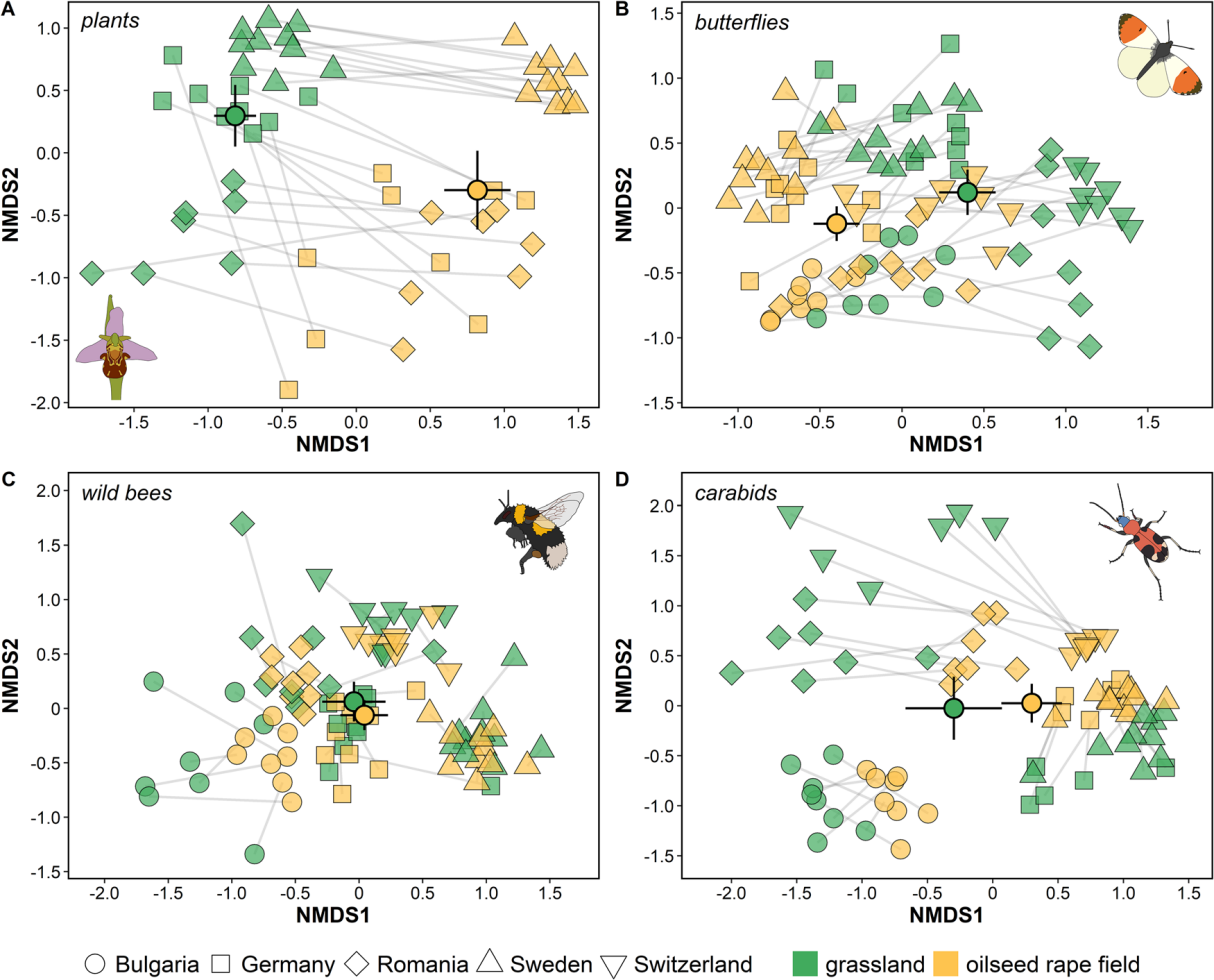
Species assemblages of four taxonomic groups (NMDS ordination) in the two sampled habitat types (oilseed rape and grassland) across five European countries (Bulgaria, Germany, Romania, Sweden and Switzerland). The taxonomic groups comprised plants (**A**; *n* = 52), butterflies (**B**; *n* = 86) wild bees (**C**; *n* = 86) and carabids (**D**, *n* = 72). Bold circles indicate centroids for the habitat types ± 95% confidence intervals. The NMDS used Bray-Curtis distances on proportional abundances or proportions of cover (plants), paired habitats are linked with grey lines. For statistics, see text and Table S7. For a display of species, see Fig. S19. Plant and animal icons were created by F.A. Boetzl under a CC BY-SA 4.0 licence.

Habitat type was, after country, the most influential predictor for the composition of species assemblages across taxa, with the strongest effect on plants and the weakest effect on wild bees (Fig. 2; Table S7). The distinctness of grasslands and oilseed rape field assemblages varied across countries in all taxa but wild bees, where patterns were consistent (Fig. 2; Table S7). Across all countries, we identified 145 of the 837 species as driving the distinctness of species assemblages between habitats with 82 species associated with grasslands (49 plant species, 21 butterfly species and twelve carabid species; 12.1% of the 676 species found in grasslands) and 63 species associated with oilseed rape fields (seven plant species, six butterfly species, ten wild bee species and 40 carabid species; 13.9% of the 454 species found in oilseed rape fields), though the sets of habitat-associated species differed considerably across the countries (Supplementary Note 2, Tables S10–S13).

Species assemblages of butterflies, wild bees, and carabids in oilseed rape fields were more similar across countries, whereas assemblages in permanent grasslands were more distinct likely because grasslands harbour more region-specific species shaped by local environmental conditions. (Fig. 2). Carabid assemblages in oilseed rape fields were especially similar across countries indicating a large assemblage overlap (Fig. 2), caused by these assemblages being dominated by common species associated with oilseed rape fields (Tables S13).

### Effects of landscape grassland amount on diversity

Contrary to our hypothesis, landscape-level grassland amount did not affect species richness or effective number of species of any of the taxa (Fig. 1; Tables S5 and S6). We, however, found landscape grassland amount affecting the composition of species assemblages in plants, butterflies and carabids with effects varying across habitat types for plants and across countries for plants and butterflies (Table S7).

### Effects of landscape grassland amount on beta diversity

**Fig. 3:**
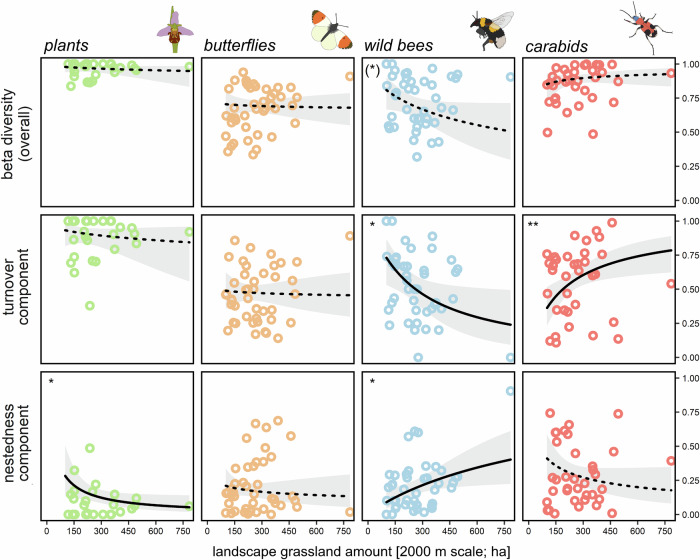
Beta diversity between paired habitats in relation to landscape grassland amount. Model predictions for the effects of landscape grassland amount at the 2000 m scale on overall beta diversity (Bray-Curtis dissimilarity) and its turnover and nestedness components between the paired habitats in plants (*n* = 26; green), butterflies (*n* = 43; orange), wild bees (*n* = 43; blue) and carabids (*n* = 36; red; estimated marginal means with 95% confidence interval; for results at the 1000 m scale, see Supplementary Note 1). Predictions from generalised linear mixed effects models are back-transformed from the log-scale. Solid lines indicate significant relationships (*p* < 0.05), dashed lines non-significant relationships (*p* > 0.05). (*) indicates *p* < 0.1, * *p* < 0.05, ** *p* < 0.01, *** *p* < 0.001. For statistics, see text and Tables S8 and S9. Plant and animal icons were created by F.A. Boetzl under a CC BY-SA 4.0 licence.

We expected decreasing turnover between the habitats with increasing landscape grassland amount. Overall, we found high beta diversity between the paired grasslands and oilseed rape fields across countries and taxa (0.79 ± 0.01), mainly driven by turnover rather than by nestedness (0.58 ± 0.02 vs. 0.21 ± 0.02; beta diversity ranges between 0 indicating full overlap and 1 indicating no overlap; Table S7, Fig. S12), showing that oilseed rape fields do not simply harbour subsets of grassland assemblages, but support distinct species assemblages. In contrast to our hypothesis, beta diversity was mostly unaffected by the amount of landscape grassland. The expected decrease of turnover between the paired habitats with increasing landscape grassland amount was only found for wild bees, while the opposite relationship was detected for carabids (Fig. 3 and Table S8). In addition, landscape grassland amount was negatively related to the nestedness component in plants and positively in wild bees (Fig. 3 and Table S8). Overall beta diversity as well as its turnover and nestedness components in all taxa and on both spatial scales also depended on country, either as a main effect or in interaction with landscape grassland amount (Tables S8 and S9, Figs. S14 and S15).

## Discussion

We show that both oilseed rape crop fields and non-crop habitats in the form of permanent grasslands contribute to biodiversity in agricultural landscapes, each hosting more or less distinct species assemblages. Contrary to our expectations, species richness and diversity was not consistently higher in permanent grasslands than in oilseed rape fields across all taxonomic groups. Moreover, an increasing amount of permanent grassland in the surrounding landscape did not alleviate dissimilarities in species composition between the paired habitats.

In contrast to our hypothesis, permanent grasslands were not generally more diverse than oilseed rape fields, with only plants and butterflies reaching a higher richness and effective number of species in grasslands, while wild bees and carabids had a higher richness in oilseed rape fields. A complementary, sample-completeness-based approach showed that both habitats were similarly important for the studied taxa, except for plant and butterfly richness and the effective number of species, which were consistently higher in grasslands (Supplementary Note 3). We found both habitats to be indeed highly complementary for local biodiversity across the four taxa and all sampling intervals (Figs. S17 and S18), with on average only 42 percent of the observed species shared between the grassland and oilseed rape field within a habitat type pair across all groups. Similar patterns of complementarity were previously reported between permanent grasslands and winter cereals for flowering plants^16^ and carabids^17^. This complementarity was driven by taxon- and species-specific landscape use, habitat requirements, and the species’ functional traits. Many species are in fact well adapted to the type of disturbance in crop fields that, in parts, resemble natural disturbance regimes, and therefore prefer crop fields^9^. Permanent non-crop habitats such as semi-natural grasslands, in contrast, also support species that do not tolerate frequent disturbances and are often more specialised, larger and weaker dispersers^18^. In our study, plant assemblages in grasslands were defined by grasses (29% of associated species) and legumes (14% of associated species) and other perennial plants, many of which were adapted to frequent grazing (e.g. *Bellis perennis, Plantago lanceolata*) and a low nutrient availability (e.g. *Anthoxanthum odoratum, Galium verum*), while plant assemblages in oilseed rape fields were defined by annual arable weeds (e.g. *Capsella bursa pastoris, Viola arvensis*) adapted to frequent soil disturbances and high nutrient availability^19^. It should be noted that such species might neither be desired from a farming perspective nor a main priority for conservation. However, they might provide structural heterogeneity and alternative food sources that benefit a higher diversity of the insect taxa. In contrast to the other groups, most butterflies and wild bees found in crop fields do not reproduce in these as they need specific food plants and nesting sites that are only found in permanent and undisturbed habitats like grasslands^10,20^. Mass-flowering crops such as oilseed rape are expected to attract pollinators from the surrounding landscape^21^ and hence harbour a subset of species that can forage on crop flowers. Oilseed rape fields were nevertheless found to be preferred by species that were only found in low numbers or absent in grasslands at the same point in time (Tables S11 and S12). In butterflies, 27% of the species mainly occurred on grasslands, but 9% of the recorded species were associated with oilseed rape fields. While grassland-associated species were often reproduction-limited and mostly reproduced on grasses or legumes, oilseed rape-associated species were multivoltine and reproduced on the crop itself, closely related brassicas or arable weeds not found in grasslands, highlighting the complementarity of the habitats (Fig. S13). In wild bees, none of the species was specifically associated with grasslands, likely due to their high mobility and the high turnover between grasslands within the same country (Fig. 2). Ten ground-nesting species were, however, associated with oilseed rape fields and likely benefitted from available bare soil nesting spots in the proximity of the fields or along tractor tracks (5% of all species recorded). Although these results indicate that oilseed rape fields can provide foraging and potentially nesting opportunities for a range of pollinator species, this effect is strongly dependent on the distance from the field edge^22^ and permanent and undisturbed habitats remain indispensable for sustaining their diversity in agricultural landscapes due to these permanent habitats meeting the requirements of specialist species and providing a continuous supply of food resources^23^. For carabids, only twelve predominantly granivorous species (5% of all species) were associated with grasslands, likely benefitting from the higher diversity of seed resources. In contrast, crop fields are expected to offer abundant food resources mainly for predatory species and to filter assemblages towards disturbance-tolerant species. In fact, the 40 species defining oilseed rape assemblages in our study (17% of all species) were mostly predatory and agrophilous species^24^ adapted to frequent disturbances that used to occur naturally in dynamic river systems and due to landslides in mountainous regions. Crop fields can act as alternative habitats for such species and accommodate their habitat requirements, and their suitability for their conservation can likely be increased by adopting biodiversity-friendly management. Our results demonstrate that both habitats filtered species assemblages based on the species’ ecological requirements, as shown in a more detailed analysis of the Romanian butterfly data^25^, and thus act complementarily. This complementarity was strongest for plants and carabids, intermediate for butterflies and least important for wild bees. The complementarity of species assemblages was also related to movement ability and expected landscape-use: the more mobile a taxon was, the weaker the effect of habitat type on its assemblages, and the fewer species were found associated with either habitat type. Consequently, we observed a few wild bees associated with one habitat type, and wild bee assemblages in both habitats were subsets of species identities and abundance distributions, with greater landscape-level grassland amount (i.e., increasing nestedness). The opposite relationship was observed for plants and carabids, in comparison less mobile taxa that are unable or less likely to use different habitats to complete their lifecycle.

The amount of permanent grassland in the surrounding landscape had, in contrast to our expectation, only limited effects on the indicators of biodiversity assessed here, but affected assemblage composition in all taxa. The complementarity of oilseed rape fields and permanent grasslands for biodiversity was thus largely independent of the amount of landscape grassland. This could be partly due to our study design, which always provided semi-natural grasslands in close proximity to the oilseed rape fields, which may already have enriched species assemblages in oilseed rape fields. Nevertheless, grasslands and oilseed rape fields harboured distinct assemblages even with a high grassland amount in the surrounding landscape. Our results indicate that landscape-wide gamma diversity is largely shaped by the high complementarity of the paired habitats for biodiversity, indicated by a high beta diversity between them. This finding backs the call for more diverse and heterogeneous landscapes in which different habitats can contribute to the overall species pool^26,27^.

The expected negative relationship between increasing grassland cover and species turnover was observed only in wild bees. Bees are central place foragers that predominantly nest in permanent, non-crop habitats and forage in the surrounding landscape, with foraging distances positively related to bee body-size^28^. With increasing grassland amount, distances between grassland nesting habitats and oilseed rape fields as foraging habitats decrease and more bee species spill over into oilseed rape fields^20^, leading to decreased turnover between the assemblages of both habitats types. In contrast, increasing grassland amount was positively related to the turnover of carabids between the paired habitats, many of which are weak dispersers and reproduce in crop fields or their margins^29^. Patterns of species turnover are thus likely determined by the species’ functional traits and their use of the landscape and cannot easily be generalised. The absence of a negative relationship between increasing grassland abundance and species turnover in taxa other than wild bees suggests that local habitat characteristics are shaping species assemblages and outweigh or negate potential effects of nearby non-crop habitats.

We found that patterns, especially the effects of landscape grassland amount, varied considerably across countries, likely due to differences in climate, land-use history, and management regimes for landscapes and habitats. This indicates that single case studies based on subsets of our data would report different, and depending on the geographic location, partly contrasting results than the joint analysis presented here. For the more mobile wild bees and butterflies, we may have underestimated the actual richness and diversity in landscapes with high permanent grassland amount, as large quantities of suitable habitat in a landscape can lead to a dilution of the landscape-wide species pool^5^. Analyses corrected for sample-completeness, however, yielded similar results for the effects of landscape grassland amount for these taxa (Supplementary Note 3). In addition, permanent grasslands are more variable than crop fields, with management differing both between and within habitat patches, resulting in a high degree of heterogeneity on both scales^30^ and varying habitat quality. Habitat amount may thus be less informative for explaining biodiversity patterns than habitat quality. A higher species turnover can be expected between different permanent grassland patches than between different oilseed rape fields. Management of both permanent grasslands and oilseed rape fields might modulate their complementarity for biodiversity by affecting their suitability for biodiversity. Unfortunately, we could not test this as detailed management information was unavailable (however, biodiversity complementarity was similar across a coarsely classified grassland management gradient, see Fig. S16). In addition, our results are, in part, also dependent on the choice of crop type. Oilseed rape is an attractive foraging resource for some bees during flowering^21^ and commonly reported to host rich carabid assemblages with high densities^24,31^, and thus likely not representative for all other crop types. We call for a wider quantification of the relative contribution of different crop types under different management regimes to biodiversity, as data is, for most crop types and especially crop-management combinations, largely missing.

In conclusion, our results indicate that in order to conserve a maximum of biodiversity in agricultural landscapes, it is essential but insufficient to conserve permanent grasslands as they alone can only support a subset of species, and not all species will disperse into adjacent crop fields where ecosystem services such as pollination or pest regulation are needed. Local filters on species assemblages, i.e., properties and management of habitats, might outweigh potential beneficial effects of landscape composition. Non-crop habitats thus need to be complemented with biodiversity-friendly crop management to support species in and adapted to crop fields. This combined approach will be required to create sustainable, future-proof agricultural landscapes that support both crop production and biodiversity in agricultural landscapes.

## Methods

### Study design

**Fig. 4:**
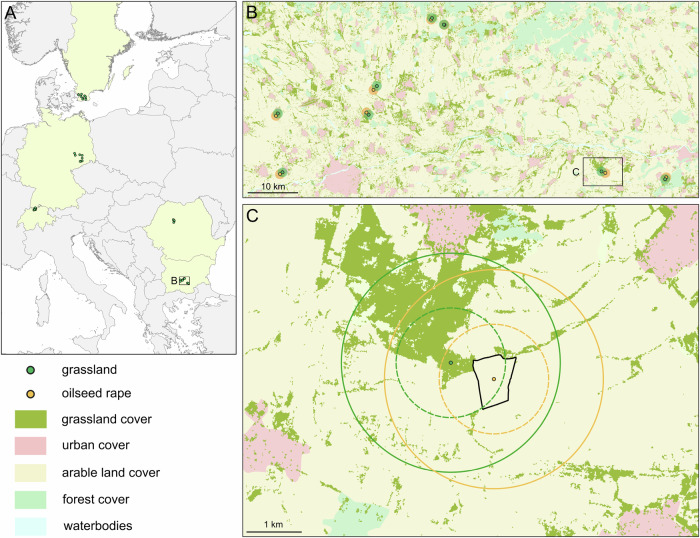
Overview of the study design at European, regional, and landscape scales. Location of the 43 studied landscapes (green dots) in the five European countries (yellow), Sweden, Germany, Switzerland, Romania and Bulgaria (**A**). As an example, we show the Bulgarian study region (**B**) with semi-natural grasslands indicated by green dots, and oilseed rape fields by orange dots (both with 1000 m buffer). For a display of all study sites in all study regions, see Fig. S1. Zooming in on one of the habitat pairs (**C**), we show the extent of the 1000 m (dashed line) and 2000 m (solid line) buffer radii with the oilseed rape field indicated by a black outline. Basic maps were generated based on the 2018 CORINE land cover raster^46^ with an overlay of the high-resolution permanent grassland raster^33^ used for the analyses (olive green).

We selected 86 paired conventional winter oilseed rape fields and permanent, semi-natural grasslands in 43 landscapes across five European countries: Bulgaria (*n* = 8), Germany (*n *= 9), Romania (*n* = 8), Sweden (*n *= 10), and Switzerland (*n* = 8; Fig. 4 and Fig. S1). All habitats were embedded within traditional agricultural landscapes at elevations from 15 m to 515 m: In Bulgaria, the landscapes were dispersed across the agricultural area of the Thracian lowland, with some reaching the foothills of the Sarnena Sredna Gora mountain range, at 115 m to 310 m elevation (humid subtropical climate Cfa, annual precipitation <600 mm). Landscapes in Germany were located in the intensive agricultural region of northern Saxony around Leipzig between 95 m and 380 m elevation (temperate oceanic climate Cfb, annual precipitation ~ 720 mm). The Romanian landscapes were located in agriculturally dominated areas of the Transylvanian Plateau around Cluj at 325 m–515 m elevation (warm-summer humid continental climate Dfb, annual precipitation ~ 840 mm). In Sweden, landscapes were dispersed throughout the intensive agricultural region of Skåne in the south of the country at 15 m–170 m elevation (temperate oceanic climate Cfb, annual precipitation ~ 760 mm). Swiss landscapes were located in the Swiss Jura northwest of Zürich at 350 m–490 m elevation (temperate oceanic climate Cfb, annual precipitation ~ 1500 mm; all climate zone information based on the Köppen climate classification^32^.

Habitat pairs were selected along a gradient of permanent grassland cover in the surrounding landscapes. The average distance between the paired habitats was 149.8 ± 57.2 m (range: 0.5 to 594.8 m; edge to edge; Fig. 4). Selected oilseed rape fields ranged in size from 0.9 ha to 92.1 ha (average ± SE: 15.7 ± 3.0 ha) with generally smaller fields in Switzerland and Romania (Table S1). All selected grassland patches had existed for at least 30 years and were neither fertilised, tilled, nor improved by sowing. The size of the selected grasslands could not always be clearly delimited as some of the grasslands in all countries, but especially in Bulgaria and Romania, formed part of a continuous grassland mosaic spanning the landscapes. In addition, grasslands varied between and within countries depending on soil type, climate, and management type and intensity. Most grasslands were managed by grazing (30 patches), the majority being grazed extensively (20 patches; <1 livestock units) while some grasslands were grazed at higher intensities (10 patches; (<5 livestock units). A further 16 grasslands were mown, predominantly once per year at the end of the growing season in July or early August (Table S2). Four grasslands were mown and/or grazed depending on the respective year, with two being mown in late July and grazed by shepherds thereafter in autumn, and the remaining two being either mown once or twice per year or grazed at low intensity (<1 livestock units). One grassland was completely unmanaged (Table S2).

### Assessing grassland cover

As a potential biodiversity-rich source habitat for the species communities inhabiting agricultural landscapes, we quantified the cover of permanent grasslands in the studied landscapes. To ensure a consistent classification of permanent grasslands across all countries, we used the pan-European high-resolution grassland raster provided by the Copernicus Land Monitoring Service of the European Union for the European Environmental Agency^33^. This raster contains all permanent natural, semi-natural, and managed grasslands without soil disturbance across Europe with a pixel size of 10 × 10 m (Fig. 4). For our analyses, we used the 2018 grassland raster, as it was the closest in time to the time points of data collection and permanent grassland cover should not vary significantly within one year due to the permanent nature of the habitats (overall accuracy: 90.0%; precision: 83.3%; recall: 96.1%^33^). We extracted the amount of permanent grassland within circular buffers around the outline centroid of all oilseed rape fields and grassland habitat patches at two different spatial scales, 1000 m and 2000 m. The final grassland amount obtained included all permanent grasslands irrespective of habitat quality within the given radius, i.e. also the selected high-quality grassland patches. Permanent grasslands covered 21.3 ± 1.2% (range: 4.9 to 59.7%) at the 1000 m scale and 21.6 ± 1.1% (range: 7.6 to 66.1%) at the 2000 m scale across all landscapes but varied across countries (Fig. S2 and Table S3).

### Data collection & processing

We sampled four taxonomic groups, plants, butterflies, wild bees and carabids using established methods within 10 m from the habitat edge in both habitats in a standardised sampling design across countries in 2017 (Germany, Romania, Sweden, and Switzerland) and 2018 (Bulgaria). Access to the study sites was granted by the landowners and samples were gathered and processed by local experts in the participating countries. No further permissions were needed for data collection in Bulgaria, Germany, Romania, Sweden, and Switzerland.

Plant diversity was assessed at two time points, during oilseed rape flowering and fruit development (crop stages BBCH 60 and 74^34^) and post-harvest. In each habitat, five 1 m × 1 m plots spaced 10 m apart were surveyed. Plots were located along a transect parallel to the habitat border at 5 m to the edge in grasslands and at 10 m to the edge in oilseed rape fields. No plant data were available from Bulgaria and Switzerland. Plants were identified in the field using regional identification keys and species-specific vegetation cover was recorded in six classes (<1%, <5%, <12.5%, <25%, <50%, and >50%). Cover classes were transformed to their midpoint values, and the resulting percentages were used as estimates of plant abundance in the analyses. The sown crop species, oilseed rape (*Brassica napus* L.) was excluded from all analyses.

Butterflies were recorded four times along a 500 m transect along the habitat edge in oilseed rape fields and parallel to the edge at 5 m in grasslands. Each transect was subdivided into 50 m segments to capture small-scale variability in topography and vegetation types, and surveys followed the standard protocols of European butterfly monitoring schemes^35^. Transects were walked at a constant slow pace, and all butterflies within 5 m above ground, 5 m in front of the observer and 2.5 m to either side of the observer were recorded. All butterflies were identified in the field. Whenever necessary for safe identification, butterflies were briefly captured with a sweep net for identification and subsequently released. Surveys were conducted immediately before or during oilseed rape flowering (BBCH 59-67), twice during fruit development and ripening (BBCH 65-98), and post-harvest (or during the latest ripening stages in Sweden), at approximately monthly intervals. In Bulgaria, only two surveys were conducted, during peak flowering (BBCH 63-65) and late ripening (BBCH 86-88). Surveys were carried out between 9.00 and 17.00 on warm (>17 °C), sunny days (>30% sun) with no strong winds and no rainfall within the preceding hour.

Wild bees were recorded using a combination of pan traps (two sampling rounds) and standardised transect walks (four rounds). Along one edge of each habitat, five sets of coloured pan traps (red, blue and yellow; 15 cm diameter, 2 cm depth; 400 ml water + some drops of dishwashing detergent) were placed at vegetation height for 24 hours with a minimum spacing of 15 m between trap sets. The first sampling round coincided with peak oilseed rape flowering (BBCH 63-67), and the second with fruit development and ripening (BBCH 68-88). In Germany and Romania, only a single pan-trap round was conducted during peak flowering to early ripening (BBCH 65-70 and 65-79, respectively). Transect walks were conducted along 100 m transects parallel to the field edge at 5 m distance in oilseed rape fields and extending from the habitat edge towards the centre in grasslands. During surveys, insects were collected with constant sweeps with a net (mesh size ≤ 100 µm) alternating to the left and right of the transect up to 1 m on either side and from the ground level to vegetation height. Surveys were conducted immediately before or during oilseed rape flowering (BBCH 59-67), twice during fruit development and ripening (BBCH 65-80 and 79-98), and post-harvest (or during the latest ripening stages in Bulgaria and Sweden), at approximately monthly intervals. In Bulgaria and Switzerland, only two surveys were conducted, during peak flowering (BBCH 65-67) and during ripening (BBCH 75-88). Collected specimens were preserved in 70% ethanol, and all wild bees were subsequently identified in the lab. Specimens from Bulgaria, Germany, Romania, and Sweden (partly) are deposited at the Institute of Biodiversity and Ecosystem Research (IBER; Sofia/Bulgaria), specimens from Switzerland at the University of Neuchâtel (UniNE; Neuchâtel/Switzerland) and remaining specimens from Sweden at the Swedish University of Agricultural Sciences (SLU; Uppsala/Sweden). For analysis, data from pan traps (77.5% of individuals) and transect walks (22.5%) were pooled at the habitat level. The domesticated honeybee, *Apis mellifera*, was excluded from all analyses.

Carabids were sampled using pitfall traps in three sampling rounds. Five pitfall traps (12 cm height, 9 cm diameter; saturated 6% salt-acetic acid solution + some drops of dishwashing detergent) were installed along a transect extending from the habitat edge towards the centre, starting at 10 m from the edge and spaced 25 m apart in both habitats. In some grazed grasslands, traps were instead installed parallel to the habitat edge at 10 m distance and protected with fencing to prevent disturbance by livestock. Sampling was conducted immediately before or during oilseed rape flowering (BBCH 58-70), during fruit development and ripening (BBCH 68-98), and post-harvest. In each round, traps were active for 20 days, although this period varied slightly due to unforeseen complications, resulting in an overall average of 21.8 ± 0.1 days (average ± SE). However, identical exposure duration was maintained for all traps in the paired habitats. Trap loss occurred due to management activities or wildlife disturbance (188 traps or 13.5%). To account for unequal sampling effort that affects abundances and accumulated richness of carabids, we standardised sampling effort within each habitat pair. We identified traps that were lost in each habitat and removed the corresponding trap samples from the same transect position and sampling interval in the paired habitat, thereby excluding an additional 125 trap samples (9.0%). We further excluded habitat pairs with fewer than ten of the 15 traps remaining to ensure that assemblages were sampled sufficiently (six pairs). This resulted in 13.2 ± 0.3 pitfall trap samples per habitat in each pair (range: 10 to 15; median: 14). Carabids were identified in the lab and specimens from Bulgaria, Germany, Romania and Switzerland are stored at the Institute of Biodiversity and Ecosystem Research (IBER; Sofia/Bulgaria), specimens from Sweden at the Swedish University of Agricultural Sciences (SLU; Uppsala/Sweden).

### Statistical analyses

Before analyses, all data were pooled at the habitat level across all sampling intervals and transect positions by summing the number of individuals, except for plants, where we calculated the average cover across all sampling intervals and transect positions in each habitat.

All statistical analyses were performed in R 4.4.1 for Windows^36^. We used generalised linear mixed effects models (GLMM, package ‘glmmTMB’ version 1.1.7^37^) to test for the effects of habitat type and permanent grassland amount in the surrounding landscapes on the four taxa. We fitted separate models for the responses ‘species richness’ and ‘effective number of species’ (including the distribution of the species’ abundances within the communities^38^) for each taxon and spatial scale, 1000 m and 2000 m. Each model contained the fixed effects ‘habitat type’ (factor, 2 levels), ‘grassland amount’ (continuous), and ‘country’ (factor, 4 or 5 levels) as well as all two-way interactions between these to account for potential varying effects of grassland amount and habitat identity depending on country. In all models, ‘grassland amount’ was log-transformed following Watling, et al.^39^. All models containing both habitats, included the habitat pair (i.e. landscape ID) nested within country as a random intercept. All models used negative binomial residual distributions and models for carabids additionally included an offset for ‘sampling intervals’, i.e., a measure of sampling effort per habitat pair (log-transformed), to account for variation in sampling effort between habitat pairs.

In addition, we assessed the effects of habitat type and grassland amount on the assemblage composition of the four taxa using nonmetric multidimensional scaling (NMDS, ‘metaMDS’, 999 permutations, Bray-Curtis distances). Prior to ordinations and subsequent analyses, species matrices were standardised using the proportions of the species or, in the case of plants, the proportional area covered. We tested for statistically significant differences between assemblages using a permutational multivariate analysis of variance (PERMANOVA; ‘adonis2’ from the ‘vegan’ package, version 2.6–4^40^; 9999 permutations, Bray-Curtis distances) including ‘habitat type’ (factor, 2 levels), ‘grassland amount’ (continuous) and ‘country’ (factor, 4 or 5 levels) as well as all two-way interactions between these to account for potential varying effects of grassland amount and habitat identity depending on country. The PERMANOVA tests additionally included the distance between the paired habitats as well as the pair identity as a strata object. Separate PERMANOVA tests were calculated for the two spatial scales: 1000  m and 2000 m. Finally, to identify species driving assemblage differences between the habitat types, we used paired Wilcoxon tests on species incidences (plants) and paired t-tests on abundances (other taxa; transformed prior to analyses with log(x + 1) with x being the abundance to improve normality). Separate tests were performed between paired habitats for each country as species pools and likely also species’ habitat preferences vary considerably across Europe. In line with our abundance-based assemblage analyses, this approach identifies common and assemblage-defining species rather than rare species.

To test the effects of landscape grassland amount on beta diversity, we first calculated overall Bray-Curtis dissimilarity as well as its turnover and nestedness components between each habitat pair using the ‘beta.pair.abund’ function from the betapart package (version 1.6^41^). We then used GLMMs (‘glmmTMB’), testing the responses against the fixed effects ‘country’ (factor, 4 or 5 levels), ‘grassland amount’ (continuous), and their interaction to allow slopes to vary across countries. The models additionally included the distance between the paired habitats (continuous) and used a beta distribution. In wild bees, two sites had an overall dissimilarity as well as a turnover component of 1 which is outside the boundaries of beta regressions, and these values were thus replaced by ‘0.9999’. If data contained zeros, we used hurdle models (Table S8). As these models used a value calculated between paired habitats as response, we used the average grassland amount around both paired habitats that was strongly positively correlated with the grassland amounts around both habitat types (Pearson: r > 0.95; p < 0.001).

In all models, continuous predictors were z-scaled to multiples of the standard deviation and mean-centered using the ‘scale’ command. All models were checked for under- and overdispersion, zero inflation, and suitability of chosen residual distributions using the DHARMa package (version 0.4.6^42^) and we detected no violation of the model assumptions. Model outputs were obtained using type III sums of squares Wald chi-square tests with the command ‘Anova’ (library ‘car’, version 3.1-2^43^) and R^2^ values with the command ‘performance’ (library ‘performance’, version 0.10.4^44^).

### Reporting summary

Further information on research design is available in the Nature Portfolio Reporting Summary linked to this article.

## Supplementary information

Supplementary Information

Reporting Summary

Transparent Peer Review file

## Data Availability

The work is based on data collected within the BiodivERsA/FACCE-JPI funded project “SusTaining AgriCultural ChAnge Through ecological engineering and Optimal use of natural resources (STACCATO)” (www.staccato-project.net). The raw species diversity data and accompanying metadata files used in this study are available from Zenodo at 10.5281/zenodo.14044711^45^.

## Code availability

All analyses were performed in R 4.4.1^36^. The fully reproducible workflow, including an R markdown file with the code and all data files, is available from Zenodo at 10.5281/zenodo.14044711^45^.
