# Supplementary material for: Crop fields complement biodiversity in permanent grasslands across European landscapes
Source: Nat Commun. 2026 Jun 13;17:5263. doi: 10.1038/s41467-026-74356-7 (PMC13264617; doi:10.1038/s41467-026-74356-7)
Supplement: Supplementary file 1 — Supplementary Information [file 41467_2026_74356_MOESM1_ESM.pdf]

Supplementary Information for:

Crop fields complement biodiversity in permanent grasslands across European landscapes

Fabian A. Boetzl[1,2*], Giovanni Tamburini[3], Cristina Craioveanu[4], Andrei Crișan[5], Toshko Ljubomirov[6], Vlada Peneva[7], Laszlo Rakosy[4,5,8], Georg Rieland[9,10], Josef Settele[9,11], Anja Schmidt[9], Oliver Schweiger[11,12], Teodora Teofilova[6], Natalia Timuş[4], Martin Wiemers[9,12,13], Niklaus E. Zimmermann[14,15], Boyan Zlatkov[6], Ola Lundin[1] & Erik Öckinger[1]

[1] Swedish University of Agricultural Sciences, Department of Ecology, Uppsala, Sweden

[2] Department of Animal Ecology and Tropical Biology, Biocenter, University of Würzburg, Am Hubland, 97074 Würzburg, Germany

[3] Department of Soil, Plant and Food Sciences (DiSSPA – Entomology), University of Bari, Bari, Italy

[4] Babes-Bolyai University, Faculty of Biology and Geology, Department of Taxonomy and Ecology, Str. Clinicilor 5-7, 400006 Cluj-Napoca, Romania

[5] Romanian Lepidopterological Society, Str. Republicii 48, Cluj-Napoca, Romania

[6] Institute of Biodiversity and Ecosystem Research (IBER), Bulgarian Academy of Sciences (BAS), 1 Tsar Osvoboditel Blvd., 1000 Sofia, Bulgaria

[7] Institute of Biodiversity and Ecosystem Research (IBER), Bulgarian Academy of Sciences (BAS), 2 Gagarin Str., 1113 Sofia, Bulgaria

[8] Ștefan cel Mare University, Str. Universității 13, 720229 Suceava, Romania

[9] Helmholtz Centre for Environmental Research – UFZ, Department of Conservation Biology & Social-Ecological Systems, Halle & Leipzig, Germany

[10] Department for Nature Conservation and Landscape Planning, Anhalt University of Applied Sciences, Bernburg, Germany

[11] iDiv, German Centre for Integrative Biodiversity Research, Halle-Jena-Leipzig, Leipzig, Germany

[12] Helmholtz Centre for Environmental Research – UFZ, Department of Community Ecology, Halle (Saale), Germany

[13] Senckenberg - Leibniz Institution for Biodiversity and Earth System Research, Senckenberg German Entomological Institute, Müncheberg, Germany
[14] Swiss Federal Research Institute WSL, Birmensdorf, Switzerland
[15] Department of Environmental Systems Science, Eidgenössische Technische Hochschule (ETH) Zürich, Zürich, Switzerland
*Corresponding author: fabian.botzl@slu.se

Table of contents

Figure S1. Location of the 43 studied landscapes

Figure S2. Permanent grassland cover within the two buffer radii

Table S1. Sizes of oilseed rape fields across the five countries

Table S2. Grassland management information

Table S3. Permanent grassland cover across the five countries and two buffer radii

Supplementary Note 1: Results on the 1000 m spatial scale

Figure S3. Relation between species richness in the paired habitats

Figure S4. Model coefficients for habitat type and landscape grassland amount for species richness and effective number of species

Figure S5. Activity densities across habitat types and countries

Figure S6. Species richness in relation to grassland amount and habitat type

Figure S7. Species richness in relation to grassland amount and country

Figure S8. Species richness across habitat types and countries

Figure S9. Effective number of species in relation to grassland amount and habitat type

Figure S10. Effective number of species in relation to grassland amount and country

Figure S11. Effective number of species across habitat types and countries

Figure S12. Overall Bray-Curtis beta-diversity between paired grasslands and oilseed rape fields and its turnover and nestedness components

Figure S13. Model coefficients for the effects of grassland amount on overall beta-diversity and its turnover and nestedness components

Figure S14. Turnover component in relation to grassland amount and country

Figure S15. Nestedness component in relation to grassland amount and country

Figure S16. Diversity across taxa in response to grassland management.

Table S4. Species richness of plants, butterflies, wild bees and carabids for each habitat and country

Table S5. Model results for species richness

Table S6. Model results for the effective number of species

Table S7. PERMANOVA results

Table S8. Model results for beta diversity and its turnover and nestedness components

Table S9. Estimated marginal means for all responses and countries

Supplementary Note 2: Species driving habitat differences across countries
Figure S17. Species richness difference between paired habitats across oilseed rape phenology
Figure S18. Species shared between paired habitats across oilseed rape phenology
Figure S19. Ordinations with country-exclusive and habitat-associated species.
Figure S20. Distribution of selected traits in habitat-associated species.
Table S10. Plant species recorded habitat types and countries
Table S11. Butterfly species recorded habitat types and countries
Table S12. Wild bee species recorded habitat types and countries
Table S13. Carabid species recorded habitat types and countries
Supplementary Note 3: Alternative analyses for species richness and the effective number of species
Figure S21. Observed sample completeness across habitats and taxa
Figure S22. Model coefficients for habitat type and landscape grassland amount for species richness and effective number of species at standardised sample completeness.
Figure S23. Observed sample completeness across habitats and taxa at standardised abundance
Figure S24. Model coefficients for habitat type and landscape grassland amount for species richness and effective number of species at standardised abundance.
Table S14. Estimated sample completeness across countries and habitats
Table S15. Model results for species richness at standardised sample completeness
Table S16. Model results for the effective number of species at standardised sample completeness
Table S17. Observed share of singleton species across countries and habitats
Table S18. Model results for species richness at standardised abundance
Table S19. Model results for the effective number of species at standardised abundance

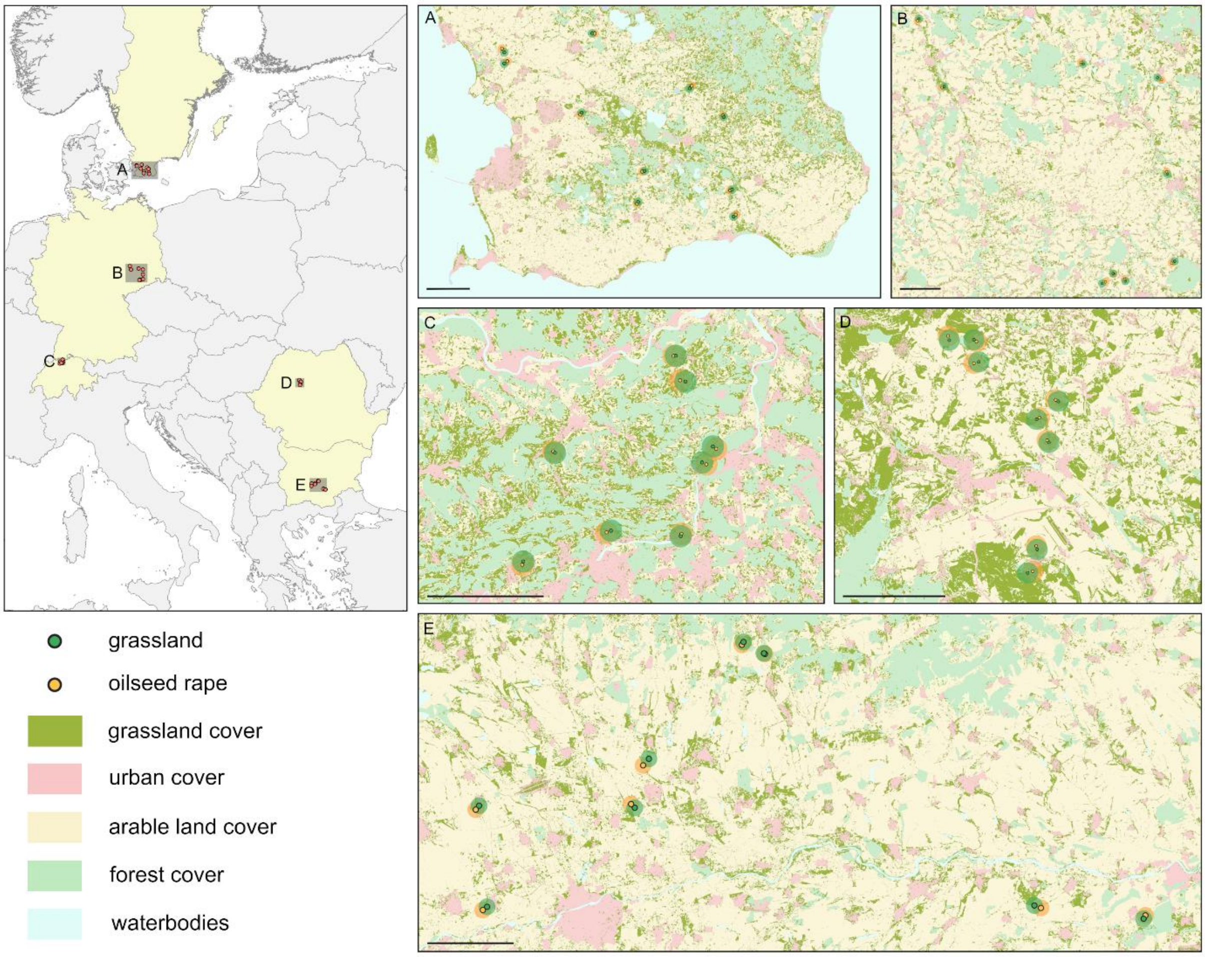

Fig. S1: Location of the 43 studied landscapes (red circles) in the study regions (grey areas) in the five European countries (yellow), Sweden (A), Germany (B), Switzerland (C), Romania (D) and Bulgaria (E). Semi-natural grassland sites are indicated by green circles, oilseed rape fields by orange circles (both with 1000 m buffer radius). Black lines in the lower left corners represent 10 km distance. Basic maps were generated based on the 2018 CORINE land cover maps with an overlay of the high-resolution grassland raster used for the analyses (light green).

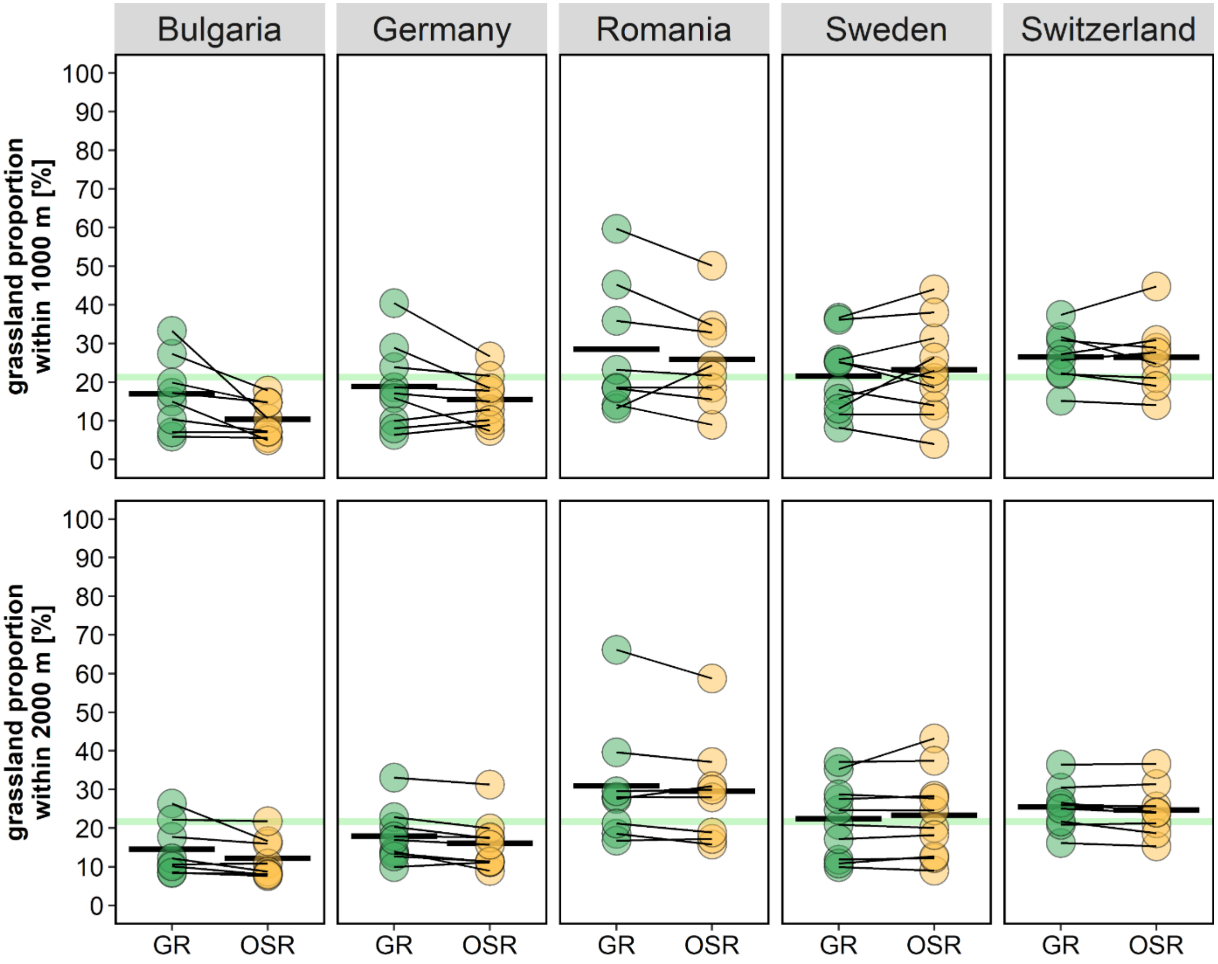

Fig. S2: Permanent grassland cover (%) within the 1000 m and 2000 m radii across all selected oilseed rape fields (OSR, orange) and permanent grasslands (GR, dark green) across the five countries. Paired oilseed rape fields and grassland plots are connected, the solid black lines represent the means in each country and habitat type, the solid light green lines represent the overall mean across countries and habitat types.

Table S1: Number and average size as well as size range of the winter oilseed rape fields in the five countries and overall. Field sizes in hectare [ha], rounded to one decimal.

country	number of fields	average ± standard error	range [min; max]
Bulgaria	8	26.4 ± 10.8	[5.2; 92.1]
Germany	9	22.9 ± 5.5	[4.3; 47.8]
Romania	8	5.6 ± 0.9	[1.97; 9.3]
Sweden	10	19.8 ± 6.7	[2.1; 62.2]
Switzerland	8	1.5 ± 0.2	[0.9; 2.6]
overall	43	15.7 ± 3.0	[0.9; 92.1]

Table S2: Grassland management information. Detailed management information for the respective grasslands in the respective years was not available for most sites. The classification is based on farmer interviews and observations during the fieldwork as well as in preceding and subsequent years.

grassland ID	management	available detailed information
Bulgaria 02	extensively grazed	
Bulgaria 03	mown and / or extensively grazed	
Bulgaria 04	grazed at moderate intensity	
Bulgaria 05	extensively grazed	
Bulgaria 06	grazed at moderate intensity	
Bulgaria 07	grazed at moderate intensity	
Bulgaria 08	grazed at moderate intensity	
Bulgaria 09	grazed at moderate intensity	more intensively grazed
Germany 01	mown and / or extensively grazed	mown once, afterwards extensively grazed (autumn)
Germany 02	mown and / or extensively grazed	mown once, afterwards extensively grazed (sheep for short period; vagrant shepherd)
Germany 03	mowing	mown once
Germany 04	mowing	mown twice
Germany 05	extensively grazed	grazed three times with sheep for short period (vagrant shepherd)
Germany 06	unmanaged	
Germany 07	mown and / or extensively grazed	mown twice or grazed with < 1 livestock units
Germany 08	grazed at moderate intensity	grazed with ~ 2 livestock units
Germany 09	grazed at moderate intensity	grazed with ~ 4.6 livestock units
Romania 01	extensively grazed	
Romania 02	grazed at moderate intensity	
Romania 03	extensively grazed	
Romania 04	extensively grazed	
Romania 05	mown	hay meadow, mown once a year in July
Romania 06	extensively grazed	
Romania 07	grazed at moderate intensity	
Romania 08	grazed at moderate intensity	more intensively grazed
Sweden 01	extensively grazed	
Sweden 02	extensively grazed	
Sweden 03	extensively grazed	
Sweden 04	extensively grazed	
Sweden 05	extensively grazed	
Sweden 06	extensively grazed	
Sweden 07	extensively grazed	
Sweden 08	mown	
Sweden 09	extensively grazed	
Sweden 10	extensively grazed	
Switzerland 01	mown	mown once in late June to early July
Switzerland 02	mown	mown once between early July and mid-August
Switzerland 03	mown	mown once between early July and mid-August
Switzerland 04	mown	mown once in late June to early July
Switzerland 05	mown	mown once in late June to early July
Switzerland 06	mown	mown once between early July and mid-August
Switzerland 07	mown	mown once between early July and mid-August
Switzerland 08	mown	mown once in late June to early July

Table S3: Permanent grassland cover (amount in ha and proportion of the landscape covered in %) in landscapes at a radius of 1000 m and 2000 m around the selected oilseed rape fields (OSR) and permanent grassland patches as well as in the combined landscapes used for beta-diversity assessments (mean ± SE). See also Fig. S2 for a graphical representation.

	OSR fields				grassland plots				combined			
scale	1000		2000		1000		2000		1000		2000	
measure	amount	proportion	amount	proportion	amount	proportion	amount	proportion	amount	proportion	amount	proportion
Bulgaria	32.4 ± 2.3	10.3 ± 0.7	153.3 ± 10.1	12.2 ± 0.8	53.4 ± 4.6	17.0 ± 1.5	182.5 ± 13.0	14.5 ± 1.0	42.9 ± 3.2	13.7 ± 1.0	167.9 ± 11.2	13.4 ± 0.9
Germany	48.5 ± 3.0	15.4 ± 1.0	202.0 ± 13.1	16.1 ± 1.0	59.1 ± 5.2	18.8 ± 1.7	225.1 ± 13.3	17.9 ± 1.1	53.8 ± 4.0	17.1 ± 1.3	213.5 ± 13.1	17.0 ± 1.0
Romania	81.2 ± 6.2	25.8 ± 2.0	371.5 ± 26.8	29.6 ± 2.1	89.7 ± 8.0	28.5 ± 2.6	388.6 ± 30.6	30.9 ± 2.4	85.4 ± 7.0	27.2 ± 2.2	380.1 ± 28.6	30.2 ± 2.3
Sweden	72.9 ± 5.9	23.2 ± 1.9	293.4 ± 21.4	23.3 ± 1.7	67.8 ± 4.7	21.6 ± 1.5	281.4 ± 19.0	22.4 ± 1.5	70.4 ± 5.1	22.4 ± 1.6	287.4 ± 20.1	22.9 ± 1.6
Switzerland	83.0 ± 4.4	26.4 ± 1.4	309.5 ± 13.1	24.6 ± 1.0	83.4 ± 3.3	26.6 ± 1.1	319.3 ± 11.9	25.4 ± 0.9	83.2 ± 3.8	26.5 ± 1.2	314.4 ± 12.4	25.0 ± 1.0
Total	63.7 ± 5.4	20.3 ± 1.7	265.7 ± 20.8	21.1 ± 1.7	70.3 ± 5.5	22.4 ± 1.8	278.2 ± 20.9	22.1 ± 1.7	67.0 ± 5.2	21.3 ± 1.7	272.0 ± 20.7	21.6 ± 1.6

Supplementary Note 1: Results of the analyses at the 1000 m spatial scale

Most of the results are the same as those obtained from the analyses at the 2000 m spatial scale depicted in the main text. In the following, we only display results that differ from those already presented in the main text, i.e. where results differ between the spatial scales.

Effects of habitat type and landscape grassland amount on observed richness and effective number of species were constant across taxa at both spatial scales (Fig. S4, Tables S5 & S6), but interactions between landscape grassland amount and country detected at the 2000 m scale were not found at the 1000 m scale (Figs. S6 & S9; Tables S5 & S6).

The composition of species assemblages was similarly affected by the predictors at both spatial scales in all taxa but wild bees (Table S7). Here, while habitat type was found to shape assemblages at the 2000 m scale this effect was only marginally significant at the 1000 m scale but instead assemblage composition was affected by landscape grassland amount at this scale (Table S7).

Trends for beta-diversity between the paired grasslands and oilseed rape fields varied, however, than between spatial scales. The negative relationships between nestedness and grassland amount in plants and between turnover and grassland amount in wild bees detected at the 2000 m scale were only marginally significant at the 1000 m scale and the positive relationship between nestedness and grassland amount detected in wild bees at the 2000 m scale was not detected at all at the 1000 m scale (Fig. S15, Table S8). In carabids, we found a negative relationship between nestedness and grassland amount at the 1000 m scale that was not found at the 2000 m scale (Fig. S15, Table S8). While relationships between turnover and grassland amount across countries were similar at both spatial scales, the interactive effect between grassland amount and country on the nestedness of wild bees detected at the 2000 m scale was not significant at the 1000 m scale but found an interactive effect between grassland amount and country on the nestedness of butterflies that was not found at the 2000 m scale (Table S8, Figs. S14 & S15).

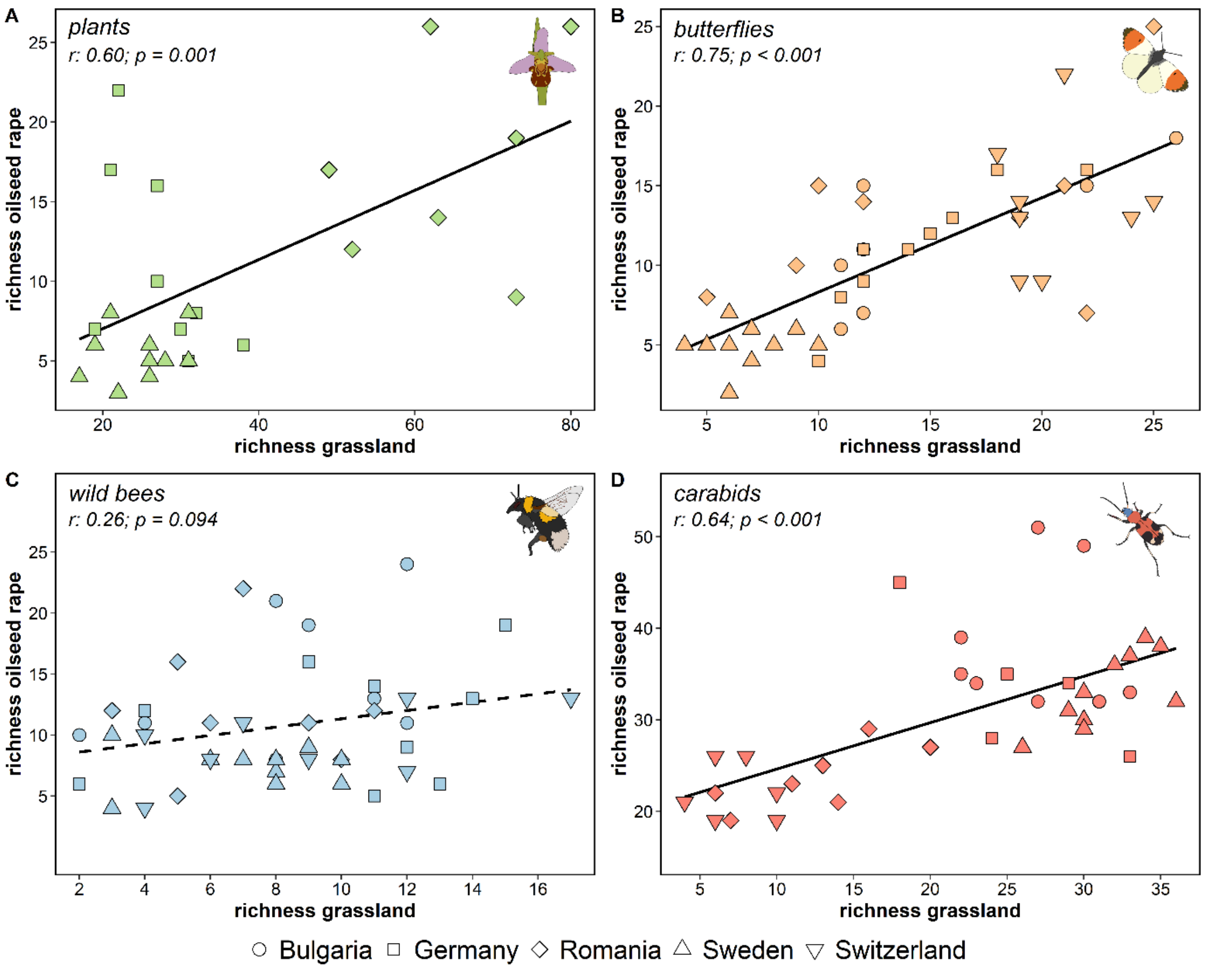

Fig. S3: Relationship between species richness of plants (A; n = 26), butterflies (B; n = 43), wild bees (C; n = 43) and carabids (D; n = 36) in oilseed rape fields and species richness of the same taxon in the adjacent permanent grasslands across all landscapes. Black solid lines indicate statistically significant positive correlations, black dashed lines non-significant relations (lines represent predictions from simple linear regressions). Pearson's correlation coefficient r and p-values given in the upper left corner of each panel. Species richness in oilseed rape fields was positively correlated to the richness in the paired grasslands for plants (Pearson: $r = 0.60$, $p = 0.001$), butterflies (Pearson: $r = 0.75$, $p < 0.001$) and carabids (Pearson: $r = 0.64$, $p < 0.001$), but not for wild bees (Pearson: $r = 0.26$, $p = 0.094$).

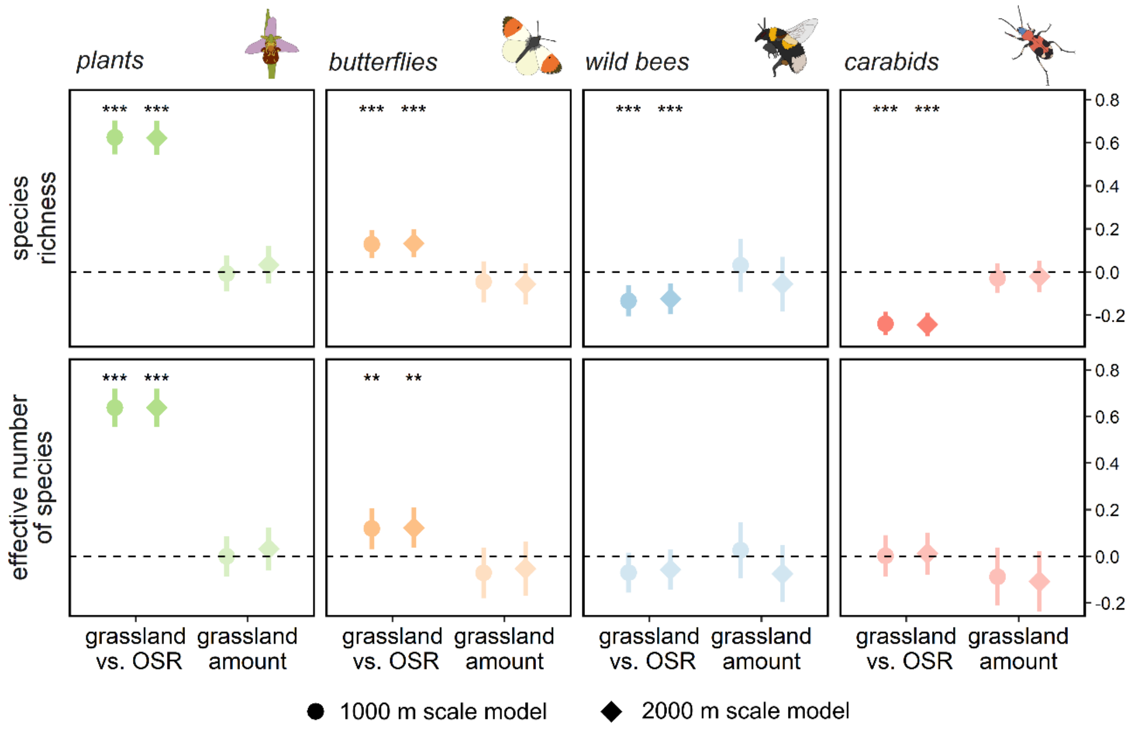

Fig. S4: Model coefficients for habitat type ('grassland vs. OSR'; positive values indicate a higher value in grasslands) and landscape grassland amount for the species richness and effective number of species in plants (n = 52), butterflies (n = 86), wild bees (n = 86) and carabids (n = 72) at two spatial scales, 1000 m and 2000 m. Coefficients with 95% confidence intervals. Coefficients are on the log-scale. (*) indicates $p < 0.1$, * $p < 0.05$, ** $p < 0.01$, *** $p < 0.001$. For statistics, see text and Tables S5, S6 & S8.

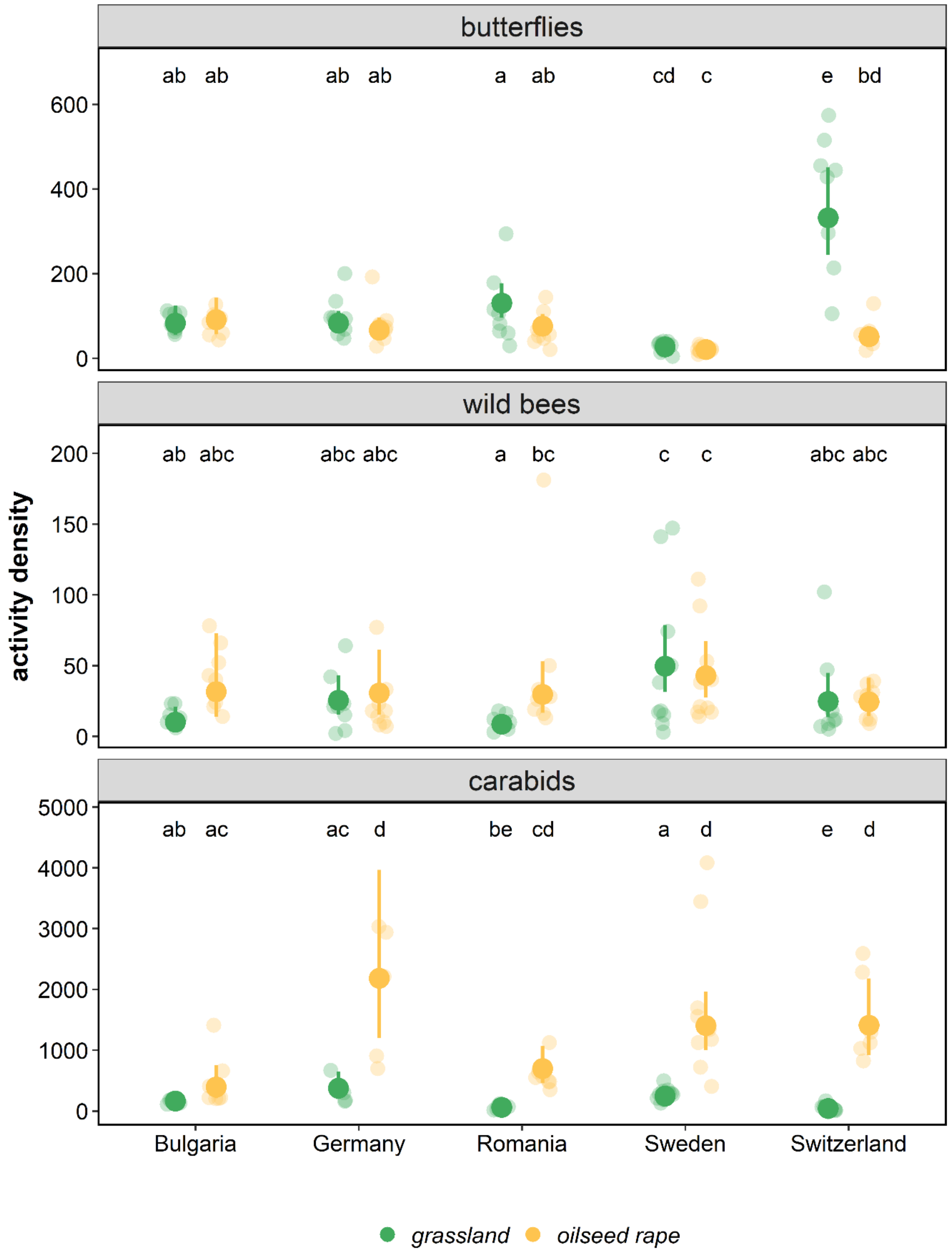

Fig. S5: Activity densities of butterflies (n = 43), wild bees (n = 43) and carabids (n = 36) in relation to habitat type and country (estimated marginal means with 95% confidence intervals). Translucent symbols are actual data points (in carabids not standardised for sampling effort). Models were fitted as described for species richness and used negative binomial residual distributions (detailed results not shown). The habitat type × country interaction shown here was the only significant effect found (apart from effects of habitat type and country) in all three taxa (depicted marginal means were extracted from the model at the 2000 m spatial scale). Different letters represent statistically significant differences ($p < 0.05$) obtained using Tukey post-hoc contrasts in the library 'emmeans'.

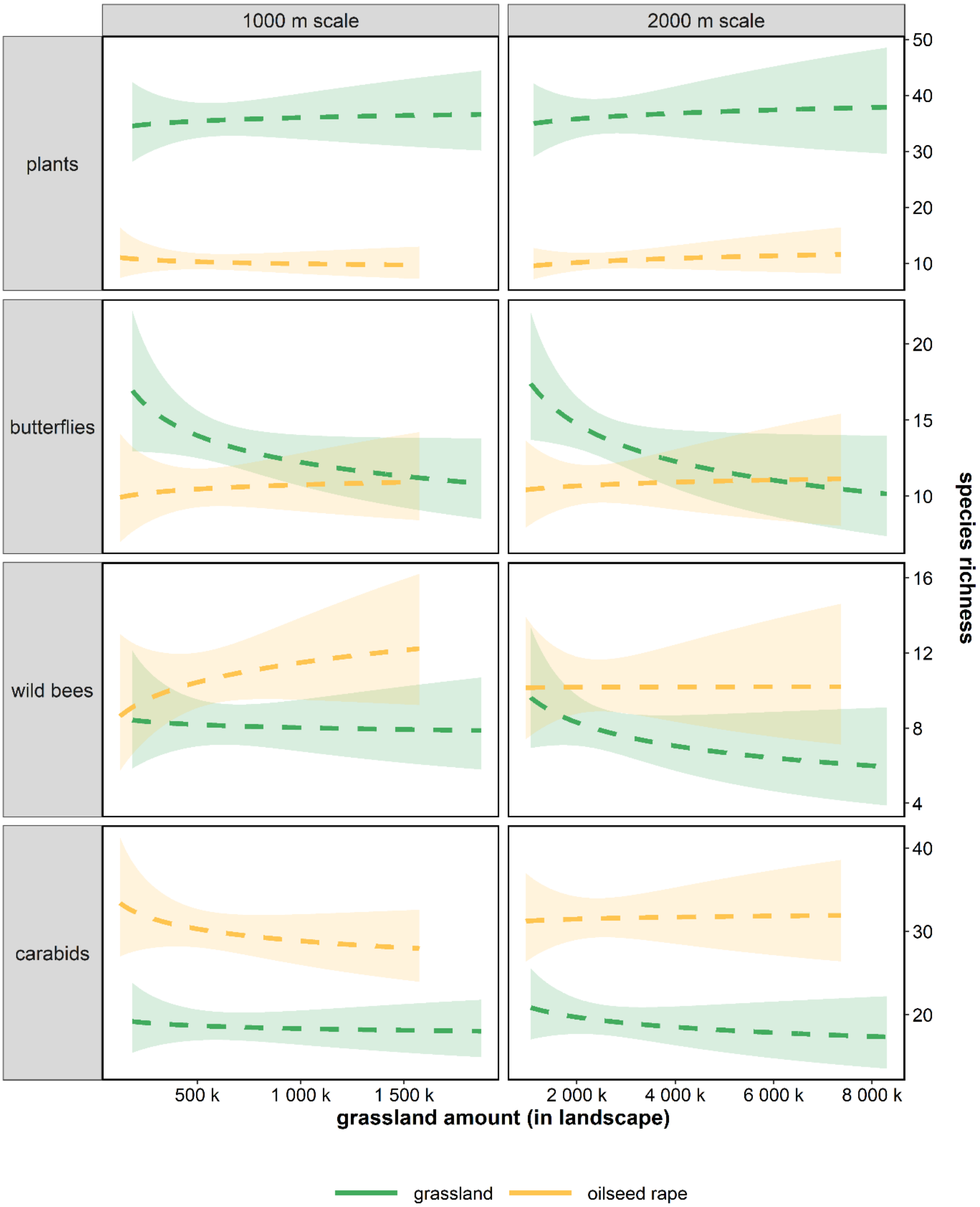

Fig. S6: Model predictions (estimated marginal means) for species richness in relation to grassland amount and habitat type at the spatial scales of 1000 m and 2000 m for plants (each habitat type n = 26), butterflies (each habitat type n = 43), wild bees (each habitat type n = 43) and carabids (each habitat type n = 36; marginal predictions with 95% confidence interval). Solid lines indicate statistically significant interactions ($p < 0.05$), dashed lines indicate statistically non-significant interactions ($p > 0.05$). For methods, see main text, for statistics, see Table S5.

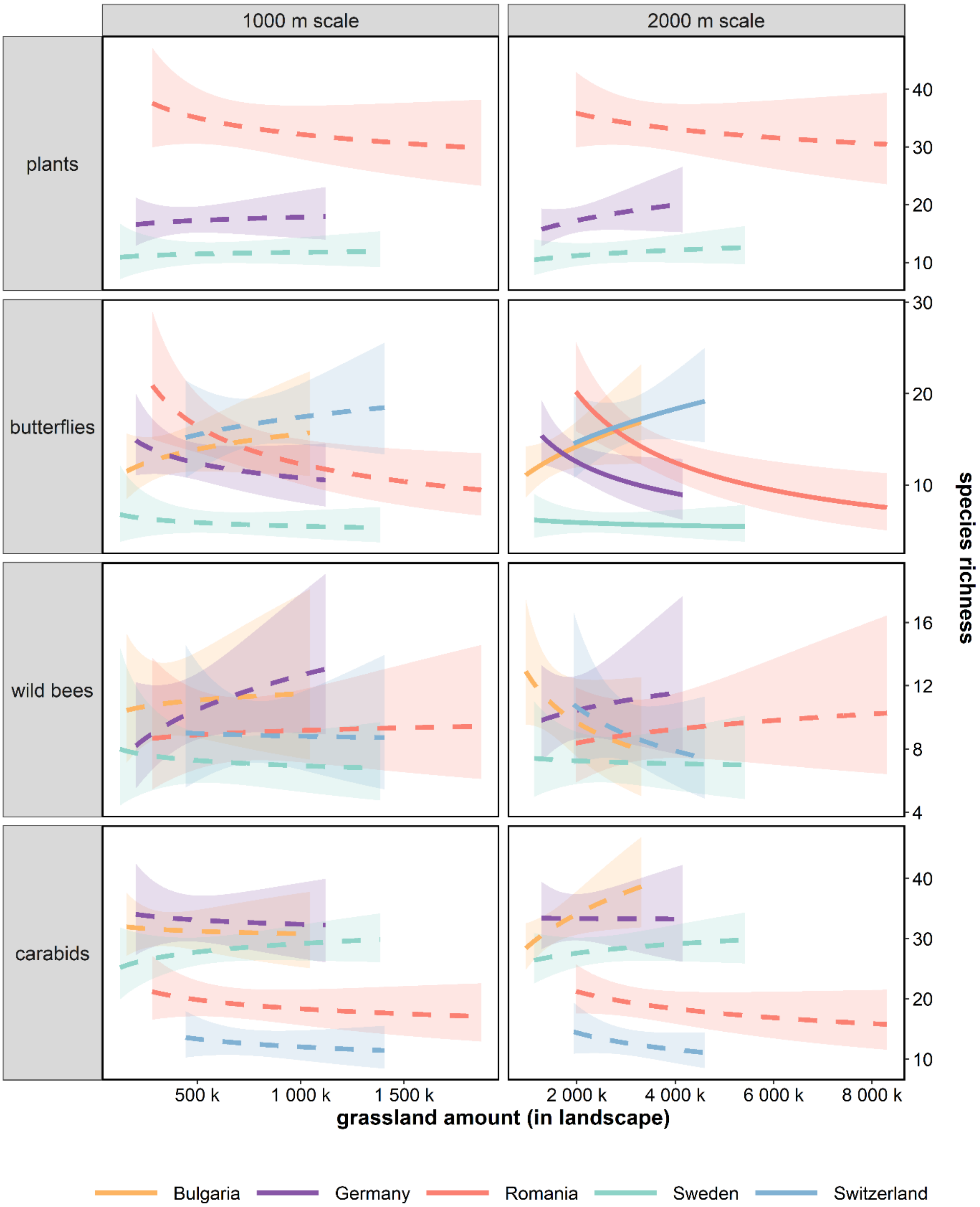

Fig. S7: Model predictions (estimated marginal means) for species richness in relation to grassland amount and country at the spatial scales of 1000 m and 2000 m for plants (n = 52), butterflies (n = 86), wild bees (n = 86) and carabids (n = 72; marginal predictions with 95% confidence interval). Solid lines indicate statistically significant interactions ($p < 0.05$), dashed lines indicate statistically non-significant interactions ($p > 0.05$). 'k' indicates 1000. For methods, see main text, for statistics, see Table S5.

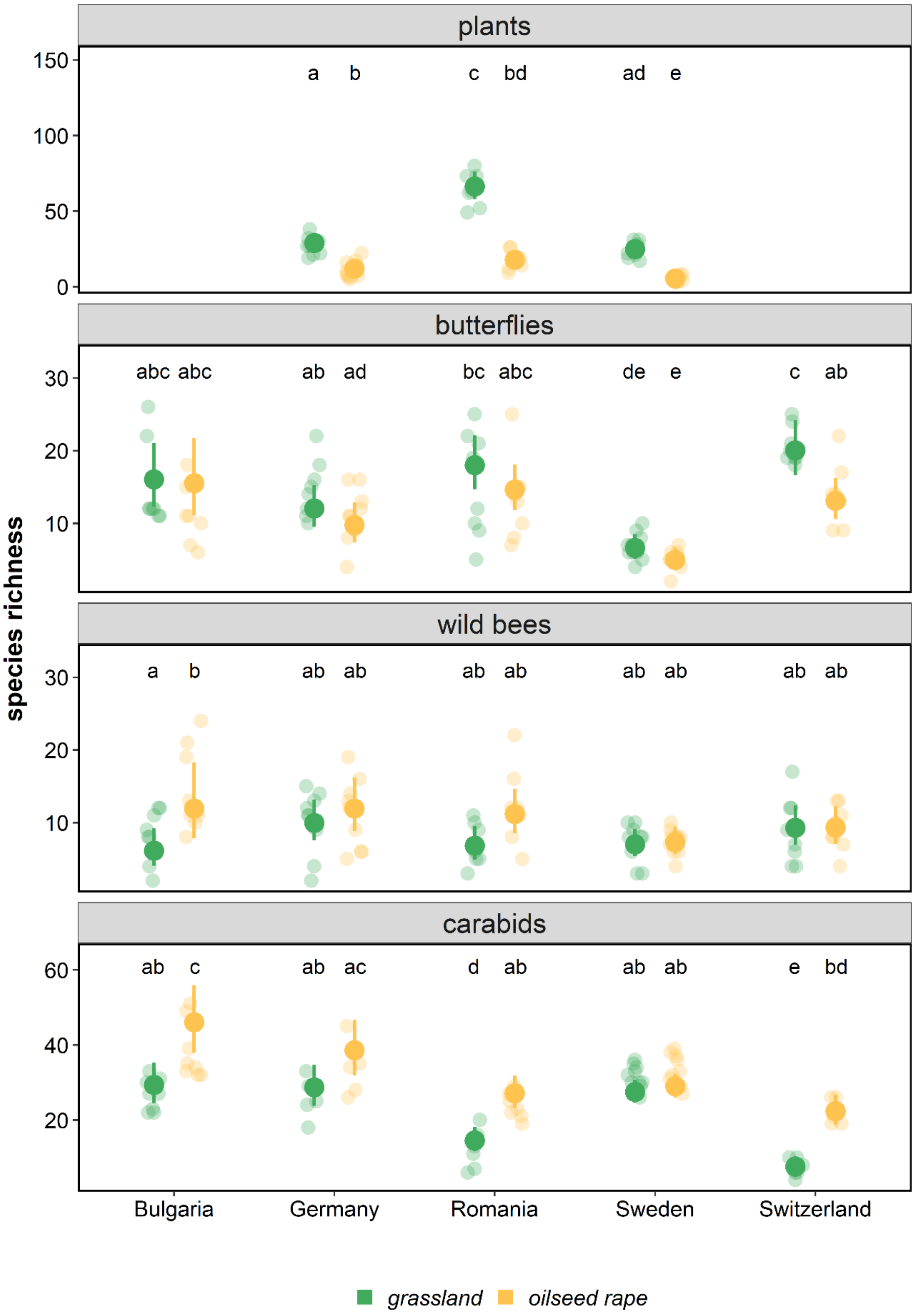

Fig. S8: Model predictions (estimated marginal means) at the 2000 m scale for the species richness in relation to habitat type and country for plants (each habitat type n = 26), butterflies (each habitat type n = 43), wild bees (each habitat type n = 43) and carabids (each habitat type n = 36; estimated marginal means with 95% confidence interval). Translucent points represent the actual data points (in case of carabids not corrected for sampling effort). Different letters represent statistically significant differences ($p < 0.05$) obtained using Tukey post-hoc contrasts in the library 'emmeans'. For methods, see main text, for statistics, see Table S5.

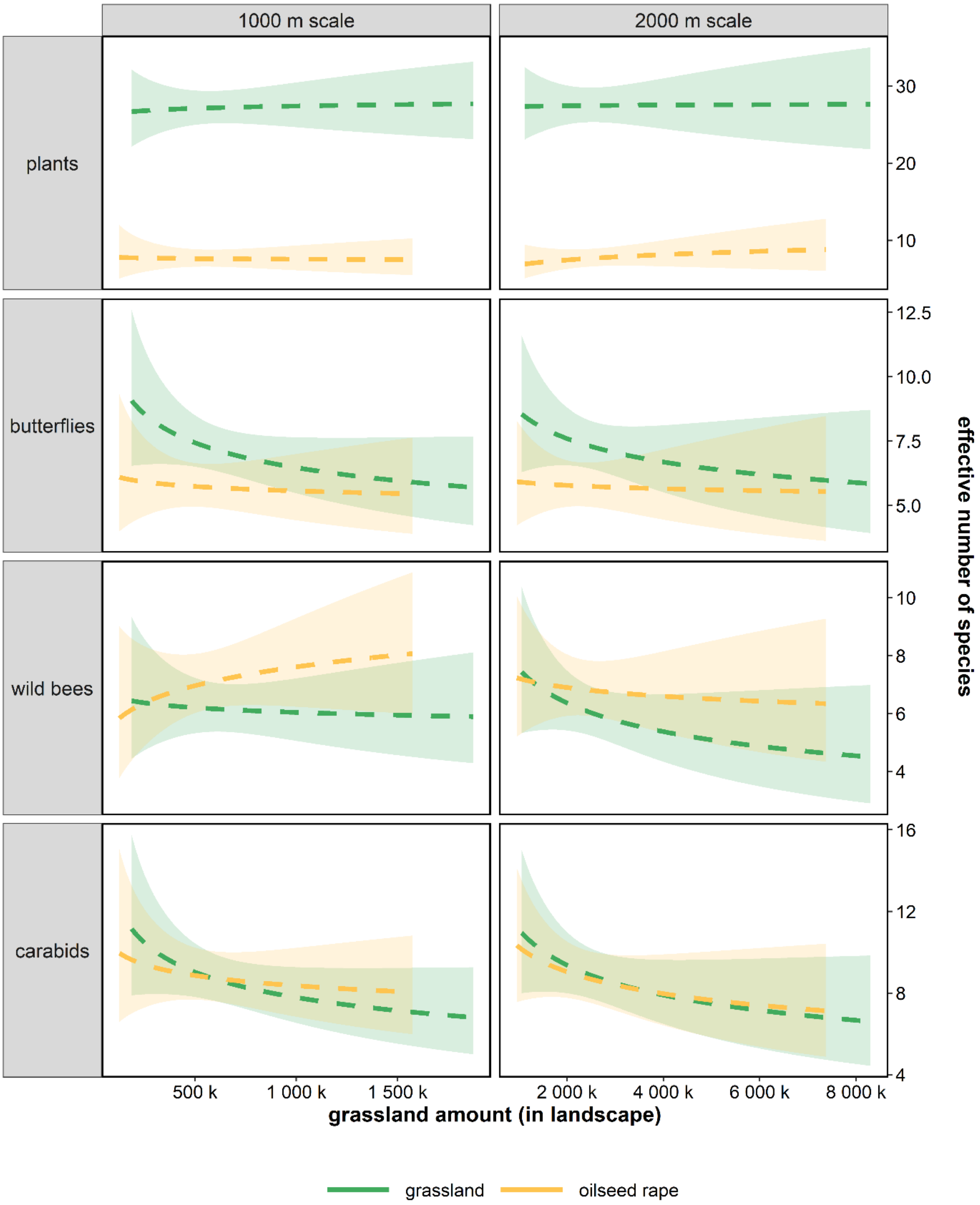

Fig. S9: Model predictions (estimated marginal means) for the effective number of species in relation to grassland amount and habitat type at the spatial scales of 1000 m and 2000 m for plants (each habitat type n = 26), butterflies (each habitat type n = 43), wild bees (each habitat type n = 43) and carabids (each habitat type n = 36; marginal predictions with 95% confidence interval). Solid lines indicate statistically significant interactions ($p < 0.05$), dashed lines indicate statistically non-significant interactions ($p > 0.05$). For methods, see main text, for statistics, see Table S6.

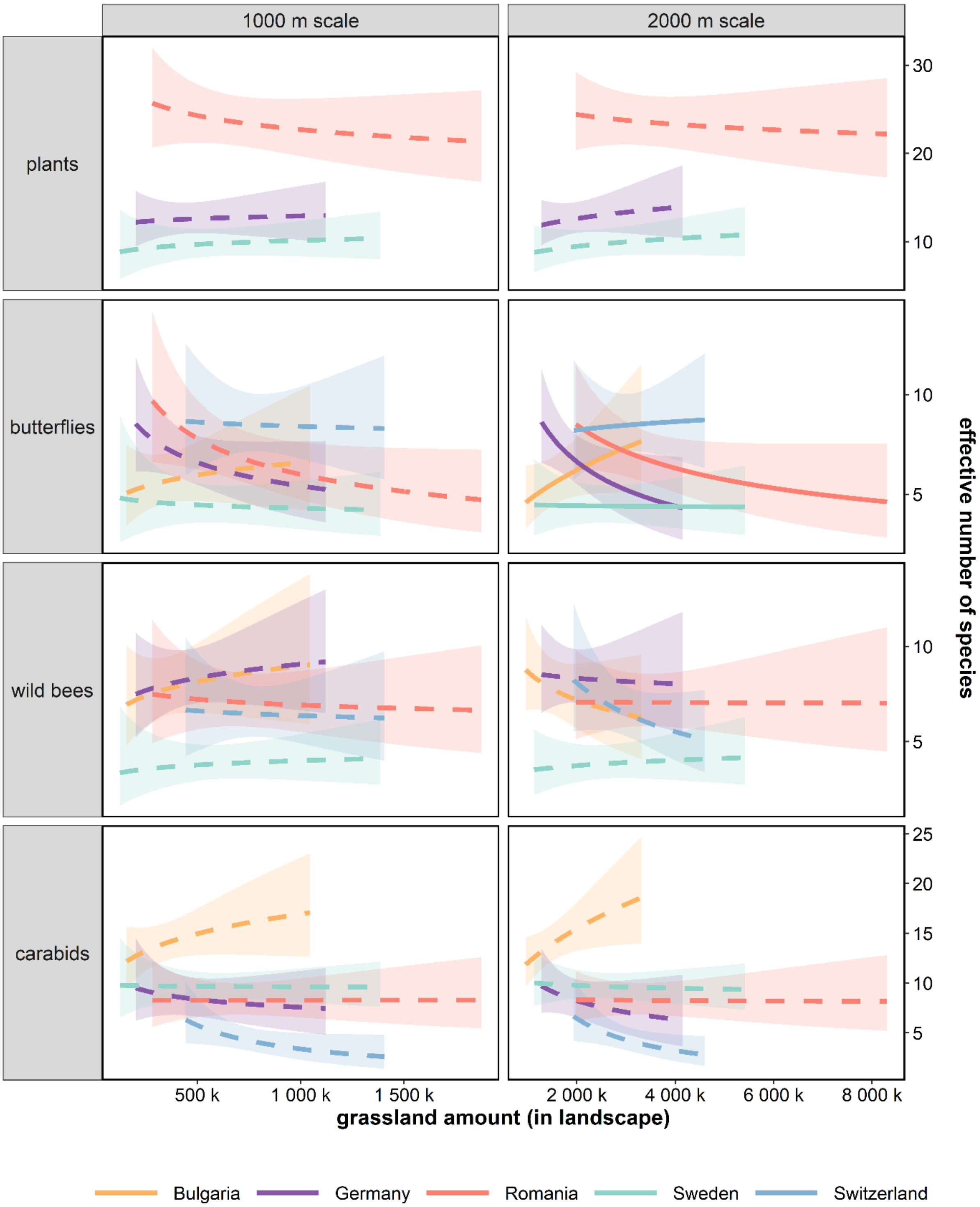

Fig. S10: Model predictions (estimated marginal means) for the effective number of species in relation to grassland amount and country at the spatial scales of 1000 m and 2000 m for plants (n = 52), butterflies (n = 86), wild bees (n = 86) and carabids (n = 72; marginal predictions with 95% confidence interval). Solid lines indicate statistically significant interactions ($p < 0.05$), dashed lines indicate statistically non-significant interactions ($p > 0.05$). 'k' indicates 1000. For methods, see main text, for statistics, see Table S6.

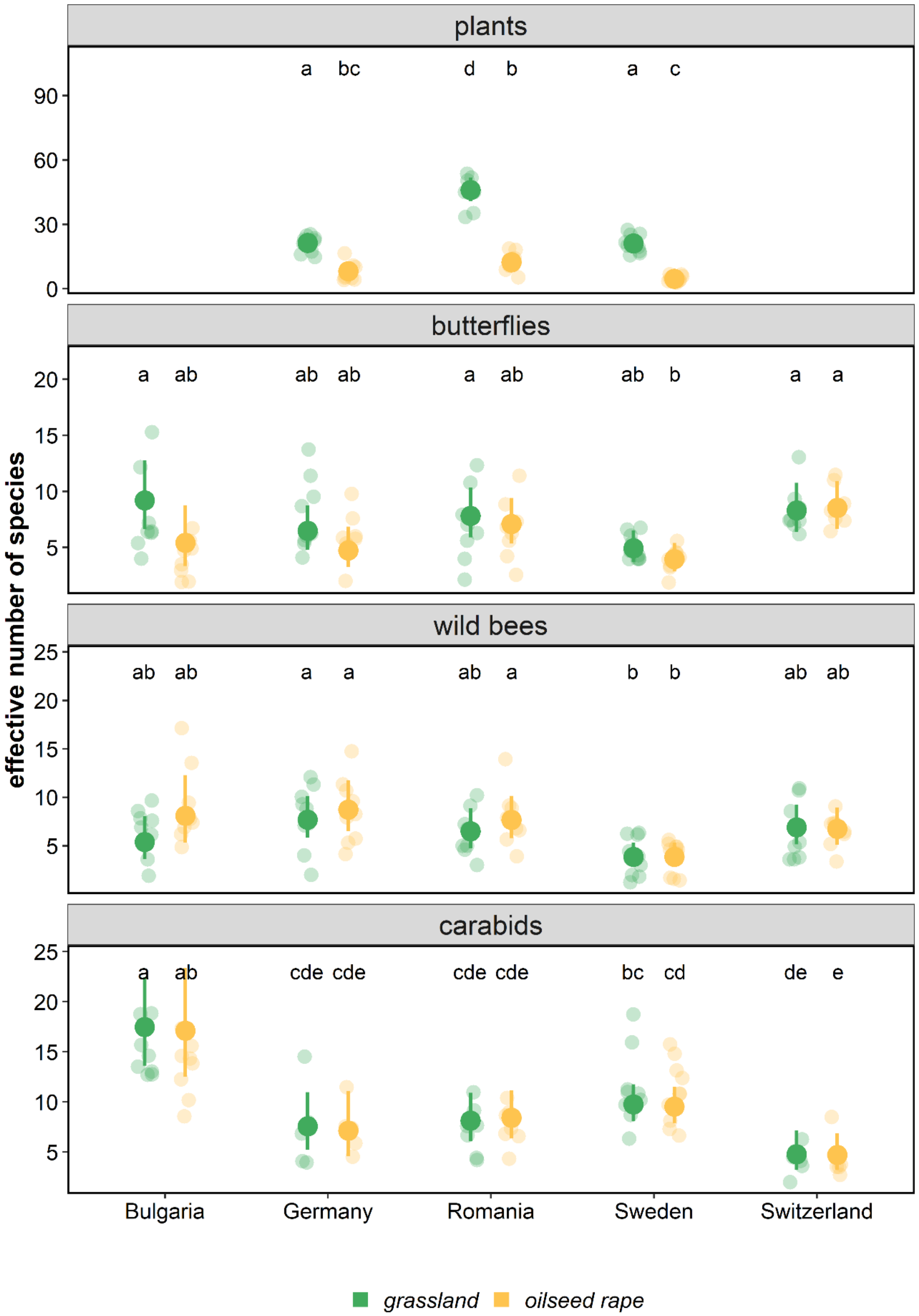

Fig. S11: Model predictions (estimated marginal means) for the effective number of species in relation to habitat type and country at the 2000 m spatial scale for plants (each habitat type n = 26), butterflies (each habitat type n = 43), wild bees (each habitat type n = 43) and carabids (each habitat type n = 36; marginal predictions with 95% confidence interval). Translucent points represent the actual data points (in case of carabids not corrected for sampling effort). Different letters represent statistically significant differences ($p < 0.05$) obtained using Tukey post-hoc contrasts in the library 'emmeans'. For methods, see main text, for statistics, see Table S6.

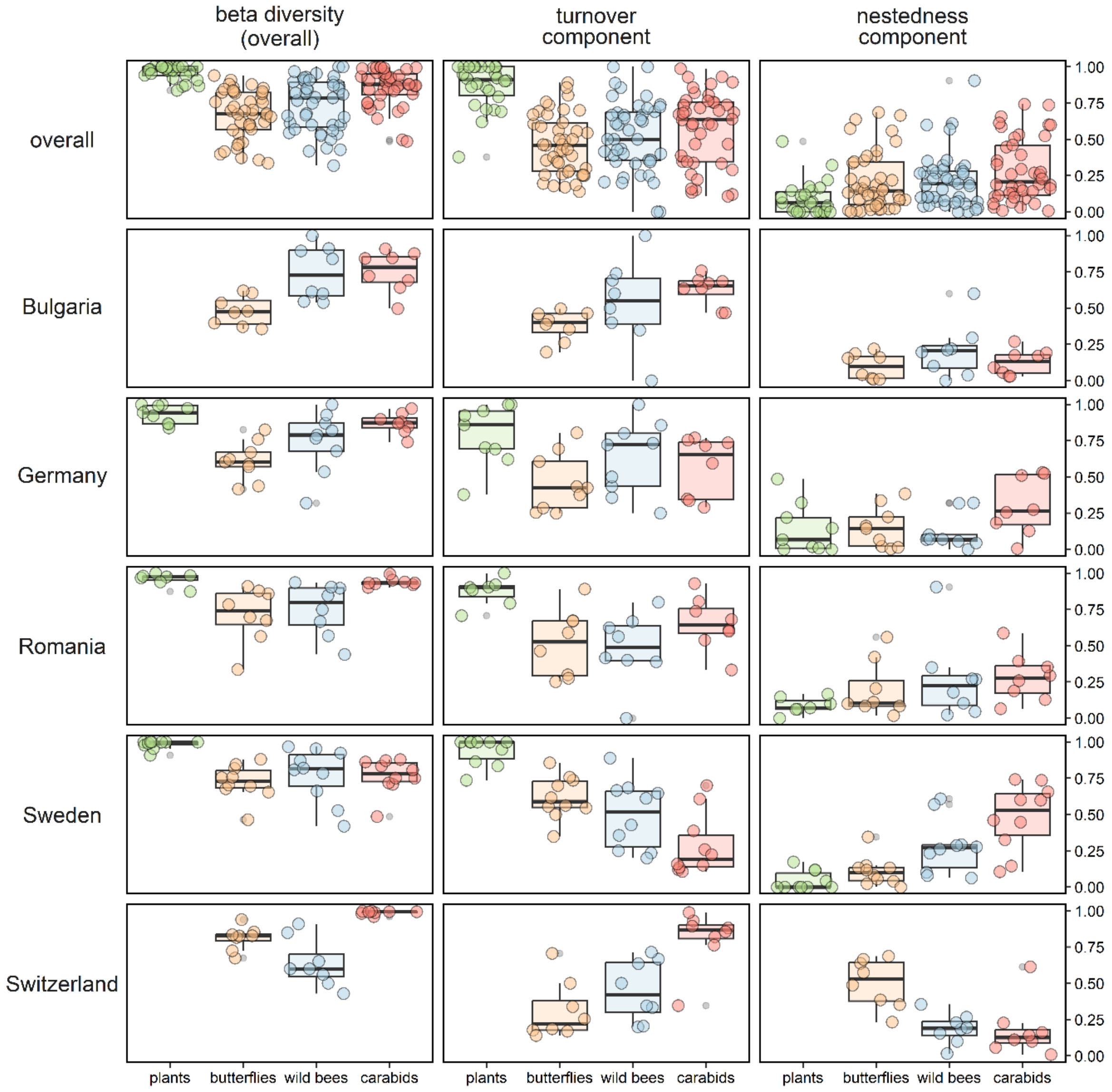

Fig. S12: Overall Bray-Curtis beta-diversity between paired grasslands and oilseed rape fields and its turnover and nestedness components for plants (n = 26), butterflies (n = 43), wild bees (n = 43) and carabids (n = 36) across all countries and for each country separately. Points represent the actual data points. Boxes show the 25th percentile, median and the 75th percentile values, and whiskers extend to the furthest data point within 1.5 times of the interquartile range.

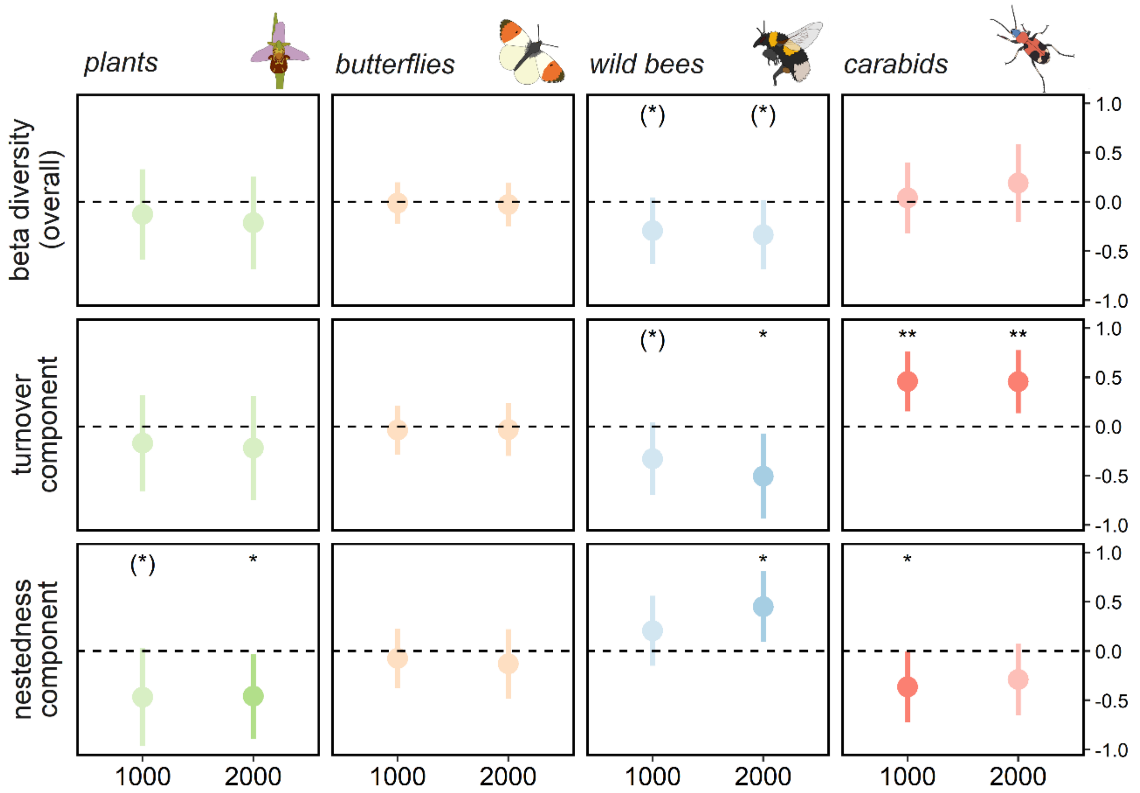

Fig. S13: Model coefficients for the effects of grassland amount at two spatial scales, 1000 m and 2000 m, on overall beta-diversity (Bray-Curtis dissimilarity) and its turnover and nestedness components in plants (n = 26), butterflies (n = 43), wild bees (n = 43) and carabids (n = 36). Coefficients with 95% confidence intervals. Coefficients are on the logit-scale. (*) indicates p < 0.1, * p < 0.05, ** p < 0.01, *** p < 0.001. For statistics, see text and Table S8 & S9.

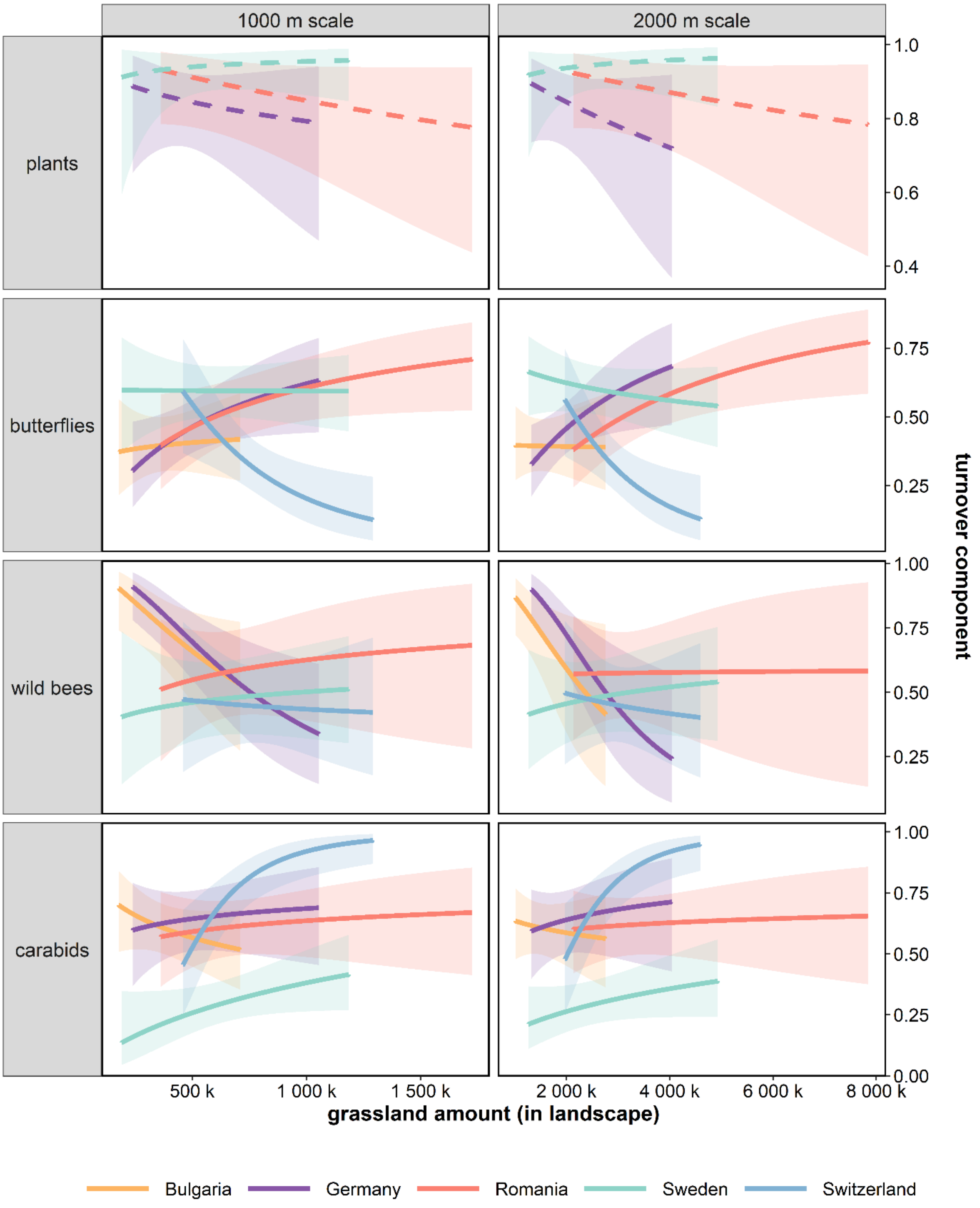

Fig. S14: Model predictions (estimated marginal means) for the turnover component of beta diversity in relation to grassland amount at spatial scales of 1000 m and 2000 m and country for plants (n = 26), butterflies (n = 43), wild bees (n = 43) and carabids (n = 36; marginal predictions with 95% confidence interval). Solid lines indicate statistically significant interactions ($p < 0.05$), dashed lines indicate statistically non-significant interactions ($p > 0.05$). 'k' indicates 1000. For methods, see main text, for statistics, see Table S8.

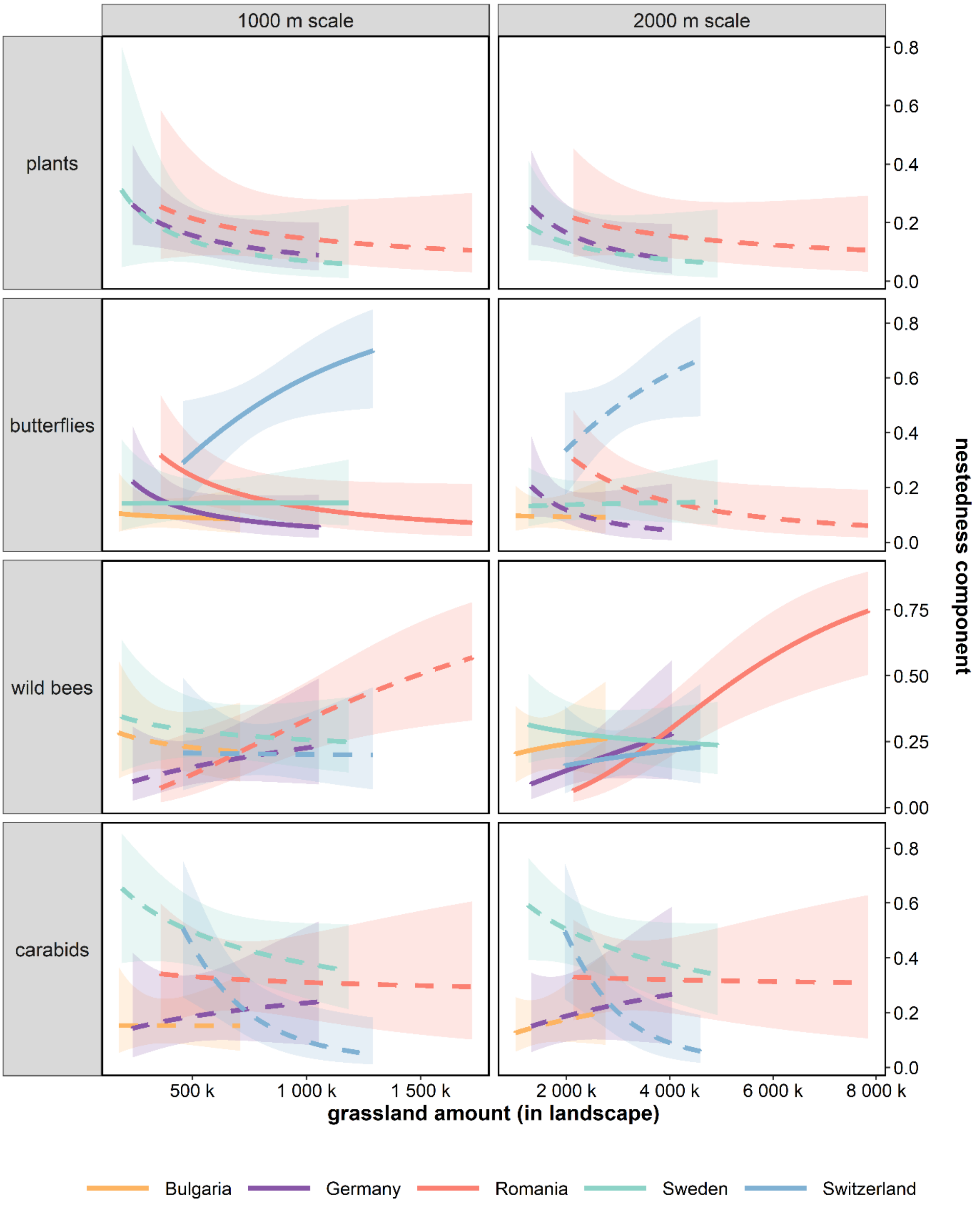

Fig. S15: Model predictions (estimated marginal means) for the nestedness component of beta diversity in relation to grassland amount at spatial scales of 1000 m and 2000 m and country for plants (n = 26), butterflies (n = 43), wild bees (n = 43) and carabids (n = 36; marginal predictions with 95% confidence interval). Solid lines indicate statistically significant interactions ($p < 0.05$), dashed lines indicate statistically non-significant interactions ($p > 0.05$). 'k' indicates 1000. For methods, see main text, for statistics, see Table S8.

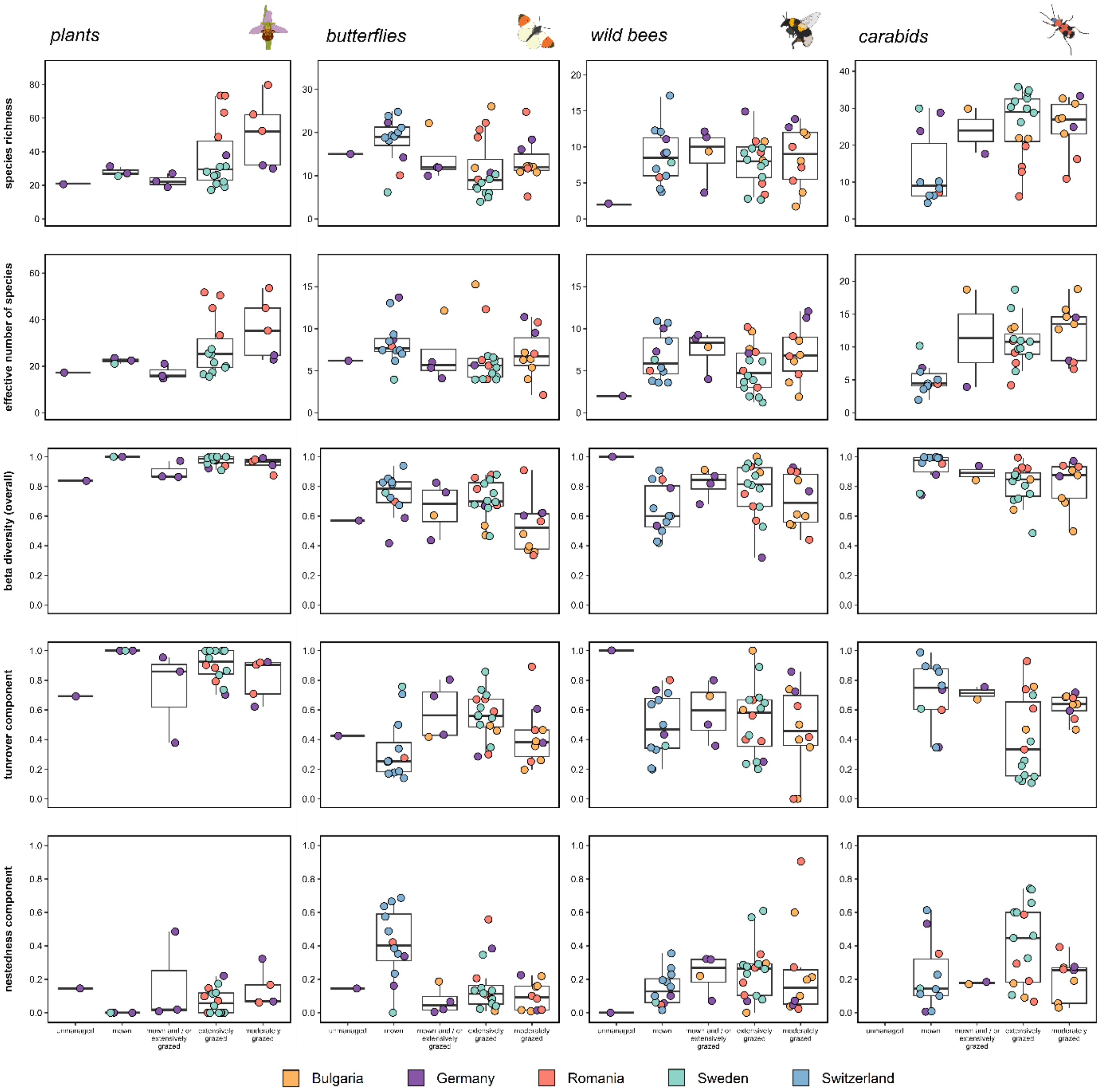

Fig. S16: Species richness and effective number of species of plants (n = 26), butterflies (n = 43), wild bees (n = 43) and carabids (n = 36) recorded in grasslands as well as their overall beta diversity and its turnover and nestedness components between grasslands and paired oilseed rape fields in relation to grassland management. Boxes show the 25th percentile, median and the 75th percentile values, and whiskers extend to the furthest data point within 1.5 times of the interquartile range. Points represent individual grasslands with different colours depending on the country (see legend). Management categories are coarse estimations as no detailed information was available. No statistics were performed due to unequal and in parts low sample sizes in the categories, but the patters suggest that grassland management was overall not an important factor for the diversity harboured therein or for the beta diversity between paired grasslands and oilseed rape fields.

Table S4: Species richness of plants, butterflies, wild bees and carabids (total and average) for each habitat and country. OSR = winter oilseed rape field; SD = standard deviation. Averages and standard deviations rounded to one decimal.

	plants		butterflies		wild bees		carabids	
	**total**	**average ± SD**	**total**	**average ± SD**	**total**	**average ± SD**	**total**	**average ± SD**
**Bulgaria**								
OSR			31	11.6 ± 4.1	55	14.6 ± 5.9	99	38.1 ± 7.7
grassland			38	14.8 ± 5.8	44	8.2 ± 3.7	81	26.9 ± 4.3
total			42	13.2 ± 5.1	76	11.4 ± 5.8	124	32.5 ± 8.4
**Germany**								
OSR	47	10.9 ± 6.0	27	11.1 ± 3.8	55	11.1 ± 4.9	67	31.2 ± 6.7
grassland	107	27.4 ± 6.1	34	14.4 ± 3.8	47	10.1 ± 4.4	68	24.0 ± 6.5
total	127	19.2 ± 10.3	36	12.8 ± 4.1	71	10.6 ± 4.6	89	27.6 ± 7.4
**Romania**								
OSR	62	17.6 ± 6.6	38	13.4 ± 5.6	49	12.1 ± 5.1	62	24.2 ± 3.6
grassland	182	64.6 ± 11.5	39	15.4 ± 7.3	33	7.0 ± 2.8	45	11.9 ± 4.8
total	213	41.1 ± 26.0	50	14.4 ± 6.4	61	9.6 ± 4.8	75	18.1 ± 7.6
**Sweden**								
OSR	15	5.4 ± 1.6	11	5.0 ± 1.3	25	7.4 ± 1.7	69	33.2 ± 4.1
grassland	80	24.7 ± 4.8	18	6.8 ± 1.8	30	7.2 ± 2.5	77	31.5 ± 3.1
total	82	15.1 ± 10.5	18	5.9 ± 1.8	38	7.3 ± 2.1	87	32.4 ± 3.6
**Switzerland**								
OSR			35	13.9 ± 4.2	32	9.2 ± 3.1	45	21.4 ± 3.5
grassland			34	20.6 ± 2.6	35	8.9 ± 4.5	30	7.8 ± 2.7
total			41	17.2 ± 4.9	50	9.1 ± 3.8	60	14.6 ± 7.7

Table S5: Type III Wald χ^2 tests for generalised linear mixed effects models of species richness in relation to habitat type (semi-natural grassland vs. oilseed rape field), country and landscape permanent grassland cover at the 1000 m and 2000 m scales (separate models for the two scales) as well as their interactions across the four taxonomic groups vascular plants (n = 26), butterflies (n = 43), wild bees (n = 43), and carabids (n = 36). (*) indicates p < 0.10, * indicates p < 0.05, ** indicates p < 0.01, *** indicates p < 0.001. Df = degrees of freedom (numerator, denominator), F = F-value, p = p-value, R^2_m = marginal R^2 value.

Response	residual distribution	Df	χ^2	p	R^2_m
**Plants**					
1000 m scale					
habitat type	negative binomial	1	242.07	**< 0.001 *****	0.92
grassland amount		1	0.03	0.860	
country		2	123.69	**< 0.001 *****	
grassland amount × habitat type		1	0.26	0.612	
grassland amount × country		2	1.43	0.489	
habitat type × country		2	7.89	**0.019 ***	
2000 m scale					
habitat type	negative binomial	1	243.75	**< 0.001 *****	0.92
grassland amount		1	0.57	0.448	
country		2	112.27	**< 0.001 *****	
grassland amount × habitat type		1	0.11	0.735	
grassland amount × country		2	2.75	0.252	
habitat type × country		2	9.43	**0.009 ****	
**Butterflies**					
1000 m scale					
habitat type	negative binomial	1	15.02	**< 0.001 *****	0.65
grassland amount		1	0.85	0.356	
country		4	66.32	**< 0.001 *****	
grassland amount × habitat type		1	2.75	**0.097 (*)**	
grassland amount × country		4	8.33	**0.080 (*)**	
habitat type × country		4	3.76	0.440	
2000 m scale					
habitat type	negative binomial	1	16.69	**< 0.001 *****	0.68
grassland amount		1	1.29	0.255	
country		4	81.25	**< 0.001 *****	
grassland amount × habitat type		1	2.86	**0.091 (*)**	
grassland amount × country		4	18.39	< 0.001 ***	
habitat type × country		4	3.58	0.466	
**Wild bees**					
1000 m scale					
habitat type	negative binomial	1	13.21	**< 0.001 *****	0.28
grassland amount		1	0.25	0.615	
country		4	9.89	**0.042 ***	
grassland amount × habitat type		1	1.20	0.274	
grassland amount × country		4	1.65	0.800	
habitat type × country		4	12.28	**0.015 ***	
2000 m scale					
habitat type	negative binomial	1	11.64	**< 0.001 *****	0.32
grassland amount		1	0.75	0.385	
country		4	7.93	**0.094 (*)**	
grassland amount × habitat type		1	1.53	0.216	
grassland amount × country		4	3.54	0.472	
habitat type × country		4	11.47	**0.022 ***	
**Carabids**					
1000 m scale					
habitat type	negative binomial	1	73.68	**< 0.001 *****	0.33
grassland amount		1	0.70	0.404	
country		4	83.92	**< 0.001 *****	
grassland amount × habitat type		1	0.22	0.637	
grassland amount × country		4	2.57	0.633	
habitat type × country		4	38.48	**< 0.001 *****	
2000 m scale					
habitat type	Poisson	1	77.48	**< 0.001 *****	0.34
grassland amount		1	0.30	0.586	
country		4	80.01	**< 0.001 *****	
grassland amount × habitat type		1	0.80	0.372	
grassland amount × country		4	8.24	**0.083 (*)**	
habitat type × country		4	37.25	**< 0.001 *****	

Table S6: Type III Wald χ^2 tests for generalised linear mixed effects models of the effective number of species (i.e. the exponent of the Shannon–Wiener diversity index) in relation to habitat type (semi-natural grassland vs. oilseed rape field), country and landscape permanent grassland cover at the 1000 m and 2000 m scales (separate models for the two scales) as well as their interactions across the four taxonomic groups vascular plants (n = 26), butterflies (n = 43), wild bees (n = 43), and carabids (n = 36). (*) indicates p < 0.10, * indicates p < 0.05, ** indicates p < 0.01, *** indicates p < 0.001. Df = degrees of freedom (numerator, denominator), F = F-value, p = p-value, R^2_m = marginal R^2 value.

Response	residual distribution	Df	χ^2	p	R^2_m
**Plants**					
1000 m scale					
habitat type	negative binomial	1	237.39	**< 0.001** ***	0.91
grassland amount		1	< 0.01	0.992	
country		2	85.82	**< 0.001** ***	
grassland amount × habitat type		1	0.04	0.844	
grassland amount × country		2	1.36	0.506	
habitat type × country		2	5.22	**0.073 (*)**	
2000 m scale					
habitat type	negative binomial	1	236.08	**< 0.001** ***	0.91
grassland amount		1	0.46	0.496	
country		2	74.67	**< 0.001** ***	
grassland amount × habitat type		1	0.42	0.514	
grassland amount × country		2	1.60	0.449	
habitat type × country		2	6.39	**0.041** *	
**Butterflies**					
1000 m scale					
habitat type	negative binomial	1	7.02	**0.008** **	0.39
grassland amount		1	1.66	0.198	
country		4	19.25	**< 0.001** ***	
grassland amount × habitat type		1	0.68	0.410	
grassland amount × country		4	4.71	0.318	
habitat type × country		4	2.83	0.586	
2000 m scale					
habitat type	negative binomial	1	7.70	**0.006** **	0.40
grassland amount		1	0.80	0.372	
country		4	20.13	**< 0.001** ***	
grassland amount × habitat type		1	0.41	0.521	
grassland amount × country		4	9.53	**0.049** *	
habitat type × country		4	3.25	0.517	
**Wild bees**					
1000 m scale					
habitat type	negative binomial	1	2.53	0.111	0.38
grassland amount		1	0.18	0.669	
country		4	31.68	**< 0.001** ***	
grassland amount × habitat type		1	0.85	0.357	
grassland amount × country		4	0.79	0.940	
habitat type × country		4	3.51	0.476	
2000 m scale					
habitat type	negative binomial	1	1.74	0.187	0.39
grassland amount		1	1.46	0.227	
country		4	27.50	**< 0.001** ***	
grassland amount × habitat type		1	0.63	0.429	
grassland amount × country		4	2.57	0.633	
habitat type × country		4	2.52	0.640	
**Carabids**					
1000 m scale					
habitat type	negative binomial	1	< 0.01	0.973	0.15
grassland amount		1	1.92	0.166	
country		4	44.05	**< 0.001** ***	
grassland amount × habitat type		1	0.72	0.397	
grassland amount × country		4	5.70	0.222	
habitat type × country		4	0.11	0.999	
2000 m scale					
habitat type	negative binomial	1	0.07	0.790	0.16
grassland amount		1	2.67	0.103	
country		4	45.58	**< 0.001** ***	
grassland amount × habitat type		1	0.12	0.730	
grassland amount × country		4	10.39	**0.034** *	
habitat type × country		4	0.09	0.999	

Table S7: PERMANOVA analyses of species assemblages (Bray-Curtis distances) in relation to habitat type (semi-natural grassland vs. oilseed rape field), country and landscape permanent grassland cover at the 1000 m and 2000 m scales (separate models for the two scales) as well as their interactions across the four taxonomic groups vascular plants (n = 26), butterflies (n = 43), wild bees (n = 43), and carabids (n = 36).. (*) indicates p < 0.10, * indicates p < 0.05, ** indicates p < 0.01, *** indicates p < 0.001. Df = degrees of freedom (numerator, denominator), F = F-value, p = p-value.

Response		Df	F	p	R²
**Plants**					
1000 m scale	grassland amount	1, 51	1.23	**0.046** *	0.01
	habitat type	1, 51	14.72	**< 0.001** ***	0.16
	country	3, 51	7.60	**< 0.001** ***	0.17
	distance between paired habitats	1, 51	1.08	**< 0.001** ***	0.01
	grassland amount × habitat type	1, 51	1.24	0.392	0.01
	grassland amount × country	3, 51	1.08	**0.003** **	0.02
	habitat type × country	3, 51	6.40	**< 0.001** ***	0.14
2000 m scale	grassland amount	1, 51	1.74	**< 0.001** ***	0.02
	habitat type	1, 51	14.83	**< 0.001** ***	0.17
	country	3, 51	7.44	**< 0.001** ***	0.17
	distance between paired habitats	1, 51	1.08	**< 0.001** ***	0.01
	grassland amount × habitat type	1, 51	1.75	0.183	0.02
	grassland amount × country	3, 51	1.10	**< 0.001** ***	0.02
	habitat type × country	3, 51	6.19	**< 0.001** ***	0.14
**Butterflies**					
1000 m scale	grassland amount	1, 85	7.69	**< 0.001** ***	0.04
	habitat type	1, 85	23.77	**< 0.001** ***	0.12
	country	4, 85	17.87	**< 0.001** ***	0.36
	distance between paired habitats	1, 85	1.49	**< 0.001** ***	0.01
	grassland amount × habitat type	1, 85	1.14	0.453	0.01
	grassland amount × country	4, 85	1.24	**< 0.001** ***	0.02
	habitat type × country	4, 85	4.91	**< 0.001** ***	0.10
2000 m scale	grassland amount	1, 85	6.25	**< 0.001** ***	0.03
	habitat type	1, 85	24.84	**< 0.001** ***	0.12
	country	4, 85	17.88	**< 0.001** ***	0.36
	distance between paired habitats	1, 85	1.54	**< 0.001** ***	0.01
	grassland amount × habitat type	1, 85	0.64	0.831	< 0.01
	grassland amount × country	4, 85	1.70	**< 0.001** ***	0.03
	habitat type × country	4, 85	5.02	**< 0.001** ***	0.10
**Wild bees**					
1000 m scale	grassland amount	1, 85	3.01	**0.004** **	0.02
	habitat type	1, 85	1.21	**0.055 (*)**	0.01
	country	4, 85	11.04	0.510	0.35
	distance between paired habitats	1, 85	1.09	0.266	0.01
	grassland amount × habitat type	1, 85	0.96	0.444	0.01
	grassland amount × country	4, 85	1.06	0.642	0.03
	habitat type × country	4, 85	0.85	0.431	0.03
2000 m scale	grassland amount	1, 85	3.10	0.115	0.02
	habitat type	1, 85	1.21	**0.044** *	0.01
	country	4, 85	10.93	0.393	0.35
	distance between paired habitats	1, 85	1.10	0.234	0.01
	grassland amount × habitat type	1, 85	0.73	0.761	0.01
	grassland amount × country	4, 85	1.16	0.346	0.04
	habitat type × country	4, 85	0.80	0.501	0.03
**Carabids**					
1000 m scale	grassland amount	1, 71	2.06	**0.068 (*)**	0.02
	habitat type	1, 71	13.41	**< 0.001** ***	0.11
	country	4, 71	7.46	**< 0.001** ***	0.24
	distance between paired habitats	1, 71	0.96	**< 0.001** ***	0.01
	grassland amount × habitat type	1, 71	1.77	0.115	0.01
	grassland amount × country	4, 71	1.14	**< 0.001** ***	0.04
	habitat type × country	4, 71	4.13	**< 0.001** ***	0.13
2000 m scale	grassland amount	1, 71	3.23	**< 0.001** ***	0.03
	habitat type	1, 71	13.49	**< 0.001** ***	0.11
	country	4, 71	7.15	**< 0.001** ***	0.23
	distance between paired habitats	1, 71	0.99	**< 0.001** ***	0.01
	grassland amount × habitat type	1, 71	1.93	**0.057 (*)**	0.02
	grassland amount × country	4, 71	1.13	**< 0.001** ***	0.04
	habitat type × country	4, 71	4.27	**< 0.001** ***	0.14

Table S8: Type III Wald χ^2 tests for generalised linear mixed effects models of overall Bray Curtis dissimilarity as well as its turnover and nestedness components in relation to landscape permanent grassland cover at the 1000 m and 2000 m scales (separate models for the two scales), country, their interaction and the distance between the selected habitat patches across the four taxonomic groups vascular plants (n = 26), butterflies (n = 43), wild bees (n = 43), and carabids (n = 36). (*) indicates p < 0.10, * indicates p < 0.05, ** indicates p < 0.01, *** indicates p < 0.001. Df = degrees of freedom (numerator), χ^2 = chi-square value, p = p-value, [1] = indicates a zero-truncated model.

Response	residual distribution	Df	χ^2	p
**Plants**				
1000 m scale				
**overall**				
grassland amount	beta	1	0.29	0.587
country		2	5.85	**0.054 (*)**
distance between paired habitats		1	2.13	0.144
grassland amount × country		2	1.44	0.487
**turnover**				
grassland amount	beta	1	0.47	0.494
country		2	5.68	**0.058 (*)**
distance between paired habitats		1	1.99	0.158
grassland amount × country		2	1.64	0.440
**nestedness**				
grassland amount	beta	1	3.38	0.066 (*)
country		2	0.84	**0.657**
distance between paired habitats		1	1.45	0.228
grassland amount × country		2	0.13	0.937
2000 m scale				
**overall**				
grassland amount	beta	1	0.79	0.373
country		2	8.40	**0.015 ***
distance between paired habitats		1	2.12	0.145
grassland amount × country		2	2.16	0.339
**turnover**				
grassland amount	beta	1	0.67	0.414
country		2	6.93	**0.031 ***
distance between paired habitats		1	1.87	0.171
grassland amount × country		2	1.91	0.385
**nestedness**				
grassland amount	beta	1	4.38	**0.036 ***
country		2	1.10	0.576
distance between paired habitats		1	1.50	0.221
grassland amount × country		2	0.40	0.818
**Butterflies**				
1000 m scale				
**overall**				
grassland amount	beta	1	0.01	0.910
country		4	22.14	**< 0.001 *****
distance between paired habitats		1	1.06	0.304
grassland amount × country		4	3.60	0.463
**turnover**				
grassland amount	beta	1	0.08	0.784
country		4	5.26	0.262
distance between paired habitats		1	2.28	0.131
grassland amount × country		4	13.52	**0.009 ****
**nestedness**				
grassland amount	beta[1]	1	0.24	0.626
country		4	19.71	**< 0.001 *****
distance between paired habitats		1	0.60	0.438
grassland amount × country		4	9.61	**0.048 ***
2000 m scale				
**overall**				
grassland amount	beta	1	0.06	0.802
country		4	20.02	**< 0.001 *****
distance between paired habitats		1	1.23	0.268
grassland amount × country		4	7.56	0.109
**turnover**				
grassland amount	beta	1	0.05	0.818
country		4	8.65	**0.070 (*)**
distance between paired habitats		1	1.44	0.231

grassland amount × country		4	19.00	**< 0.001** ***
**nestedness**				
grassland amount		1	0.52	0.472
country	beta[1]	4	24.52	**< 0.001** ***
distance between paired habitats		1	0.23	0.632
grassland amount × country		4	9.42	**0.051 (*)**
**Wild bees**				
1000 m scale				
**overall**				
grassland amount		1	2.87	**0.090 (*)**
country	beta	4	1.57	0.814
distance between paired habitats		1	0.53	0.467
grassland amount × country		4	7.54	0.110
**turnover**				
grassland amount		1	2.13	**0.079 (*)**
country	beta[1]	4	1.48	0.625
distance between paired habitats		1	0.77	0.291
grassland amount × country		4	3.05	**0.024 ***
**nestedness**				
grassland amount		1	1.28	0.253
country	beta[1]	4	3.40	0.493
distance between paired habitats		1	0.07	0.798
grassland amount × country		4	7.53	0.111
2000 m scale				
**overall**				
grassland amount		1	3.51	**0.061 (*)**
country	beta	4	2.08	0.721
distance between paired habitats		1	0.16	0.690
grassland amount × country		4	10.41	**0.034 ***
**turnover**				
grassland amount		1	5.28	**0.022 ***
country	beta[1]	4	1.42	0.840
distance between paired habitats		1	0.64	0.423
grassland amount × country		4	11.85	**0.019 ***
**nestedness**				
grassland amount		1	6.11	**0.013 ***
country	beta[1]	4	7.34	0.119
distance between paired habitats		1	0.03	0.862
grassland amount × country		4	12.97	**0.011 ***
**Carabids**				
1000 m scale				
**overall**				
grassland amount		1	0.05	0.829
country	beta	4	35.44	**< 0.001** ***
distance between paired habitats		1	0.15	0.700
grassland amount × country		4	1.96	0.744
**turnover**				
grassland amount		1	8.62	**0.003** **
country	beta	4	28.19	**< 0.001** ***
distance between paired habitats		1	2.40	0.121
grassland amount × country		4	13.74	**0.008** **
**nestedness**				
grassland amount		1	3.92	**0.048 ***
country	beta	4	16.03	**0.003** **
distance between paired habitats		1	0.34	0.558
grassland amount × country		4	7.26	0.123
2000 m scale				
**overall**				
grassland amount		1	0.88	0.348
country	beta	4	25.71	**< 0.001** ***
distance between paired habitats		1	0.49	0.484
grassland amount × country		4	1.49	0.829
**turnover**				
grassland amount		1	7.70	**0.006** **
country	beta	4	25.32	**< 0.001** ***
distance between paired habitats		1	1.26	0.261
grassland amount × country		4	9.50	**0.049 ***
**nestedness**				
grassland amount	beta	1	2.43	0.119

country	4	11.44	**0.022 ***
distance between paired habitats	1	0.42	0.516
grassland amount × country	4	9.19	**0.056 (*)**

Table S9: Estimated marginal means with the range of the 95 % confidence interval for the generalised linear mixed effects models presented in Tables S5, S6, and S8 for each country. Countries: Bulgaria (BGR), Germany (DEU), Romania (ROM), Sweden (SWE) and Switzerland (CHE; country codes following ISO 3166). Values rounded to 1 decimal (richness & effective number of species) or two decimals (proportion of unique species & beta diversity measures). Different letters above the means indicate statistically significant differences ($p < 0.05$).

response / scale	plants			butterflies					wild bees					carabids				
	**DEU**<br>**n = 18**	**ROM**<br>**n = 14**	**SWE**<br>**n = 20**	**BGR**<br>**n = 16**	**DEU**<br>**n = 18**	**ROM**<br>**n = 16**	**SWE**<br>**n = 20**	**CHE**<br>**n = 16**	**BGR**<br>**n = 16**	**DEU**<br>**n = 18**	**ROM**<br>**n = 16**	**SWE**<br>**n = 20**	**CHE**<br>**n = 16**	**BGR**<br>**n = 16**	**DEU**<br>**n = 10**	**ROM**<br>**n = 14**	**SWE**<br>**n = 20**	**CHE**<br>**n = 12**
species richness																		
1000 m scale	a<br>17.5<br>[15.0; 20.5]	b<br>33.8<br>[30.0; 38.0]	c<br>11.6<br>[9.9; 13.6]	a<br>14.6<br>[11.4; 18.7]	a<br>11.7<br>[9.6; 14.3]	a<br>14.6<br>[12.4; 17.1]	b<br>5.7<br>[4.7; 7.0]	a<br>16.3<br>[13.6; 19.5]	ab<br>11.3<br>[8.2; 15.4]	a<br>11.4<br>[9; 14.3]	ab<br>9<br>[7.2; 11.3]	b<br>7.1<br>[5.8; 8.8]	ab<br>8.9<br>[6.9; 11.5]	a<br>31.1<br>[27.1; 35.7]	a<br>32.8<br>[28.8; 37.5]	b<br>19.2<br>[16.9; 21.9]	a<br>28.3<br>[26.2; 30.6]	c<br>12.8<br>[10.7; 15.3]
2000 m scale	a<br>18.6<br>[15.6; 22.2]	b<br>34.4<br>[30.4; 38.9]	c<br>11.7<br>[10.0; 13.7]	ab<br>15.8<br>[12.3; 20.4]	a<br>10.9<br>[8.8; 13.4]	b<br>16.3<br>[13.8; 19.1]	C<br>5.7<br>[4.7; 7.0]	b<br>16.2<br>[13.8; 19.0]	a<br>8.6<br>[5.9; 12.4]	a<br>10.9<br>[8.6; 13.9]	a<br>8.8<br>[6.9; 11.2]	a<br>7.2<br>[5.9; 8.8]	a<br>9.3<br>[7.4; 11.7]	a<br>36.8<br>[31.5; 42.9]	ab<br>33.3<br>[28.8; 38.5]	c<br>19.9<br>[17.3; 22.9]	b<br>28.3<br>[26.2; 30.5]	d<br>13.1<br>[10.9; 15.7]
eff. number of species																		
1000 m scale	a<br>12.7<br>[10.8; 15]	b<br>23.6<br>[20.9; 26.6]	a<br>9.9<br>[8.4; 11.6]	ab<br>6.2<br>[4.5; 8.5]	ab<br>6.1<br>[4.8; 7.7]	a<br>7.0<br>[5.8; 8.4]	b<br>4.4<br>[3.6; 5.4]	a<br>8.5<br>[7.0; 10.4]	a<br>8.5<br>[6.4; 11.3]	a<br>8.6<br>[7; 10.6]	a<br>7.1<br>[5.8; 8.7]	b<br>3.9<br>[3.1; 4.9]	a<br>6.5<br>[5.1; 8.2]	a<br>15.7<br>[12.9; 19.2]	b<br>8.0<br>[6.1; 10.5]	b<br>8.3<br>[6.8; 10.0]	b<br>9.6<br>[8.4; 11.0]	c<br>4.7<br>[3.5; 6.2]
2000 m scale	a<br>13.3<br>[11.0; 16.0]	b<br>23.8<br>[21.0; 27.0]	a<br>9.9<br>[8.4; 11.7]	ab<br>7.1<br>[5.1; 9.8]	ab<br>5.5<br>[4.2; 7.3]	a<br>7.4<br>[6.1; 9.1]	b<br>4.4<br>[3.6; 5.4]	a<br>8.4<br>[7.0; 10.1]	ab<br>6.6<br>[4.7; 9.3]	a<br>8.2<br>[6.6; 10.3]	a<br>7.1<br>[5.7; 8.8]	b<br>3.9<br>[3.1; 4.9]	a<br>6.8<br>[5.5; 8.4]	a<br>17.3<br>[13.8; 21.7]	bc<br>7.3<br>[5.3; 10.2]	b<br>8.3<br>[6.7; 10.2]	b<br>9.6<br>[8.4; 11.0]	c<br>4.7<br>[3.6; 6.3]
overall beta diversity																		
1000 m scale	a<br>0.94<br>[0.87; 0.97]	ab<br>0.97<br>[0.91; 0.99]	b<br>0.98<br>[0.96; 0.99]	a<br>0.49<br>[0.36; 0.62]	ab<br>0.65<br>[0.55; 0.74]	b<br>0.74<br>[0.65; 0.81]	b<br>0.71<br>[0.63; 0.78]	b<br>0.82<br>[0.73; 0.88]	a<br>0.68<br>[0.45; 0.84]	a<br>0.71<br>[0.55; 0.83]	a<br>0.74<br>[0.59; 0.85]	a<br>0.75<br>[0.63; 0.85]	a<br>0.63<br>[0.46; 0.77]	a<br>0.71<br>[0.59; 0.8]	ab<br>0.88<br>[0.8; 0.93]	bc<br>0.93<br>[0.88; 0.96]	a<br>0.76<br>[0.69; 0.82]	c<br>0.97<br>[0.94; 0.99]
2000 m scale	a<br>0.92<br>[0.84; 0.97]	ab<br>0.97<br>[0.92; 0.99]	b<br>0.98<br>[0.96; 0.99]	a<br>0.48<br>[0.33; 0.63]	ab<br>0.68<br>[0.57; 0.77]	b<br>0.73<br>[0.64; 0.81]	ab<br>0.71<br>[0.63; 0.77]	b<br>0.82<br>[0.74; 0.88]	a<br>0.60<br>[0.34; 0.81]	a<br>0.68<br>[0.5; 0.82]	a<br>0.72<br>[0.55; 0.84]	a<br>0.76<br>[0.64; 0.85]	a<br>0.63<br>[0.47; 0.76]	a<br>0.78<br>[0.65; 0.88]	ab<br>0.9<br>[0.79; 0.95]	b<br>0.93<br>[0.87; 0.96]	a<br>0.76<br>[0.69; 0.81]	b<br>0.97<br>[0.93; 0.99]
turnover component																		
1000 m scale	a<br>0.82<br>[0.65; 0.92]	ab<br>0.89<br>[0.75; 0.95]	b<br>0.95<br>[0.88; 0.98]	ab<br>0.42<br>[0.27; 0.57]	ab<br>0.53<br>[0.41; 0.64]	ab<br>0.52<br>[0.41; 0.63]	a<br>0.60<br>[0.50; 0.68]	b<br>0.39<br>[0.28; 0.50]	a<br>0.55<br>[0.30; 0.78]	a<br>0.56<br>[0.38; 0.73]	a<br>0.58<br>[0.41; 0.74]	a<br>0.48<br>[0.34; 0.62]	a<br>0.45<br>[0.29; 0.63]	ab<br>0.53<br>[0.38; 0.68]	a<br>0.66<br>[0.51; 0.79]	a<br>0.61<br>[0.49; 0.72]	b<br>0.30<br>[0.22; 0.40]	a<br>0.74<br>[0.61; 0.83]
2000 m scale	a<br>0.79<br>[0.58; 0.91]	ab<br>0.9<br>[0.77; 0.96]	b<br>0.95<br>[0.88; 0.98]	ab<br>0.39<br>[0.24; 0.57]	ab<br>0.56<br>[0.43; 0.68]	ab<br>0.46<br>[0.34; 0.58]	a<br>0.60<br>[0.50; 0.68]	b<br>0.36<br>[0.27; 0.47]	a<br>0.42<br>[0.14; 0.77]	a<br>0.51<br>[0.31; 0.71]	a<br>0.57<br>[0.39; 0.74]	a<br>0.48<br>[0.34; 0.63]	a<br>0.46<br>[0.30; 0.63]	ab<br>0.56<br>[0.37; 0.74]	a<br>0.67<br>[0.49; 0.81]	a<br>0.61<br>[0.47; 0.73]	b<br>0.30<br>[0.22; 0.40]	a<br>0.74<br>[0.61; 0.84]
nestedness component																		
1000 m scale	a<br>0.13<br>[0.07; 0.22]	a<br>0.18<br>[0.09; 0.34]	a<br>0.10<br>[0.04; 0.23]	a<br>0.09<br>[0.04; 0.19]	a<br>0.09<br>[0.04; 0.17]	a<br>0.19<br>[0.12; 0.29]	a<br>0.14<br>[0.08; 0.23]	b<br>0.44<br>[0.32; 0.56]	a<br>0.21<br>[0.10; 0.39]	a<br>0.18<br>[0.10; 0.31]	a<br>0.19<br>[0.10; 0.34]	a<br>0.28<br>[0.19; 0.39]	a<br>0.20<br>[0.11; 0.34]	a<br>0.15<br>[0.07; 0.32]	ab<br>0.20<br>[0.10; 0.38]	ab<br>0.32<br>[0.21; 0.47]	b<br>0.45<br>[0.34; 0.56]	ab<br>0.26<br>[0.16; 0.41]
2000 m scale	a<br>0.11<br>[0.06; 0.21]	a<br>0.19<br>[0.09; 0.35]	a<br>0.10<br>[0.04; 0.23]	a<br>0.09<br>[0.03; 0.23]	a<br>0.08<br>[0.03; 0.19]	ab<br>0.23<br>[0.14; 0.36]	a<br>0.14<br>[0.08; 0.23]	b<br>0.46<br>[0.35; 0.58]	a<br>0.26<br>[0.12; 0.47]	a<br>0.19<br>[0.11; 0.32]	a<br>0.12<br>[0.05; 0.25]	a<br>0.27<br>[0.19; 0.37]	a<br>0.18<br>[0.11; 0.30]	a<br>0.20<br>[0.08; 0.41]	a<br>0.22<br>[0.10; 0.41]	a<br>0.33<br>[0.20; 0.48]	a<br>0.45<br>[0.34; 0.56]	a<br>0.26<br>[0.15; 0.41]

Supplementary Note 2: Species driving habitat differences across countries

We identified 49 plant species associated with grasslands (12 species in Germany, 24 species in Romania and 21 species in Sweden) and 7 plant species associated with oilseed rape fields (one species in Germany, four species in Romania and three species in Sweden; Table S10). Plants associated with grassland habitats were mostly perennial (Fig. S20A) and either associated with nutrient poor conditions (e.g. *Teucrium chamaedrys*) or adapted to nutrient rich soils (e.g. *Plantago lanceolata*, *Veronica chamaedrys*) indicating a wide gradient in grassland characteristics across countries. The largest portion of the associated plant species across countries were grasses (14 of the 49 species, e.g. *Dactylis glomerata*, *Festuca pratensis*, *Poa angustifolia;* Table S10, Fig. S20A) and seven species belonged to the Fabaceae family (e.g. *Lotus corniculatus*, *Securigera varia*, *Trifolium repens*; Table S10, Fig. S20A). Only two species were associated with grasslands in all countries, the rather ubiquitous *Plantago lanceolata* and the grass *Dactylis glomerata*. Plants associated with oilseed rape fields mostly had annual life cycles (Fig. S20A) and were representants of classical arable weed communities like *Capsella bursa-pastoris*, *Chenopodium album*, *Stellaria media* and *Viola arvensis* or were indicators for nutrient rich soils (e.g. *Polygonum aviculare*, *Setaria pumila*).

Twenty-one butterfly species were associated with grasslands (two species in Bulgaria, three species in Germany, five species in Romania, four species in Sweden and 16 species in Switzerland; Table S11). Most of these species were overwintering as larvae and a considerable proportion only had one generation per year (Fig. S20B). Most butterfly species associated with grasslands reproduce on plant species typically found in grasslands with *Coenonympha pamphilus* (associated with the habitat in all countries) and *Maniola jurtina* (associated in Germany, Sweden and Switzerland), both reproducing on grasses, being the species most commonly associated with grasslands. Eight of the 21 species belonged to the family Lycaenidae, many of which are reproducing on Fabaceae commonly found in and also associated with grasslands (*Cupido argiades*, *Lysandra bellargus*, *Polyommatus icarus*, *Polyommatus thersites*). In oilseed rape fields, we found six associated butterfly species (three species in Bulgaria, four species in Germany and two species in each of the remaining countries; Table S11). These species were all multivoltine and four of the six species overwintered exclusively or partly as imagines, with species overwintering as eggs or larvae being absent (Fig. S20B). These species were either directly reproducing on oilseed rape (*Pieris rapae*, associated in all countries except Sweden) or other Brassicaceae (*Pieris napi*, associated in Sweden and Switzerland) or were highly mobile ubiquitists that reproduce on *Urtica dioica*

(*Aglais io*, *Aglais urticae*) or also on other common arable weeds (*Vanessa cardui* reproducing on a wide range of host plants from different families and *Issoria lathonia* reproducing among others on *Viola arvensis* that was found associated to oilseed rape fields as well).

We found no wild bee species associated with grasslands, but ten species were associated with oilseed rape fields (five species in Bulgaria, one species in Germany, four species in Romania and one species in Switzerland), albeit only *Lasioglossum pauxillum* in more than one country (Bulgaria and Romania; Table S12). All these species were ground nesting (Fig. S20C), and it seems plausible that they might have utilised open field margins or bare soil along farm roads for nesting. Five of the species belonged to the genus *Lasioglossum*, three to the genus *Andrena*, one to the genus *Anthophora* and the remaining species, all of them being polylactic generalists, the remaining *Sphecodes crassus*, is a nest parasite of *Lasioglossum* (Fig. S20C). As we observed foraging wild bees and not their nests (or reproductive success), we cannot draw any conclusions about the suitability of oilseed rape fields for the reproduction of these species. We believe that these species are likely not nesting in the oilseed rape fields but might use their edges (see Tschanz, et al. [6]) or the closer vicinity of the fields for nesting and reproduction.

In carabids, we found twelve species associated with grasslands (five species in Bulgaria, three species each in Germany and Sweden, two species in Switzerland), albeit only the rather ubiquitist *Poecilus versicolor* in more than one country (Germany and Sweden), and 40 species associated with oilseed rape fields (17 species in Bulgaria, 20 species in Germany, twelve species in Romania, 15 species in Sweden and 16 species in Switzerland; Table S13). Grassland-associated carabids were predominantly granivorous (Fig. S20D), with nine of the twelve species being predominantly granivorous (*Amara communis*, *Amara lunicollis*, *Harpalus albanicus, Harpalus attenuatus*, *Harpalus dimidiatus*, *Harpalus flavicornis*, *Harpalus rubripes*, *Ophonus azureus*) or facultatively granivorous (*Poecilus versicolor*). These species likely benefitted from the high availability of seed resources, especially from perennial grasses, in grasslands. Of the remaining species, *Microlestes maurus* and *Microlestes minutulus*, are xerophilous species benefitting from the more open and exposed habitat structure of grasslands. In contrast, the carabid species associated with oilseed rape fields were predominantly predatory (Fig. S20D) and mostly comprised classical species characteristic of agricultural habitats across Europe [5] with four species, *Amara similata*, *Anchomenus dorsalis*, *Harpalus rufipes* and *Trechus qudristriatus* associated with the habitat in all five countries and two species, *Harpalus affinis* and *Poecilus cupreus*, associated with oilseed rape in all but one country. Most of the species associated with oilseed rape fields are adapted to frequent disturbances and naturally occur in early succession habitats like dynamic river shores or

mountain landslides and have become common in agricultural habitats defined by frequent soil disturbances (see data from crop fields presented in Boetzl et al. 2024). Interestingly, there was no clear difference in the distribution of overwintering strategies between the habitats (Fig. S20D) which could indicate that many carabid imagines can successfully overwinter in frequently disturbed crop fields [7-9]. *Amara similata*, the species most commonly associated with oilseed rape across countries, is likely also a facultative pest on oilseed rape and often encountered in large numbers in the crop [10]. Some of the species associated with oilseed rape also inhabit the system of soil crevasses that are often found in arable fields with regular soil disturbance (*Clivina fossor*, *Tachys scutellaris*, *Zuphium olens*).

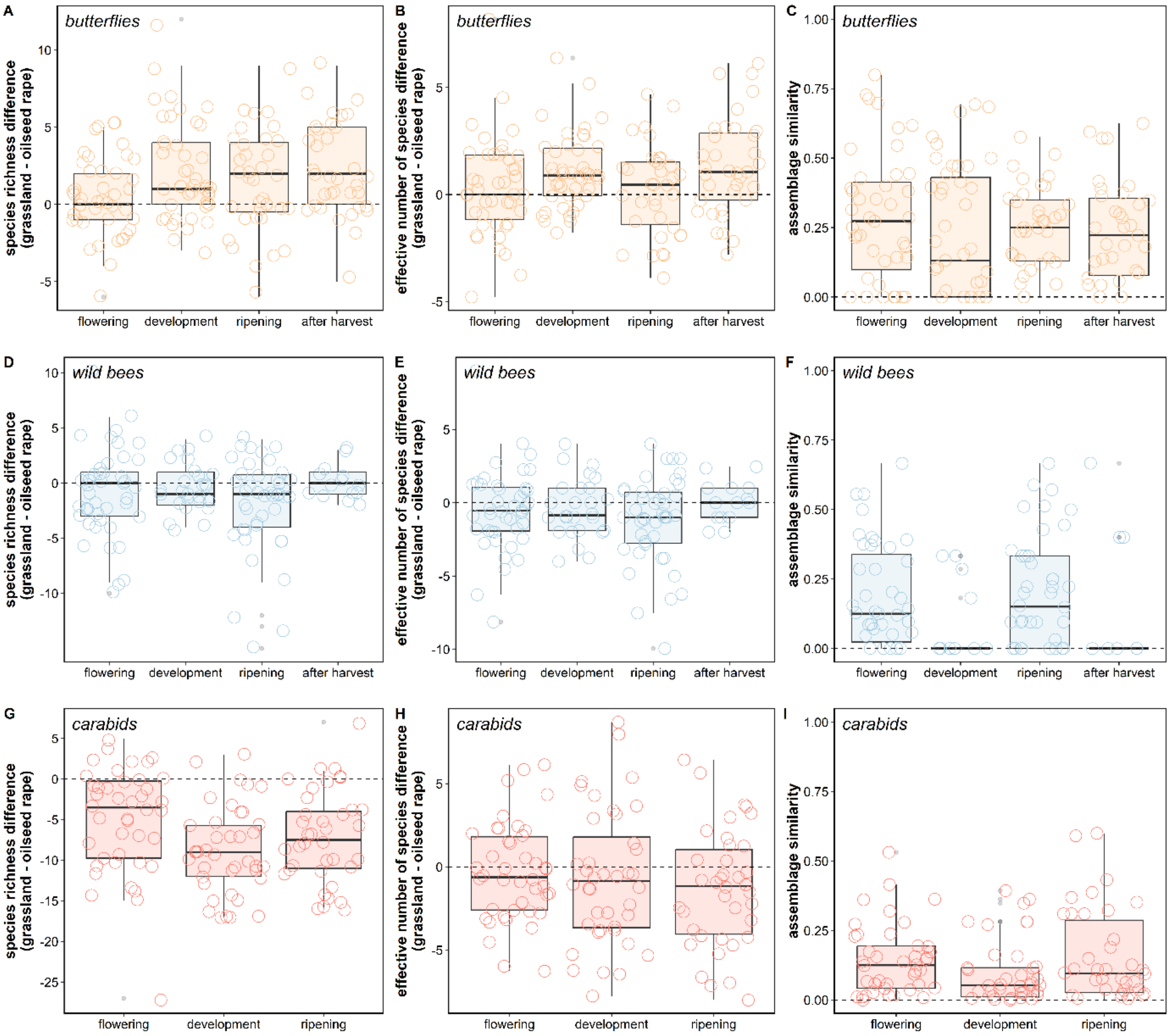

Fig. S17: Difference in species richness (A, D, G) and effective number of species (B, E, H) as well as species assemblage similarity (C, F, I) between the two paired habitats (grassland – oilseed rape) across oilseed rape phenology and all countries in butterflies (A, B, C; n = 43), wild bees (D, E, F; n = 43) and carabids (G, H, I; n = 36). Dashed lines in A, B, D, E, G and H indicate no difference in species richness between the paired habitats.

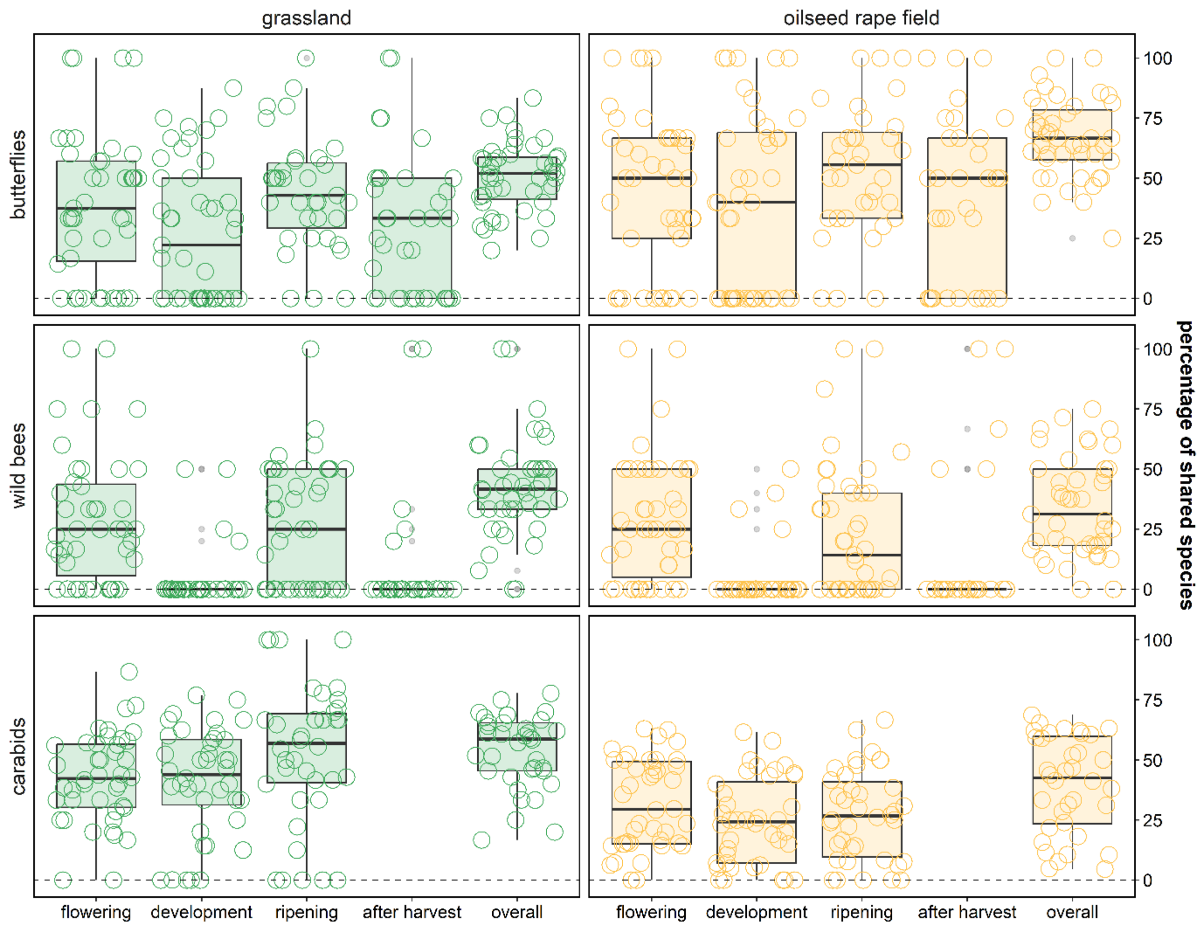

Fig. S18: Percentage of species shared between the paired permanent grasslands (left) and oilseed rape fields (right) of the species assemblages of butterflies (top; n = 43), wild bees (middle; n = 43) and carabids (bottom; n = 36) in the respective habitat type across the different sampling intervals along oilseed rape phenology and across the whole sampling period ('overall').

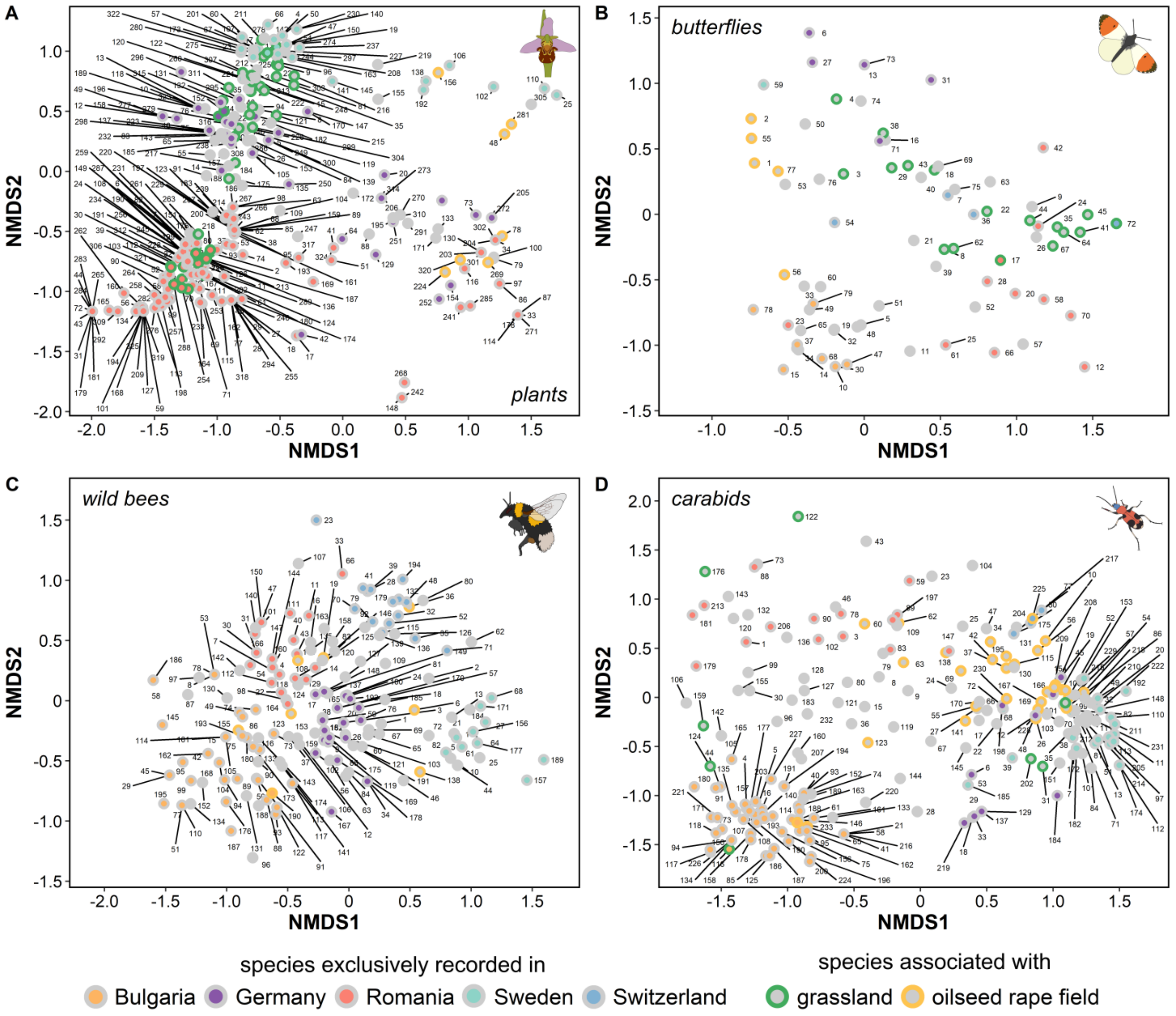

Fig. S19: Species assemblages of plants (A), butterflies (B), wild bees (C) and carabids (D; NMDS ordinations) in the two sampled habitat types (oilseed rape and grassland) across five European countries (Bulgaria, Germany, Romania, Sweden and Switzerland). Points indicate the individual species in the assemblages; numbers refer to the rows in the species lists provided in Tables S10 to S13. Coloured cores indicate that the respective species was exclusively recorded in the respective country and likely to drive assemblage differences between countries, coloured outer rings indicate that the respective species was determined as habitat-associated species that is likely to drive assemblage differences between habitats (Supplementary note 3, Tables S10 to S13). The NMDS used Bray-Curtis distances on proportional abundances or proportions of cover (plants). For statistics, see text and Table S7.

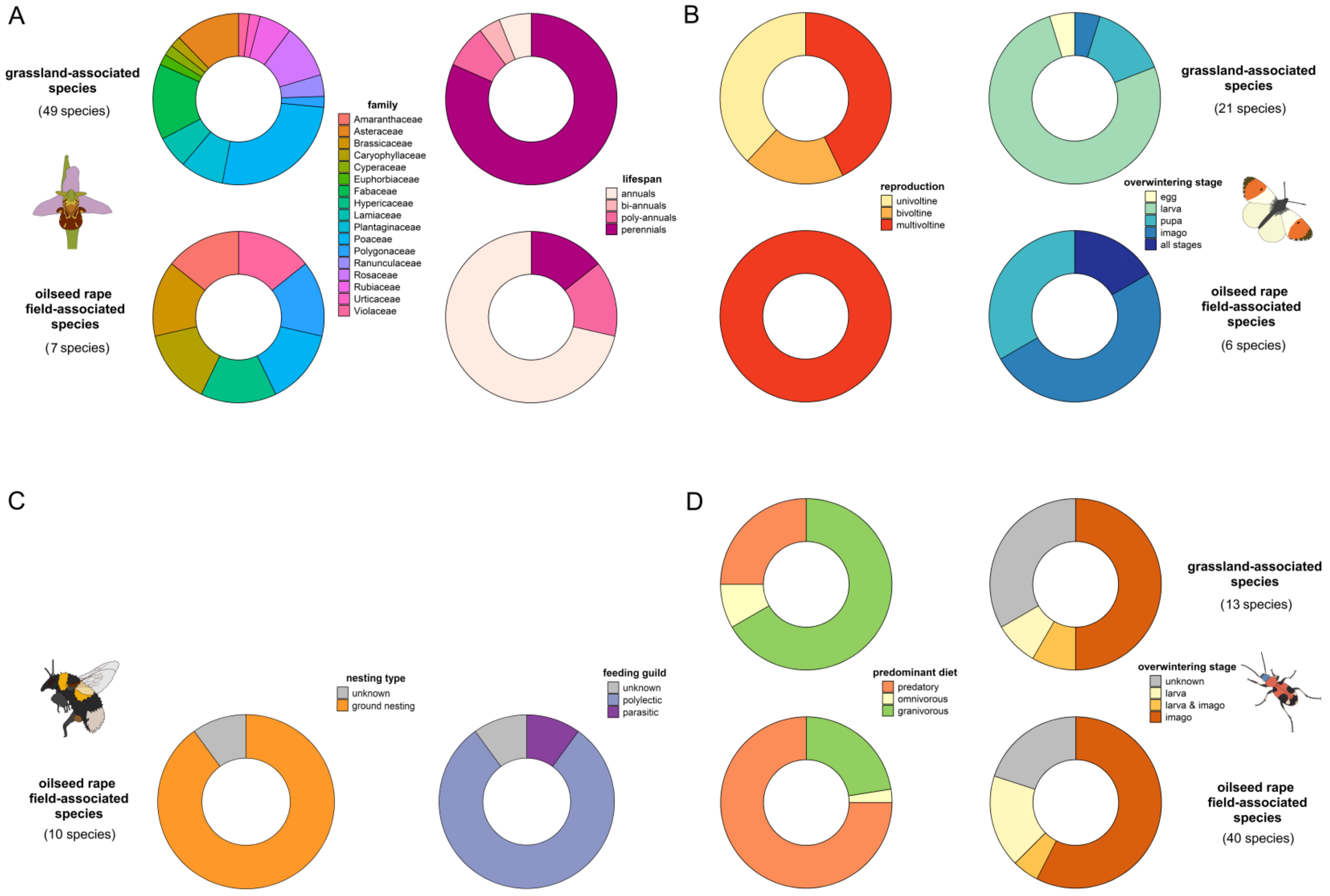

Fig. S20: Distribution of selected traits among species identified as associated with one of the two habitat types. Donut charts indicate the proportional representation of the respective traits among the habitat-associated species in both habitat types. Traits include family and lifespan in plants (A; taken from Kleyer, et al. [1]), reproduction strategy and overwintering stage in butterflies (B; taken from Middleton-Welling, et al. [2]), nesting type and feeding guild in wild bees (C; taken from Westrich [3]) and predominant diet and overwintering stage in carabids (D; taken from Lindroth [4] and Boetzl, et al. [5]).

Table S10: Plant species recorded in grasslands (GR) and oilseed rape fields (OSR) across four countries (Germany, Romania, and Sweden). Plant species presence is indicated by ‘x’. Green and yellow highlights indicate species found predominantly in the grasslands or oilseed rape fields, respectively, in the respective country (significant two-sided paired t-test; $p < 0.05$) and are thus influential for differences between habitats.

	**species**	**Germany**		**Romania**		**Sweden**	
		**GR n = 9**	**OSR n = 9**	**GR n = 7**	**OSR n = 7**	**GR n = 10**	**OSR n = 10**
1	*Acer platanoides*	x					
2	*Achillea collina*			x	x		
3	*Achillea millefolium*	x				x	
4	*Achillea ptarmica*					x	
5	*Achillea setacea*	x					
6	*Adonis vernalis*			x			
7	*Agrimonia eupatoria*	x		x			
8	*Agrostis canina*			x		x	
9	*Agrostis capillaris*	x				x	
10	*Agrostis gigantea*	x					
11	*Ajuga genevensis*			x			
12	*Alchemilla vulgaris*	x					
13	*Alopecurus aequalis*	x				x	
14	*Alopecurus myosuroides*	x	x				
15	*Alopecurus pratensis*	x				x	
16	*Amaranthus albus*	x	x				
17	*Anagallis arvensis*			x	x		
18	*Anchusa barrelieri*			x			
19	*Anemone nemorosa*					x	
20	*Anthemis arvensis*	x	x				
21	*Anthoxanthum odoratum*	x		x		x	
22	*Anthriscus caucalis*	x					
23	*Anthriscus sylvestris*					x	
24	*Anthyllis vulneraria*			x			
25	*Apera spica venti*						x
26	*Arrhenatherum elatius*	x	x	x			
27	*Artemisia absinthium*			x			
28	*Artemisia campestris*			x			
29	*Asparagus tenuifolius*			x			
30	*Asperula cynanchica*			x			
31	*Aster linosyris*			x			
32	*Astragalus monspessulanus*			x			
33	*Avena sativa*				x		
34	*Ballota nigra*				x		
35	*Bellis perennis*	x				x	
36	*Bothriochloa ischaemum*			x			
37	*Brachypodium pinnatum*			x			
38	*Briza media*			x			
39	*Bromus erectus*			x			
40	*Bromus hordeaceus*	x	x			x	
41	*Bromus squarrosus*			x	x		
42	*Bromus sterilis*		x				
43	*Buglossoides purpurocaerulea*			x			
44	*Bupleurum falcatum*			x			
45	*Camelina microcarpa*			x			
46	*Campanula patula*	x					
47	*Campanula rotundifolia*					x	
48	*Capsella bursa-pastoris*		x		x	x	x
49	*Cardamine amara*	x					
50	*Cardamine pratensis*					x	
51	*Cardaria draba*			x	x		
52	*Carduus collinus*			x			
53	*Carduus hamulosus*			x			
54	*Carex ericetorum*					x	
55	*Carex hirta*	x		x			

56	*Carex humilis*			x			
57	*Carex leporina*					x	
58	*Carex michelii*			x			
59	*Carex montana*			x			
60	*Carex panicea*					x	
61	*Carex tomentosa*			x			
62	*Carlina vulgaris*			x			
63	*Caucalis platycarpos*			x			
64	*Centaurea cyanus*	x	x				
65	*Centaurea jacea*	x		x			
66	*Centaurea montana*					x	
67	*Centaurea nigra*					x	
68	*Centaurea nigrescens*			x			
69	*Centaurea scabiosa*			x	x		
70	*Centaurea stoebe*			x			
71	*Cephalaria radiata*			x			
72	*Cephalaria uralensis*			x			
73	*Cerastium arvense*		x				
74	*Cerastium brachypetalum*			x	x		
75	*Cerastium glomeratum*	x					
76	*Cerastium holosteoides*	x					
77	*Cerinthe minor*			x			
78	*Chenopodium album*		x		x		
79	*Chenopodium hybridum*				x		
80	*Cichorium intybus*			x			
81	*Cirsium arvense*	x	x	x	x	x	x
82	*Cirsium eriophorum*			x			
83	*Cirsium oleraceum*	x					
84	*Cirsium vulgare*	x		x	x	x	
85	*Clinopodium vulgare*			x			
86	*Conium maculatum*				x		
87	*Consolida regalis*				x		
88	*Convolvulus arvensis*	x	x	x	x	x	
89	*Cornus sanguinea*			x			
90	*Crataegus monogyna*			x			
91	*Crepis biennis*	x		x			
92	*Cruciata glabra*			x			
93	*Cynodon dactylon*			x	x		
94	*Dactylis glomerata*	x	x	x		x	
95	*Daucus carota*	x		x	x		
96	*Deschampsia cespitosa*	x				x	
97	*Descurainia sophia*				x		
98	*Dianthus carthusianorum*			x			
99	*Dorycnium herbaceum*			x			
100	*Echinochloa crus galli*		x		x		
101	*Echium vulgare*			x			
102	*Elymus repens*					x	x
103	*Elytrigia intermedia*			x			
104	*Elytrigia repens*	x	x	x	x		
105	*Epilobium ciliatum*	x					
106	*Equisetum arvense*					x	x
107	*Equisetum palustre*					x	
108	*Erigeron acris*			x			
109	*Erigeron annuus*			x			
110	*Erodium cicutarium*						x
111	*Eryngium campestre*			x			
112	*Euphorbia cyparissias*			x	x		
113	*Euphorbia esula*			x			
114	*Euphorbia peplus*				x		
115	*Falcaria vulgaris*			x			
116	*Fallopia convolvulus*				x		
117	*Festuca arenaria*					x	

118	*Festuca brevipila*	x					
119	*Festuca guestfalica*	x					
120	*Festuca ovina*					x	
121	*Festuca pratensis*	x		x	x	x	x
122	*Festuca rubra*	x		x		x	
123	*Festuca rupicola*	x		x			
124	*Festuca valesiaca*			x	x		
125	*Filipendula ulmaria*					x	
126	*Filipendula vulgaris*			x			
127	*Fragaria vesca*			x			
128	*Fragaria viridis*			x			
129	*Fumaria officinalis*		x				
130	*Galeopsis bifida*				x		
131	*Galeopsis tetrahit*	x	x			x	
132	*Galium album*	x					
133	*Galium aparine*		x		x		
134	*Galium glaucum*			x			
135	*Galium mollugo*	x		x			
136	*Galium verum*	x		x			
137	*Geranium pratense*	x					
138	*Geranium robertianum*					x	x
139	*Geranium rotundifolium*	x					
140	*Geranium sanguineum*					x	
141	*Geranium sylvaticum*					x	
142	*Geum rivale*					x	
143	*Geum urbanum*	x					
144	*Glechoma hederacea*	x		x		x	
145	*Glyceria fluitans*					x	
146	*Helictotrichon pubescens*	x		x		x	
147	*Heracleum sphondylium*	x					
148	*Hibiscus trionum*				x		
149	*Hieracium bauhini*			x			
150	*Hieracium peleteranium*					x	
151	*Hieracium pilosella*	x		x			
152	*Holcus lanatus*	x					
153	*Hordelymus europaeus*	x					
154	*Hordeum vulgare*		x				
155	*Hypericum maculatum*			x		x	x
156	*Hypericum perforatum*	x				x	x
157	*Hypochaeris radicata*	x		x			
158	*Impatiens glandulifera*	x					
159	*Inula bifrons*			x			
160	*Inula ensifolia*			x			
161	*Inula salicina*			x			
162	*Iris pontica*			x			
163	*Juncus effusus*					x	
164	*Jurinea mollis*			x			
165	*Kengia serrotina*			x			
166	*Knautia arvensis*			x			
167	*Koeleria macrantha*			x			
168	*Lactuca saligna*			x			
169	*Lactuca serriola*			x	x		
170	*Lamium album*	x					
171	*Lamium purpureum*		x		x		
172	*Lapsana communis*	x			x		
173	*Lathyrus pratensis*	x				x	
174	*Lathyrus tuberosus*			x	x		
175	*Leontodon autumnalis*	x		x			
176	*Leontodon hispidus*	x		x			
177	*Leucanthemum vulgare*			x			
178	*Linaria vulgaris*				x		
179	*Linum austriacum*			x			

180	*Linum catharticum*			x			
181	*Linum flavum*			x			
182	*Lolium multiflorum*	x	x			x	
183	*Lolium perenne*	x	x	x			
184	*Lotus corniculatus*	x		x		x	
185	*Luzula campestris*	x		x			
186	*Lysimachia nummularia*			x			
187	*Medicago falcata*			x			
188	*Medicago lupulina*			x		x	
189	*Medicago x varia*	x					
190	*Melica transsilvanica*			x			
191	*Melilotus officinalis*			x			
192	*Mentha arvensis*					x	x
193	*Mentha longifolia*			x	x		
194	*Muscari comosum*			x			
195	*Myosotis arvensis*	x	x		x		
196	*Myosotis scorpioides*	x					
197	*Nepeta cataria*			x			
198	*Nonea pulla*			x			
199	*Onobrychis viciifolia*			x			
200	*Ononis arvensis*			x			
201	*Origanum vulgare*					x	
202	*Ornithogalum umbellatum*			x			
203	*Oxalis fontana*				x		
204	*Panicum miliaceum*				x		
205	*Persicaria lapathifolia*		x				
206	*Persicaria maculosa*		x				
207	*Peucedanum carvifolia*			x			
208	*Peucedanum palustre*					x	
209	*Peucedanum tauricum*			x			
210	*Phalaris arundinacea*	x					
211	*Phaseolus vulgaris*					x	
212	*Phleum pratense*	x				x	
213	*Picris hieracioides*			x			
214	*Pimpinella saxifraga*			x			
215	*Plantago lanceolata*	x	x	x	x	x	
216	*Plantago major*	x	x			x	
217	*Plantago media*			x	x	x	
218	*Poa angustifolia*			x			
219	*Poa annua*		x			x	x
220	*Poa compressa*			x			
221	*Poa nemoralis*					x	
222	*Poa pratensis*	x		x	x	x	x
223	*Poa trivialis*	x					
224	*Polygonum aviculare*	x	x	x	x		
225	*Potentilla anserina*	x				x	
226	*Potentilla argentea*			x		x	
227	*Potentilla erecta*			x		x	
228	*Potentilla incana*			x			
229	*Potentilla recta*			x			
230	*Potentilla reptans*					x	
231	*Prunella vulgaris*			x			
232	*Prunus avium*	x					
233	*Prunus spinosa*			x			
234	*Pyrus pyraster*			x			
235	*Quercus robur*	x				x	
236	*Ranunculus acris*	x		x	x	x	
237	*Ranunculus bulbosus*					x	
238	*Ranunculus ficaria*	x					
239	*Ranunculus polyanthemos*			x			
240	*Ranunculus repens*	x	x	x			
241	*Rapistrum perenne*				x		

242	*Reseda lutea*				x		
243	*Rhinanthus angustifolius*			x			
244	*Rhinanthus minor*					x	
245	*Rosa canina*			x			
246	*Rosa gallica*			x			
247	*Rubus caesius*	x		x	x		
248	*Rumex acetosa*	x	x	x		x	
249	*Rumex acetosella*	x					
250	*Rumex crispus*	x	x				
251	*Rumex obtusifolius*		x				
252	*Rumex x pratensis*		x				
253	*Salvia austriaca*			x			
254	*Salvia nemorosa*			x			
255	*Salvia nutans*			x			
256	*Salvia pratensis*			x			
257	*Salvia verticillata*			x	x		
258	*Sanguisorba minor*			x			
259	*Sanguisorba officinalis*			x			
260	*Saxifraga granulata*	x				x	
261	*Scabiosa ochroleuca*			x			
262	*Scorzonera hispanica*			x			
263	*Securigera varia*	x		x			
264	*Senecio jacobaea*			x			
265	*Serratula lycopifolia*			x			
266	*Seseli annuum*			x			
267	*Seseli pallasii*			x			
268	*Setaria glauca*				x		
269	*Setaria pumila*		x		x		
270	*Silene latifolia*	x	x	x	x		
271	*Sinapis alba*				x		
272	*Sisymbrium officinale*		x				
273	*Sonchus arvensis*		x				
274	*Sorbus aucuparia*					x	
275	*Stachys palustris*					x	
276	*Stachys recta*			x			
277	*Stellaria alsine*	x					
278	*Stellaria graminea*			x		x	
279	*Stellaria holostea*	x					
280	*Stellaria longifolia*					x	
281	*Stellaria media*	x	x	x	x		x
282	*Stipa capillata*			x			
283	*Stipa lessingiana*			x			
284	*Stipa pulcherrima*			x			
285	*Symphytum officinale*				x		
286	*Taraxacum sect Ruderalia*	x	x	x	x		
287	*Tetragonolobus maritimus*			x			
288	*Teucrium chamaedrys*			x			
289	*Thalictrum minus*			x			
290	*Thesium linophyllon*			x			
291	*Thlaspi arvense*		x		x		
292	*Thymelaea passerina*			x			
293	*Thymus pannonicus*			x			
294	*Torilis japonica*			x			
295	*Trifolium campestre*	x					
296	*Trifolium dubium*	x					
297	*Trifolium medium*					x	
298	*Trifolium pratense*	x		x			
299	*Trifolium repens*	x	x	x		x	
300	*Trifolium striatum*	x					
301	*Tripleurospermum perforatum*				x		
302	*Triticum aestivum*				x		
303	*Urtica dioica*	x	x			x	

304	*Valerianella locusta*	x					
305	*Veronica arvensis*	x	x	x	x		x
306	*Veronica austriaca*			x			
307	*Veronica chamaedrys*	x		x		x	
308	*Veronica hederifolia*	x					
309	*Veronica orchidea*			x			
310	*Veronica persica*	x	x		x		
311	*Veronica serpyllifolia*	x				x	
312	*Vicia angustifolia*			x			
313	*Vicia cracca*	x		x		x	
314	*Vicia hirsuta*		x				
315	*Vicia sativa*	x					
316	*Vicia sepium*	x					
317	*Vicia tenuifolia*			x	x		
318	*Vinca herbacea*			x			
319	*Viola ambigua*			x			
320	*Viola arvensis*		x		x		
321	*Viola hirta*			x			
322	*Viola riviniana*					x	
323	*Vulpia myuros*	x					
324	*Xanthium strumarium*			x	x		
325	*Xeranthemum annuum*			x			
	**Total**	107	47	182	62	80	15
	**Shared species**	27		31		11	

Table S11: Butterfly species recorded in grasslands (GR) and oilseed rape fields (OSR) across all countries (Bulgaria, Germany, Romania, Sweden and Switzerland) with total abundances. Green and yellow highlights indicate species found predominantly in the grasslands or oilseed rape fields, respectively, in the respective country (significant two-sided paired t-test; $p < 0.05$) and are thus influential for differences between habitats.

	species	Bulgaria		Germany		Romania		Sweden		Switzerland	
		GR n = 8	OSR n = 8	GR n = 9	OSR n = 9	GR n = 8	OSR n = 8	GR n = 10	OSR n = 10	GR n = 8	OSR n = 8
1	*Aglais io*	1	7	30	132		8	7	25	3	7
2	*Aglais urticae*		2		8			3	15		1
3	*Anthocharis cardamines*	6	1	8	6		1	4	1	12	3
4	*Aphantopus hyperantus*			86	20	3	2	81	7		2
5	*Aporia crataegi*	35	3	1		4	1	1	1		
6	*Araschnia levana*			19							
7	*Argynnis paphia*									10	2
8	*Aricia agestis*	8	5	4	6	2	3			74	14
9	*Boloria dia*					11	7			175	20
10	*Boloria euphrosyne*	1									
11	*Brenthis daphne*	3				1					
12	*Brenthis hecate*					2					
13	*Brenthis ino*			1							
14	*Callophrys rubi*	1	1			1	1				
15	*Carcharodus alceae*	1	1								
16	*Celastrina argiolus*			2	1						2
17	*Coenonympha glycerion*					63	20				
18	*Coenonympha pamphilus*	70	13	173	25	78	18	26		238	47
19	*Colias croceus*	36	13			9	10			2	1
20	*Colias erate*					3					
21	*Colias hyale / alfacariensis*			3	2	9	6			17	7
22	*Cupido argiades*		2	2			2			27	4
23	*Cupido minimus*						1				
24	*Cupido osiris*					4					
25	*Cyaniris semiargus*	1					1	1		47	7
26	*Erynnis tages*					9	2			30	1
27	*Favonius quercus*			5	2						
28	*Glaucopsyche alexis*					10	4				
29	*Gonepteryx rhamni*	2		11	9					14	3
30	*Hamearis lucina*	2									
31	*Heteropterus morpheus*			1							
32	*Iphiclides podalirius*	3				2	9				
33	*Issoria lathonia*	36	46	8	35	1	5				
34	*Lasiommata megera*	3	3							1	
35	*Leptidea sinapis / juvernica*	1				7	1			64	7
36	*Limenitis camilla*									3	1
37	*Lycaena dispar*	4	4								
38	*Lycaena phlaeas*	8	3	22	3			9		5	2
39	*Lycaena thersamon*	1				1	1				
40	*Lycaena tityrus*	4		25	3					15	1
41	*Lysandra bellargus*					11				52	2
42	*Maniola jurtina*	58	20	145	42	87	49	77	11	421	83
43	*Melanargia galathea*			21	2	42	33			655	28
44	*Melitaea athalia*					6	1			774	37
45	*Melitaea aurelia*					5					
46	*Melitaea cinxia*	6									
47	*Melitaea didyma*	12	5							8	1
48	*Melitaea phoebe*	12	6				1				
49	*Ochlodes sylvanus*		1	21	3			2	2	4	1
50	*Papilio machaon*	4	2	1		3	3			1	
51	*Pararge aegeria*						1			1	
52	*Phengaris arion*					2					
53	*Pieris brassicae*	7	13	29	25	3	6	4	7	5	7
54	*Pieris mannii*									1	1
55	*Pieris napi*	2	1	167	180		2	15	86	8	30

56	*Pieris rapae*	232	452	49	184	19	216	39	47	5	69
57	*Plebejus argus*	19	2		1	444	59	1			
58	*Plebejus argyrognomon*					13	2				
59	*Plebejus idas*							1			
60	*Polygonia c-album*	10	3	2	2						1
61	*Polyommatus daphnis*						1				
62	*Polyommatus icarus*	47	4	15	2	45	33			250	63
63	*Polyommatus thersites*					11	2			90	5
64	*Pontia edusa*	20	30	1	5	3	16				
65	*Pyrgus carthami*					2	3				
66	*Pyrgus malvae*					6					1
67	*Satyrium ilicis*	13	3								
68	*Satyrium pruni*			1		1					
69	*Satyrium spini*					1					
70	*Satyrium w-album*			1							
71	*Spialia sertorius*									13	
72	*Thecla betulae*			1							
73	*Thymelicus lineola*			14	5		2	2		3	
74	*Thymelicus sylvestris*	1		3	1	1				2	
75	*Vanessa atalanta*	4	1	7	7	1		1			1
76	*Vanessa cardui*	3	9	2	2		1	1	6		1
77	*Zerynthia cerisy*		3								
78	*Zerynthia polyxena*	17	7								
**Total abundance**		694	666	881	713	926	534	275	208	3030	463
**Total richness**		38	31	34	27	39	38	18	11	34	35
**Shared species**		27		25		27		11		28	

Table S12: Wild bee species recorded in grasslands (GR) and oilseed rape fields (OSR) across all countries (Bulgaria, Germany, Romania, Sweden and Switzerland) with total abundances. Green and yellow highlights indicate species found predominantly in the grasslands or oilseed rape fields, respectively, in the respective country (significant two-sided paired t-test; $p < 0.05$) and are thus influential for differences between habitats.

	species	Bulgaria		Germany		Romania		Sweden		Switzerland	
		GR n = 8	OSR n = 8	GR n = 9	OSR n = 9	GR n = 8	OSR n = 8	GR n = 10	OSR n = 10	GR n = 8	OSR n = 8
1	*Andrena alfkenella*						4	2	4		
2	*Andrena apicata*								2		1
3	*Andrena argentata*			7							
4	*Andrena atrata*						1				
5	*Andrena barbilabris*							1			
6	*Andrena bicolor*			6	6		4	14	27	1	
7	*Andrena bisulcata*						1				
8	*Andrena braunsiana*	1									
9	*Andrena bucephala*				2						
10	*Andrena carantonica*						1	1	3		
11	*Andrena chrysopyga*					2	3				
12	*Andrena chrysosceles*			5	3						1
13	*Andrena cineraria*							23	12		3
14	*Andrena coitana*					1	1				
15	*Andrena colletiformis*		1								
16	*Andrena combinata*		1			1					1
17	*Andrena curvungula*			3	1						
18	*Andrena dorsata*				2		2	1			
19	*Andrena flavipes*	2	6	4	7	9	38	2	2	5	8
20	*Andrena floricola*				2						
21	*Andrena fulva*			1				1	4		1
22	*Andrena fulvago*					1					
23	*Andrena fulvida*									1	
24	*Andrena gravida*			5	2					3	
25	*Andrena haemorrhoa*			2	1			382	290		4
26	*Andrena hattorfiana*		1		1						
27	*Andrena helvola*							15	8		
28	*Andrena humilis*									3	
29	*Andrena impunctata*	2									
30	*Andrena intermedia*						1				
31	*Andrena labialis*						1				
32	*Andrena lagopus*										1
33	*Andrena marginata*					1					
34	*Andrena minutula*				1						
35	*Andrena morio*									1	2
36	*Andrena nitida*			4						2	10
37	*Andrena nitidiscula*		3				1				
38	*Andrena niveata*			1							
39	*Andrena ovatula*									4	6
40	*Andrena pandellei*						3				
41	*Andrena pandelli*									2	1
42	*Andrena panurgimorpha*	2									
43	*Andrena paucisquama*					1	4				
44	*Andrena semilaevis*			1				1	5		
45	*Andrena simillima*	1									
46	*Andrena subopaca*	1	3	5	7						
47	*Andrena symphyti*						2				
48	*Andrena taraxaci*									2	
49	*Andrena tarsata*	1									
50	*Andrena tenuis*		3								
51	*Andrena transitoria*	1									
52	*Andrena vaga*										1
53	*Andrena ventricosa*	1									
54	*Anthidium punctatum*					1					
55	*Anthophora crassipes*	1									

56	*Anthophora plumipes*				5		1				
57	*Anthophora retusa*				1						
58	*Bombus argillaceus*	1	1								
59	*Bombus barbutellus*			1							
60	*Bombus hortorum*			1	2			2			
61	*Bombus hypnorum*							1			
62	*Bombus lapidarius*							3		1	1
63	*Bombus lucorum*		1		2						1
64	*Bombus muscorum*								1		
65	*Bombus pascuorum*			3	6	1		1	1	2	
66	*Bombus pomorum*					1					
67	*Bombus pratorum*			3	1				1		1
68	*Bombus ruderarius*							10	4		
69	*Bombus soroeensis*		1	3	3				1		
70	*Bombus sylvarum*										1
71	*Bombus sylvestris*										2
72	*Bombus terrestris*			1	2		1	4	3		
73	*Bombus vestalis*		1								
74	*Ceratina acuta*		1								
75	*Ceratina cucurbitina*		2								
76	*Ceratina cyanea*				1						
77	*Ceratina dallatorreana*	2									
78	*Ceratina nigrolabiata*	1									
79	*Chelostoma distinctum*									1	1
80	*Chelostoma florisomne*							1		1	
81	*Chelostoma rapunculi*			1	1						
82	*Coelioxys inermis*							1			
83	*Colletes cunicularius*				1					1	
84	*Colletes succinctus*			1	2				1		
85	*Dasypoda altercator*			1							
86	*Eucera chrysopyga*	2	1								
87	*Eucera clypeata*	1	2				1				
88	*Eucera dalmatica*		2								
89	*Eucera digitata*	1	3								
90	*Eucera nigrescens*	2		2			1				
91	*Eucera nigrifacies*		1								
92	*Eucera proxima*		1							3	1
93	*Eucera seminuda*		8								
94	*Eucera vulpes*	3	1								
95	*Halictus cochlearitarsis*	2	2								
96	*Halictus eurygnathus*	1		1							
97	*Halictus kessleri*	4	1			1	19				
98	*Halictus maculatus*		2	1		2	3				
99	*Halictus phryganicus*	1									
100	*Halictus pollinosus*			2	3						
101	*Halictus quadricinctus*					4	67				
102	*Halictus rubicundus*			3	6						
103	*Halictus scabiosae*			3	1						
104	*Halictus seladonius*		1								
105	*Halictus semitectus*		1								
106	*Halictus sexcinctus*	1			2		1				
107	*Halictus simplex*			2	4	2	21			6	4
108	*Halictus subauratus*						1				
109	*Halictus tumulorum*			15	11	1	1	7	1	4	8
110	*Hoplitis adunca*	1									
111	*Hoplitis claviventris*						1				
112	*Hoplitis leucomelana*					1					
113	*Hylaeus angustatus*		1								
114	*Hylaeus annularis*		1								
115	*Hylaeus annulatus*									1	
116	*Hylaeus brachycephalus*		1								
117	*Hylaeus breviceps*		1								

118	*Hylaeus brevicornis*				1		2				
119	*Hylaeus communis*			1	2						
120	*Hylaeus cornutus*						1				
121	*Hylaeus hyalinatus*				1						
122	*Hylaeus moricei*		2								
123	*Hylaeus punctatus*		1								
124	*Hylaeus sinuatus*			1							
125	*Hylaeus variegatus*					1					
126	*Lasioglossum albipes*					5	3	5	4	4	3
127	*Lasioglossum calceatum*			20	28				1	15	23
128	*Lasioglossum clypeare*				1	1					
129	*Lasioglossum costulatum*						1				
130	*Lasioglossum crassepunctatum*	7	17			1	1				
131	*Lasioglossum damascenum*	1					1				
132	*Lasioglossum fulvicorne*									30	35
133	*Lasioglossum glabriusculum*		14								
134	*Lasioglossum griseolum*	13	17								1
135	*Lasioglossum interruptum*					3	3			1	
136	*Lasioglossum laticeps*		43		2					88	54
137	*Lasioglossum lativentre*			3	4	3		1		2	
138	*Lasioglossum leucopus*		7	4	2			6	32		
139	*Lasioglossum leucozonium*			4	1					1	
140	*Lasioglossum lineare*					2	2			2	
141	*Lasioglossum lucidulum*		24							1	2
142	*Lasioglossum malachurum*	4	10	3	2	3	48				
143	*Lasioglossum marginatum*	1	3			1					
144	*Lasioglossum mesosclerum*						1				
145	*Lasioglossum minutulum*	2	1								
146	*Lasioglossum morio*			19	9	13	17	11	7	12	11
147	*Lasioglossum nigripes*					1	1				
148	*Lasioglossum nitidisculum*				1						
149	*Lasioglossum nitidiusculum*				1						
150	*Lasioglossum obscuratum*						1				
151	*Lasioglossum parvulum*				2						
152	*Lasioglossum pauperatum*	21	58			2	3				
153	*Lasioglossum pauxillum*	5	47	52	45	8	55				
154	*Lasioglossum politum*			2	1	4	29			1	5
155	*Lasioglossum puncticolle*	1	11								1
156	*Lasioglossum quadrinotatum*							11	7		
157	*Lasioglossum rufitarse*								1		
158	*Lasioglossum setulosum*		1							3	
159	*Lasioglossum sexnotatum*	1		8	2						
160	*Lasioglossum trichopygum*						3				
161	*Lasioglossum truncaticolle*		1								
162	*Lasioglossum villosulum*	1	3								
163	*Lasioglossum xanthopus*				1	1	2	1			2
164	*Lasioglossum zonulum*						1				
165	*Megachile circumcincta*			2							
166	*Megachile ericetorum*						4				
167	*Megachile genalis*				1						
168	*Megachile leachella*		1								
169	*Megachile pyrenaea*			1							
170	*Megachile willughbiella*				1						
171	*Melitta leporina*							1			
172	*Melitta tricincta*							1			
173	*Nomada bispinosa*	1	1								
174	*Nomada flavoguttata*	1		1			1				
175	*Nomada fucata*			3							
176	*Nomada goodeniana*	1	1								
177	*Nomada roberjeotiana*								1		
178	*Nomada ruficornis*				1						
179	*Nomada sexfasciata*									1	

180	*Nomada sheppardana*	1	5								
181	*Nomada stigma*	1									
182	*Nomada zonata*				1						
183	*Osmia aurulenta*					1					
184	*Osmia bicornis*				4			1		4	
185	*Osmia brevicornis*			1	4						
186	*Osmia jason*	1	1								
187	*Osmia leaiana*	4									
188	*Osmia rufohirta*	3				1	1				
189	*Osmia uncinata*							1			
190	*Sphecodes crassus*		6								
191	*Sphecodes ephippius*			2	1					1	
192	*Sphecodes monilicornis*			2							
193	*Sphecodes pseudofasciatus*		5								
194	*Stelis signata*									1	
195	*Systropha planidens*	6	3								
**Total abundance**		112	338	217	208	81	366	512	423	211	197
**Total richness**		44	55	47	55	33	49	30	25	35	32
**Shared species**		23		31		21		17		17	

Table S13: Carabid species recorded in grasslands (GR) and oilseed rape fields (OSR) across all countries (Bulgaria, Germany, Romania, Sweden and Switzerland) with total abundances. Green and yellow highlights indicate species found predominantly in the grasslands or oilseed rape fields, respectively, in the respective country (significant two-sided paired t-test; $p < 0.05$) and are thus influential for differences between habitats.

		Bulgaria		Germany		Romania		Sweden		Switzerland	
	**species**	**GR n = 8**	**OSR n = 8**	**GR n = 5**	**OSR n = 5**	**GR n = 7**	**OSR n = 7**	**GR n = 10**	**OSR n = 10**	**GR n = 6**	**OSR n = 6**
1	*Abax carinatus*					1					
2	*Abax parallelepipedus*									1	
3	*Abax parallelus*					1					
4	*Acinopus megacephalus*	51	30								
5	*Acinopus picipes*	15	27								
6	*Acupalpus elegans*			1							
7	*Acupalpus exiguus*				2			1			
8	*Acupalpus interstitialis*		4				11				
9	*Acupalpus meridianus*		3		1		2				4
10	*Agonum muelleri*								88		236
11	*Agonum obscurum*							6	1		
12	*Agonum piceum*				1						
13	*Agonum versutum*							4	1		
14	*Agonum viridicupreum*		1								
15	*Amara aenea*	68	178	124	58	25	18	73	56	24	9
16	*Amara anthobia*	3	1								
17	*Amara apricaria*		1						4		
18	*Amara arenaria*			4							
19	*Amara aulica*				1						
20	*Amara bifrons*							2	4		
21	*Amara chaudoiri*		3				1				
22	*Amara communis*		3	17	2		19	14	16		
23	*Amara convexior*			13	3					4	
24	*Amara equestris*			3			1				
25	*Amara eurynota*						4				9
26	*Amara familiaris*	1	2	23	130		5	20	94		2
27	*Amara fulva*		1		5						
28	*Amara fulvipes*	2	1	6							
29	*Amara littorea*							2			
30	*Amara lucida*	2				1	2	3	1		
31	*Amara lunicollis*			76	12			11			
32	*Amara majuscula*							1			
33	*Amara montivaga*			3							
34	*Amara ovata*	1	7	4	205	4	121				565
35	*Amara plebeja*			3	4			5	15		
36	*Amara proxima*										1
37	*Amara saphyrea*	1	1				1				
38	*Amara similata*		17	4	210	2	304	52	431		293
39	*Amara spreta*							15			
40	*Amara tibialis*			1	2			3	4		
41	*Amblystomus metallescens*		1								
42	*Amblystomus rectangulus*		1								
43	*Anchomenus dorsalis*		278	8	243	12	825	62	2321	2	818
44	*Anisodactylus binotatus*				5				1	1	34
45	*Anisodactylus signatus*						16				
46	*Apotomus clypeonitens*	3	1								
47	*Asaphidion flavipes*				83			2	38		4
48	*Badister bullatus*			2				5	1	3	
49	*Badister lacertosus*			1							
50	*Badister sodalis*						1				2
51	*Badister unipustulatus*				1						
52	*Bembidion aeneum*							1	5		
53	*Bembidion deletum*										1
54	*Bembidion guttula*							21	8		
55	*Bembidion lampros*			28	110		1	203	607		52
56	*Bembidion mannerheimii*			1							

57	*Bembidion obtusum*				21			15	40		2
58	*Bembidion properans*	5	12	33	31	1				1	42
59	*Bembidion quadrimaculatum*			2	22				3		54
60	*Bembidion tetracolum*				5			2	123		
61	*Brachinus alexandri*		1								
62	*Brachinus bodemeyeri*						1				
63	*Brachinus crepitans*	7	2			5	222			2	13
64	*Brachinus ejaculans*		33								
65	*Brachinus elegans*		9				491				
66	*Brachinus explodens*	30	174	1	32	1	998			3	159
67	*Brachinus nigricornis*		1								
68	*Brachinus psophia*		102								
69	*Calathus ambiguus*		4		168		1				
70	*Calathus cinctus*	2	3		70						
71	*Calathus erratus*								75		
72	*Calathus fuscipes*	70	15	249	2997	74	185	288	1958	17	5
73	*Calathus melanocephalus*			6	2		1	26	114		
74	*Calathus micropterus*							9	23		
75	*Calathus mollis*				1						
76	*Callistus lunatus*					1				4	
77	*Calosoma auropunctatum*	1	670				10				
78	*Calosoma sycophanta*		2								
79	*Carabus auratus*			1	5					176	
80	*Carabus auronitens*										1
81	*Carabus cancellatus*					2	9				
82	*Carabus convexus*		1	1			3				
83	*Carabus coriaceus*	18	34			23	15	31	5	3	
84	*Carabus glabratus*							1	2		
85	*Carabus granulatus*		2	12	57			84	443		1
86	*Carabus hampei*						1				
87	*Carabus hortensis*			5	1			2			
88	*Carabus montivagus*	1	2								
89	*Carabus nemoralis*			11	4			110	84	1	
90	*Carabus purpurascens*									21	
91	*Carabus scabriusculus*					1	1				
92	*Carabus ulrichii*						1				
93	*Carabus violaceus*					9	11				
94	*Carabus wiedemanni*	4	1								
95	*Carterus angustipennis*	1									
96	*Carterus dama*		1								
97	*Carterus gilvipes*	1									
98	*Chlaenius aeneocephalus*	3	269								
99	*Chlaenius decipiens*	21	56			16	5				
100	*Chlaenius nigricornis*							1	1		
101	*Chlaenius vestitus*		1								
102	*Cicindela campestris*	8				1	1		1		
103	*Clivina fossor*			1	28		3	40	111		21
104	*Cychrus caraboides*			1	1			2			
105	*Cylindera germanica*					64	85				
106	*Demetrias atricapillus*				5			3	2		
107	*Diachromus germanus*		1	1	1					1	9
108	*Dicheirotrichus placidus*			1							
109	*Ditomus calydonius*	1									
110	*Dixus clypeatus*	11				2					
111	*Dixus eremita*	4									
112	*Dixus obscurus*	17	4								
113	*Dolichus halensis*					2	9		1		
114	*Dromius linearis*							1			
115	*Dyschirius globosus*							1	1		
116	*Dyschirius salinus*							1			
117	*Elaphrus cupreus*							1			
118	*Gynandromorphus etruscus*	7	18								

119	*Harpalus affinis*		4	13	127		16	14	195	5	294
120	*Harpalus albanicus*	5									
121	*Harpalus angulatus*	4									
122	*Harpalus attenuatus*	15									
123	*Harpalus calceatus*		1				3	1	1		1
124	*Harpalus caspius*	5	1			56	16				
125	*Harpalus cupreus*		20			2	8				
126	*Harpalus dimidiatus*	47								88	16
127	*Harpalus distinguendus*	40	479	1	57	1	56	2	43		
128	*Harpalus flavicornis*	163	26			2	1				
129	*Harpalus froelichi*	13									
130	*Harpalus fuscicornis*	5	2								
131	*Harpalus griseus*	7	1			8	21				
132	*Harpalus honestus*									2	
133	*Harpalus hospes*		5			6	6				
134	*Harpalus laevipes*			1							
135	*Harpalus latus*			31	2			18	13	1	
136	*Harpalus luteicornis*										1
137	*Harpalus politus*			1		2					
138	*Harpalus pumilus*	10		6				1			
139	*Harpalus punctatostriatus*	5									
140	*Harpalus pygmaeus*	14	13								
141	*Harpalus rubripes*	41	7	16	7	7	1	2	1	26	
142	*Harpalus rufipalpis*			3							
143	*Harpalus rufipes*	13	32	42	163	36	516	47	183	2	260
144	*Harpalus saxicola*	5									
145	*Harpalus serripes*	31	56	1							
146	*Harpalus signaticornis*	1	10	11	185		4	1	3		1
147	*Harpalus smaragdinus*	14	9			3					
148	*Harpalus subcylindricus*	18	12	18		7	3			10	
149	*Harpalus tardus*	27	9	5	4			7	8	1	
150	*Harpalus xanthopus*		1	1	1						
151	*Laemostenus cimmerius*		1								
152	*Laemostenus terricola*						1				
153	*Lebia chlorocephala*							1			
154	*Lebia humeralis*	6									
155	*Lebia scapularis*	3									
156	*Leistus ferrugineus*			7				12	3		
157	*Licinus depressus*		2								
158	*Limodromus assimilis*			2	83			1	130		1
159	*Loricera pilicornis*			8	406			11	1078		33
160	*Microlestes apterus*	1				1	1				
161	*Microlestes corticalis*	3	13								
162	*Microlestes fissuralis*	104	80								
163	*Microlestes fulvibasis*	30	11								
164	*Microlestes maurus*	49	6			12		2	1		
165	*Microlestes minutulus*	85	149	23	1	3		13	10	4	1
166	*Microlestes negrita*		25								
167	*Microlestes plagiatus*		1								
168	*Microlestes schroederi*		3								
169	*Molops piceus*									1	
170	*Myas chalybaeus*	1									
171	*Nebria brevicollis*	4	8	75	1281			87	491	1	2
172	*Notiophilus aestuans*		1	2	37			1	62		
173	*Notiophilus aquaticus*							4	57		
174	*Notiophilus biguttatus*			2	39				22		
175	*Notiophilus germinyi*					1	1	4	2		
176	*Notiophilus laticollis*	1									
177	*Notiophilus palustris*			2	4			19	28		
178	*Notiophilus substriatus*	2									
179	*Oodes heliopiodes*							2			
180	*Ophonus ardosiacus*										1

181	*Ophonus azureus*	5	13			17	2			8	
182	*Ophonus cribricollis*	43	24								
183	*Ophonus diffinis*	1	1								
184	*Ophonus laticollis*					1					
185	*Ophonus parallelus*	2									
186	*Ophonus puncticeps*			7		1					
187	*Ophonus puncticollis*					4					
188	*Ophonus rufibarbis*						1	5	1		
189	*Ophonus sabulicola*	30	2			1	8				
190	*Panagaeus bipustulatus*			2							
191	*Panagaeus cruxmajor*							2			
192	*Pangus scaritides*	1									
193	*Parophonus hirsutulus*	23	2								
194	*Parophonus laeviceps*	1	30								
195	*Parophonus maculicornis*	9	1							6	1
196	*Parophonus mendax*	8	15								
197	*Parophonus planicollis*		6								
198	*Patrobus atrorufus*								2		
199	*Pedius inquinatus*	4	6								
200	*Poecilus anatolicus*		9								
201	*Poecilus cupreus*		431	154	3722	1	690	835	3571	17	6877
202	*Poecilus cursorius*		114								
203	*Poecilus koyi*						2				
204	*Poecilus kugelanni*				1						
205	*Poecilus lepidus*				33			54	611		
206	*Poecilus puncticollis*		5								
207	*Poecilus punctulatus*				2						
208	*Poecilus versicolor*			916	90		2	71	18		
209	*Polystichus connexus*	1	1								
210	*Pterostichus anthracinus*		1								73
211	*Pterostichus diligens*							5			
212	*Pterostichus hungaricus / melas*					99	29				
213	*Pterostichus macer*	3	8				3				
214	*Pterostichus madidus*			4	35						
215	*Pterostichus melanarius*			42	1430		3	255	3154		600
216	*Pterostichus niger*			1	3			79	257		1
217	*Pterostichus nigrita*							4			
218	*Pterostichus oblongopunctatus*				2			6	2		
219	*Pterostichus ovoideus*					4					
220	*Pterostichus strenuus*				8			15	6		
221	*Pterostichus vernalis*			3	12			16	3		7
222	*Stenolophus abdomialis*		1								
223	*Stenolophus teutonus*										1
224	*Stomis pumicatus*							7			2
225	*Syntomus foveatus*			1							
226	*Syntomus obscuroguttatus*	2	30	16	1						
227	*Syntomus pallipes*	5									
228	*Syntomus truncatellus*							1			
229	*Synuchus vivalis*			1	2			6	16		
230	*Tachys bistriatus*	1	3								
231	*Tachys scutellaris*										12
232	*Tachyura hoemorroidalis*	6									
233	*Tachyura parvula*		1								
234	*Thalassophilus longicornis*				3						
235	*Trechus micros*								2		
236	*Trechus quadristriatus*	7	32	9	307		8	23	222		66
237	*Trechus secalis*			5				58	38		
238	*Zabrus tenebrioides*	12	5			3	3				
239	*Zuphium olens*		5								
**Total abundance**		1295	3713	2079	12577	526	4790	2816	16917	436	10588
**Total richness**		81	99	68	67	45	62	77	69	30	45
**Shared species**		56		47		32		59		15	

Supplementary Note 3: Alternative analyses for species richness and the effective number of species

In addition to the sample size-based analyses, we conducted sample coverage-based analyses for species richness and the effective number of species following Chao, et al. [11]. Here, diversity measures are corrected based on an estimated sample completeness in the assemblages derived from the individual shares of the species in the assemblages via extrapolation and rarefaction [11,12]. The share of singleton observations, i.e. species that occur with only one individual, in a sample / assemblage strongly affects these estimations as the estimation of sample completeness is based on the distribution of singletons, with the assumption that a complete inventory of an assemblage would result in no remaining singleton observations [12]. A high share of singletons will lead to an estimated low sample completeness and, consequently, to a high estimated alpha diversity. For the moment, it is only possible to estimate measures of alpha diversity, more precisely, the three Hill-Chao numbers, resembling species richness ($q = 0$), the Hill-Shannon diversity or effective number of species ($q = 1$) and the Hill-Simpson diversity ($q = 2$; not presented here). These diversity metrics can be derived from an asymptotic estimation of the maximum diversity values in the sampled community. The identity of overlooked or not detected species can, however, not be estimated and thus all assemblage metrics that are based on species identities, like the NMDS / PERMANOVA or the components of beta diversity analyses presented in the main text, cannot be performed coverage based. It should, however, be stated that the method has recently been extended to an indirect approximation of overall beta diversity via the estimation of alpha and gamma diversity [13]. Instead of estimating at a certain, standardised level of sample completeness, alpha diversity can also be estimated at a standardised sample of individuals, i.e. at differing sample completeness but equal abundances, to avoid the so called mass-effect which often causes diversity to be determined by absolute abundances in a sample [14].

Standardised to equal sample completeness

We estimated the sample completeness for each taxonomic group in each grassland and oilseed rape field using the function 'iNEXT3D' (package 'iNEXT.3D', version 1.0.5, Chao, et al. [11]). Estimations were performed on the presence absence level ('incidence-raw') for plants where only coverage data was available and on the 'abundance' level for butterflies, wild bees and carabids. The obtained estimated values differed, in parts, systematically between habitats and countries (Fig. S21, Table S14). Species richness and effective number of species for each

taxonomic group were then estimated at a standardised sample completeness, the one third quantile within range of the observed sample completeness values, with the function 'estimate3D'. The one third quantile was chosen as a conservative compromise, rather accepting the rarefaction of assemblages with high sample completeness than the potential over-extrapolation of assemblages at low sample completeness (Table S14). This resulted in an extrapolation in 33 % of the sites in plants, in 34 % of the sites in butterflies, in 34 % of the sites in wild bees and in 33 % of the sites in carabids and a rarefaction in the remaining sites (except for one site in plants where the observed sample coverage was the chosen sample coverage). The estimated diversity values were then used as responses in generalised linear mixed effects models fitted as described in the main text, using the same commands and the same fixed and random effects but a Gamma distribution with log link. Model fits were assessed as described in the main text and models did not violate their assumptions.

While this analyses in detail yielded different results than our sample size-based approach, the overall message of our analyses remains the same. Both habitats harbour and support considerable biodiversity, with permanent grasslands being not necessarily more important for biodiversity than crop fields across taxa. Landscape grassland amount had limited effects on the two measures of diversity. While the higher richness of wild bees and carabids found in the size-based approach was no longer found in the coverage-based analyses with standardised sample completeness, we also no longer found grasslands to be more species rich in butterflies, with both habitat types having a comparable number of species in these three taxa (Fig. S22; Table S15). Only the effective number of species of carabids was found to be higher in grasslands in the coverage-based analyses and now negatively related with increasing grassland amount at both spatial scales (Fig. S22; Table S16). These results thus complement but do not contradict the results obtained from the size-based approach presented in the main text.

There are, however, also some concerns with this approach that may have biased sample completeness estimates and thus the estimates of diversity obtained based on the estimated sample completeness. As the estimation of sample completeness predominantly depends on the distribution of singleton observations [12], we believe that diversity values may have, in parts, been overestimated. Despite our extensive sampling, we observed a considerable share of singletons across habitats and countries, in some cases reaching up to 100 percent of the observed species in a habitat (Table S17). The estimation assumes that an assemblage is static and can be fully sampled with sufficient effort. This is not really true for assemblages in diverse landscapes where different habitats host partly distinct species assemblages (as we show in the

main text) and species stochastically enter assemblages via spillover from neighbouring habitats [15,16]. In addition, repeated sampling in time does not necessarily increase sampling effort as species assemblages change over time based on the individual species' phenological niches. Each repeated sampling in time thus introduces new species that could not have been detected before and there is no further chance to record some species not detected prior that have already ceased activity in the respective year. These mechanisms lead to an inflation of singletons in observed assemblages that lead to an overestimation of diversity.

More importantly, we often observed a systematic difference in singletons between habitat types, which may resemble true differences in sample completeness but may also be caused by the nature of the respective habitats. It can, for example, be assumed that most butterflies present in grasslands also reproduce in this habitat and occur there in larger numbers, which leads to fewer observed singletons than in oilseed rape fields, where many of the observed butterflies were likely dispersing individuals spilling over from surrounding habitats or structures. The observed singletons, however, increase the estimate of butterfly diversity in oilseed rape fields, which leads to the disappearance of the difference in species richness and the conclusion that there was no difference in butterfly richness between the habitats. Based on our experience monitoring butterflies across habitats and in these specific habitats, we believe this result to be an artefact and to not resemble the true conditions. Similarly, carabids are commonly found to be much more abundant in crop fields than in semi-natural grasslands [15,17-19], which was also observed here (Fig. S5 & Table S13). While this pattern may arise from unequal sample completeness, it seems more likely that it is caused by the much higher productivity in the (fertilised) crop fields and the resulting increased availability of food sources. With a higher total abundance, the probability of singleton observations decreases, which is regarded as achieving absolute sample completeness in this framework [12]. Consequently, while sample completeness was commonly estimated at 100 % for carabids across oilseed rape fields, sample completeness in grasslands was estimated considerably lower due to the many observed singletons. This led to a higher estimated diversity in this habitat and the disappearance of the pattern observed in the size-based analyses. It should be noted that some of the estimated values appeared unusually high, e.g. an estimated 43 carabid species in a grassland in Germany (where 29 species were observed) and 56 carabid species in a Bulgarian oilseed rape field (where 49 species were observed) at the conservative one third quantile of 95.5 percent estimated sample completeness. Even at this conservative level, the estimated richness values are higher than the carabid richness usually observed in these habitats in comparable studies, even at considerably higher sampling intensities and longer sampling

intervals [15,18,20] and we believe our values are thus likely, in parts, overestimated which may bias patterns. This overestimation occurs due to the large variation in sample completeness. We have tried to compensate for this and limit the bias by estimating at a conservative, rather low sample completeness preferring rarefaction over extrapolation.

We thus believe that the results of these coverage-based analyses are not bias-free and need to be interpreted with caution as they may not be fully trustworthy. We nevertheless report these results here in addition to our sample size-based analyses as a complementary approach for transparency.

Standardised to equal individual numbers

Similarly to the analyses at standardised sample completeness described above, we estimated species richness and effective number of species for a standardised number of individuals across samples, accepting varying sample completeness in these samples (Fig. S23; Table S14). We set the number of individuals at which richness and effective number of species were estimated to the conservative one third quantile of the individuals collected across all samples for the respective taxon. This is, of course, not possible for plants where data was only available at the presence absence level. This resulted in an extrapolation in 31 % of the sites in butterflies, in 31 % of the sites in wild bees and in 33 % of the sites in carabids and a rarefaction in the remaining sites (except for two sites in butterflies and three sites in wild bees where the observed number of individuals was identical to the chosen number of individuals for standardisation). The estimated values were then used as responses in separate models as described above. This approach yielded almost identical results to the analyses at standardised sample completeness with minor changes in the effects of landscape grassland amount on wild bee richness at 2000 m (not marginally significant in this approach), on carabid richness at 1000 m (not marginally significant in this approach) and on the effective number of species of carabids at 1000 m (changed from marginally significant to significant in this approach; Fig. S24, Tables S18 & S19). These results, as the ones obtained via the estimation at equal sample completeness, thus do not disagree with the main results of the size-based analyses presented in the main text and do not disagree with the conclusions drawn from these.

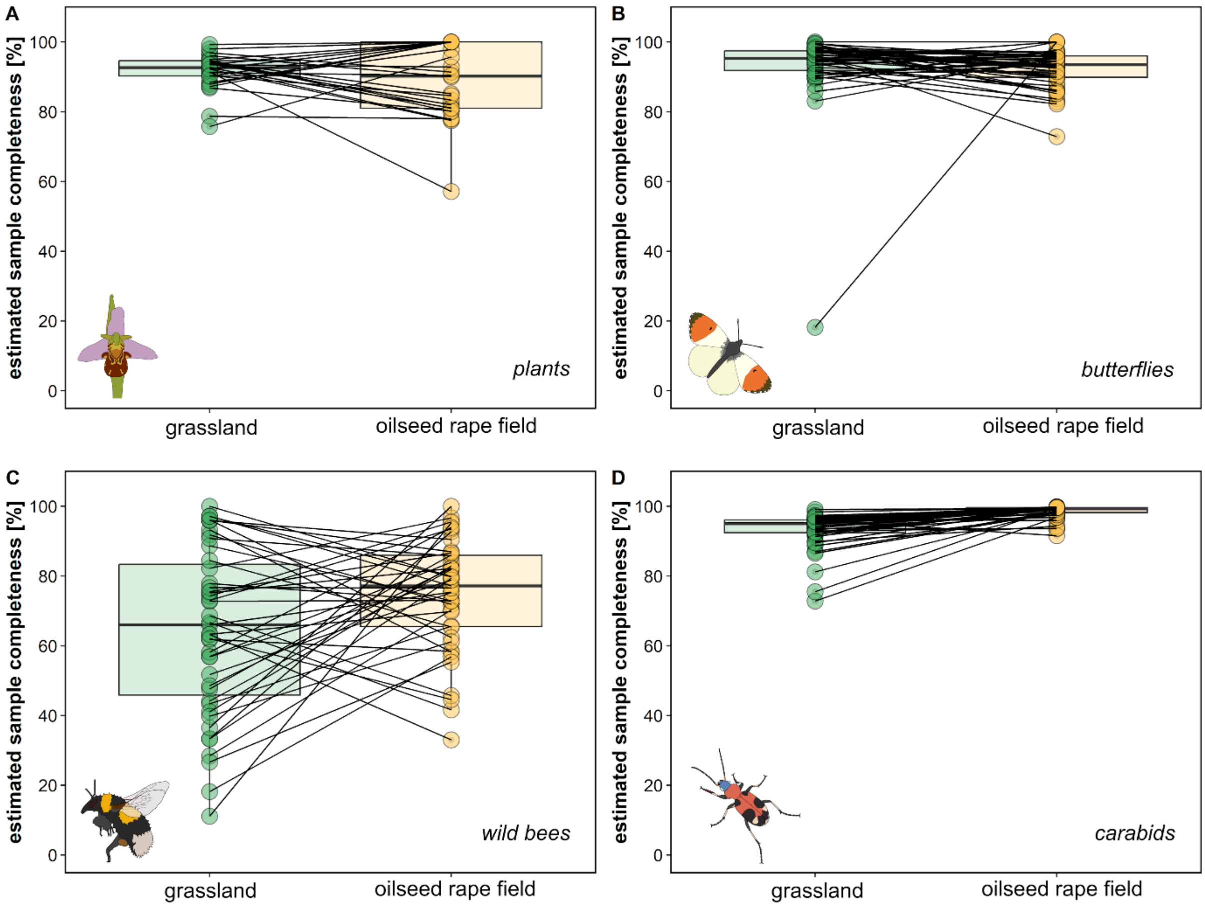

Fig. S21: Estimated sample completeness in each grassland (green) and oilseed rape field (yellow) for plants (A; n = 52), butterflies (B; n = 86), wild bees (C; n = 86) and carabids (D; n = 72). Estimates for plants were based on the presence-absence level ('incidence-raw'), estimated for the other taxa on the abundance level. Paired habitats are connected with black lines. For averages and ranges, overall and by country, see Table S14.

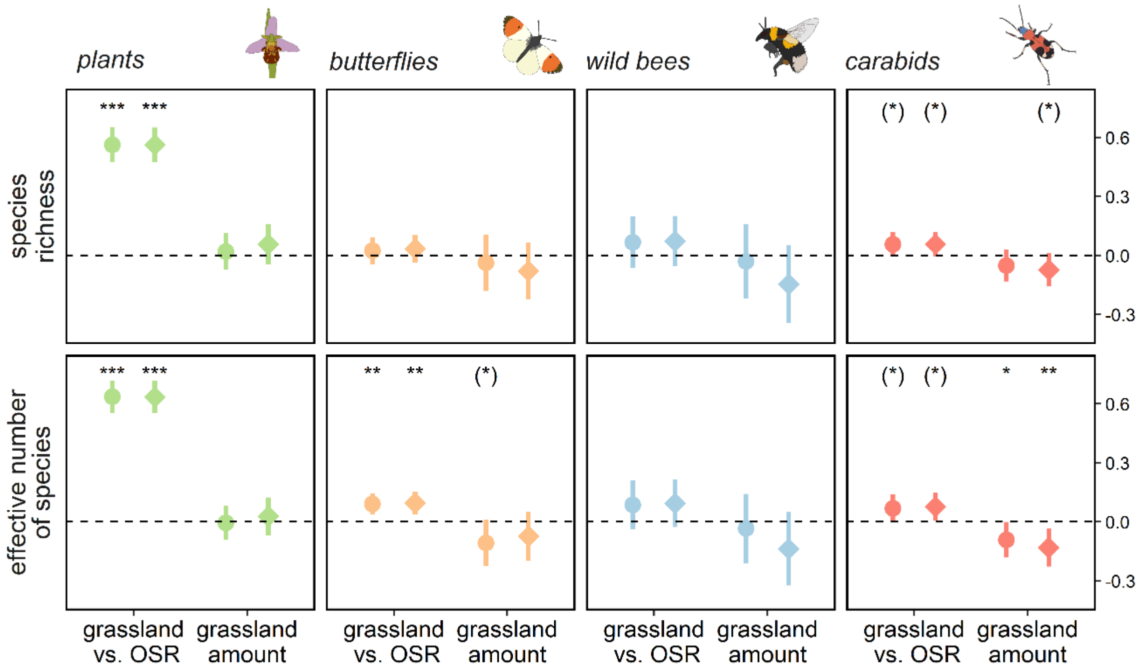

Fig. S22: Model coefficients for habitat type ('grassland vs. OSR'; positive values indicate a higher value in grasslands) and landscape grassland amount for species richness and effective number of species of plants, butterflies, wild bees and carabids at standardised sample completeness and at two spatial scales, 1000 m (circles) and 2000 m (diamonds). Coefficients with 95% confidence intervals. Coefficients from generalised linear mixed effects models are on the log-scale (species richness, effective number of species). (*) indicates $p < 0.1$, * $p < 0.05$, ** $p < 0.01$, *** $p < 0.001$. For statistics, see text and Tables S15 & S16.

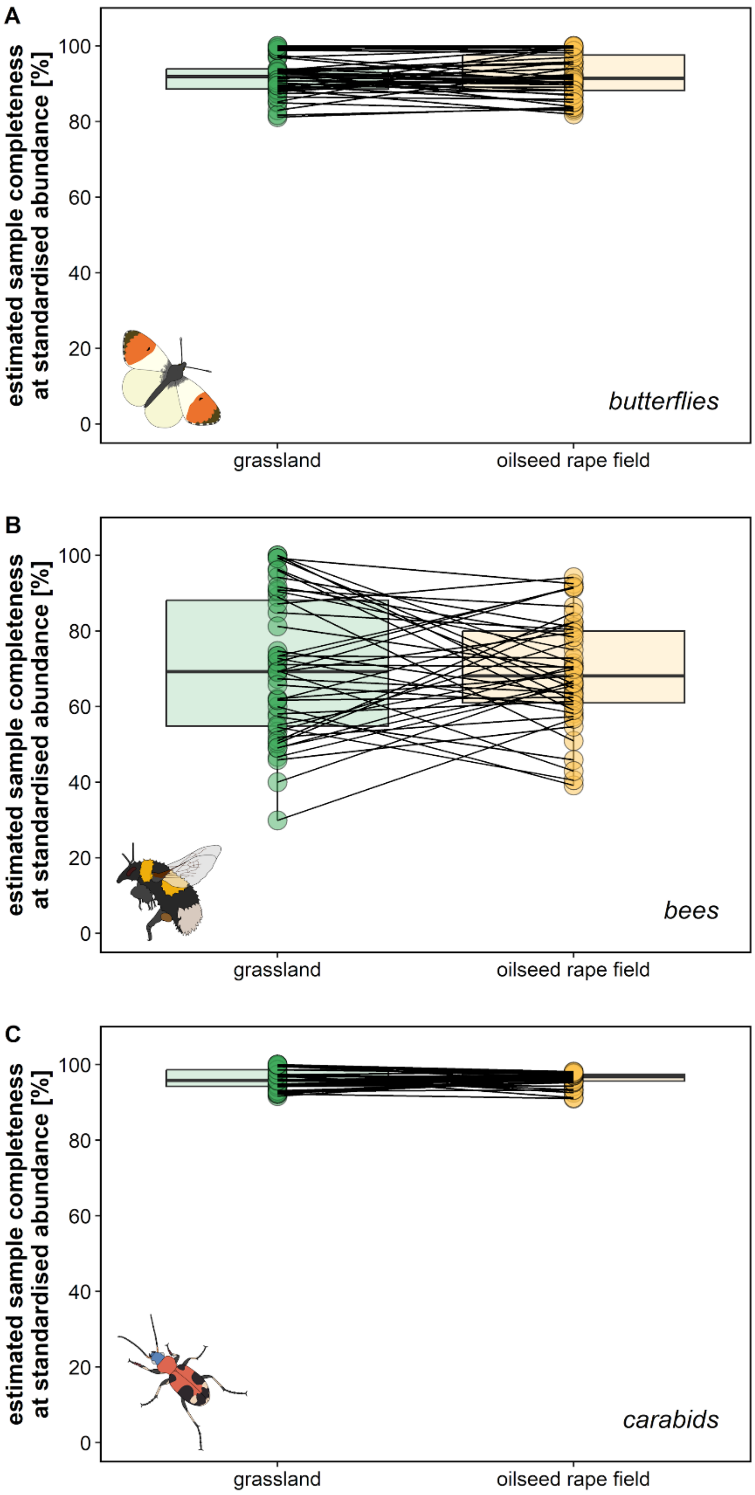

Fig. S23: Estimated sample completeness in each grassland (green) and oilseed rape field (yellow) for assemblages of butterflies (A; n = 86), wild bees (B; n = 86) and carabids (C; n = 72) at standardised abundance. Paired habitats are connected with black lines.

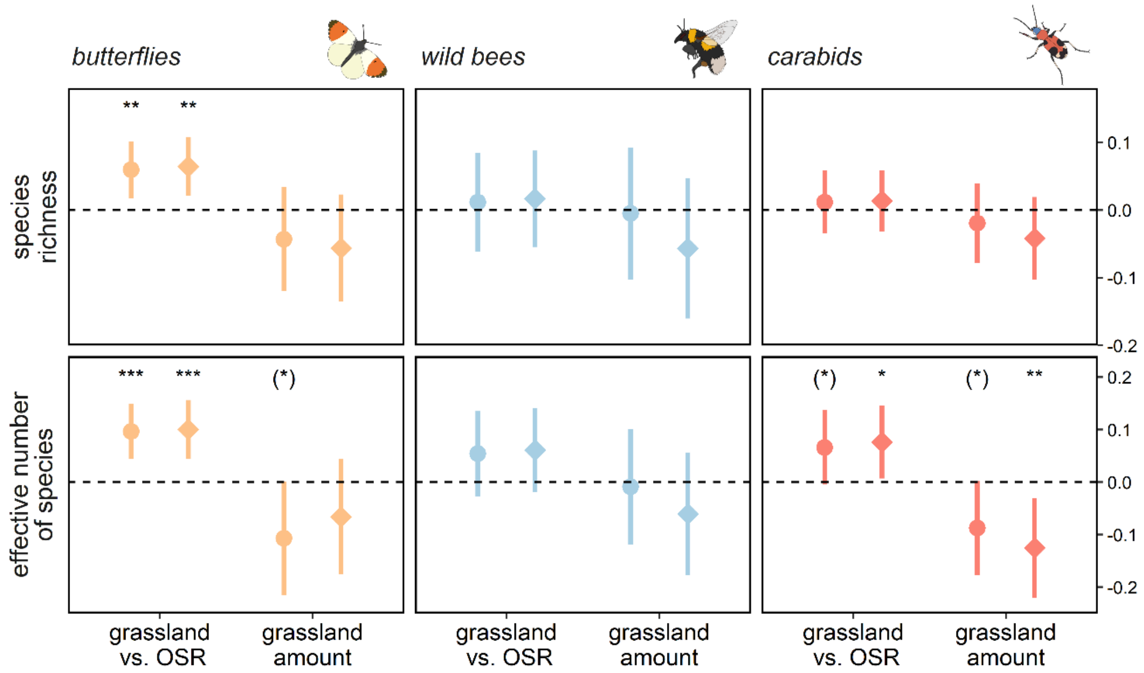

Fig. S24: Model coefficients for habitat type ('grassland vs. OSR'; positive values indicate a higher value in grasslands) and landscape grassland amount for species richness and effective number of species of butterflies, wild bees and carabids at standardised abundance and at two spatial scales, 1000 m (circles) and 2000 m (diamonds). Coefficients with 95% confidence intervals. Coefficients from generalised linear mixed effects models are on the log-scale (species richness, effective number of species). (*) indicates $p < 0.1$, * $p < 0.05$, ** $p < 0.01$, *** $p < 0.001$. For statistics, see text and Tables S18 & S19.

Table S14: Estimated overall sample completeness and across countries and habitats in the four taxonomic groups (average with range [minimum; maximum]) as well as the 1/3 quantile of sample completeness, the 1/3 quantile of abundance (rounded to full integers) used for standardisation and the estimated sample completeness at the 1/3 quantile of abundance (average with range). All values in percent, rounded to one decimal.

country	plants	butterflies	wild bees	carabids
**Bulgaria**		92.9 [83.7; 98.1]	69.9 [41.1; 100.0]	94.8 [89.9; 99.3]
grassland		93.3 [89.4; 98.1]	61.4 [41.1; 100.0]	94.1 [89.9; 96.1]
oilseed rape		92.5 [83.7; 97.6]	78.4 [65.6; 86.0]	95.5 [91.6; 99.3]
**Germany**	87.0 [77.5; 96.9]	93.9 [83.1; 97.0]	63.9 [18.2; 93.3]	97.2 [91.3; 99.9]
grassland	91.5 [78.7; 96.9]	93.9 [83.1; 97.0]	69.4 [18.2; 93.3]	95.0 [91.3; 99.1]
oilseed rape	82.4 [77.5; 91.4]	93.9 [89.8; 96.4]	58.4 [33.0; 85.8]	99.5 [98.9; 99.9]
**Romania**	87.3 [57.1; 94.8]	94.1 [82.8; 100.0]	61.0 [11.1; 96.7]	93.7 [72.8; 99.2]
grassland	92.9 [90.4; 94.8]	95.1 [89.6; 98.7]	47.8 [11.1; 82.2]	89.0 [72.8; 96.8]
oilseed rape	81.6 [57.1; 92.9]	93.1 [82.8; 100.0]	74.2 [44.6; 96.7]	98.4 [96.9; 99.2]
**Sweden**	95.1 [75.8; 100.0]	90.1 [18.2; 100.0]	80.1 [33.3; 100.0]	97.5 [92.4; 99.9]
grassland	90.8 [75.8; 99.3]	85.6 [18.2; 100.0]	76.5 [33.3; 97.2]	95.6 [92.4; 97.4]
oilseed rape	99.4 [95.7; 100.0]	94.5 [82.2; 100.0]	83.7 [62.4; 100.0]	99.3 [97.5; 99.9]
**Switzerland**		92.6 [72.9; 99.6]	71.2 [39.8; 97.1]	95.2 [75.5; 99.8]
grassland		98.3 [96.3; 99.6]	63.1 [39.8; 97.1]	90.9 [75.5; 97.0]
oilseed rape		86.9 [72.9; 95.5]	79.3 [61.2; 94.0]	99.4 [98.2; 99.8]
**overall**	90.2 [57.1; 100.0]	92.6 [18.2; 100.0]	69.6 [11.1; 100.0]	95.7 [72.8; 99.9]
grassland	91.6 [75.8; 99.3]	92.9 [18.2; 100.0]	64.3 [11.1; 100.0]	93.0 [72.8; 99.1]
oilseed rape	88.7 [57.1; 100.0]	92.3 [72.9; 100.0]	74.8 [33.0; 100.0]	98.5 [91.6; 99.9]
**1/3 quantile sample completeness**	88.5	92.2	62.7	95.5
**1/3 quantile of abundance**		46	14	183
**sample completeness at 1/3 quantile of abundance**		92.0 [81.1; 100.0]	69.6 [29.9; 100.0]	96.2 [91.0; 100.0]

Table S15: Type III Wald χ^2 tests for generalised linear mixed effects models of species richness, standardised at equal sample completeness, in relation to habitat type (semi-natural grassland vs. oilseed rape field), country and landscape permanent grassland cover at the 1000 m and 2000 m scales (separate models for the two scales) as well as their interactions across the four taxonomic groups (vascular plants, butterflies, wild bees and carabids). (*) indicates p < 0.10, * indicates p < 0.05, ** indicates p < 0.01, *** indicates p < 0.001. Df = degrees of freedom (numerator, denominator), F = F-value, p = p-value, R^2_m = marginal R^2 value.

Response	residual distribution	Df	χ^2	p	R^2_m
**Plants**					
1000 m scale					
habitat type	Gamma	1	155.18	**< 0.001** ***	0.86
grassland amount		1	0.17	0.679	
country		2	99.58	**< 0.001** ***	
grassland amount × habitat type		1	0.67	0.413	
grassland amount × country		2	1.79	0.409	
habitat type × country		2	19.28	**< 0.001** ***	
2000 m scale					
habitat type	Gamma	1	156.32	**< 0.001** ***	0.86
grassland amount		1	1.14	0.286	
country		2	89.97	**< 0.001** ***	
grassland amount × habitat type		1	0.08	0.782	
grassland amount × country		2	3.01	0.222	
habitat type × country		2	20.32	**< 0.001** ***	
**Butterflies**					
1000 m scale					
habitat type	Gamma	1	0.48	0.490	0.50
grassland amount		1	0.27	0.605	
country		4	38.31	**< 0.001** ***	
grassland amount × habitat type		1	5.07	**0.024 ***	
grassland amount × country		4	6.90	0.141	
habitat type × country		4	19.91	**< 0.001** ***	
2000 m scale					
habitat type	Gamma	1	0.86	0.355	0.54
grassland amount		1	1.13	0.287	
country		4	48.33	**< 0.001** ***	
grassland amount × habitat type		1	3.48	**0.062 (*)**	
grassland amount × country		4	10.45	**0.034 ***	
habitat type × country		4	19.51	**< 0.001** ***	
**Wild bees**					
1000 m scale					
habitat type	Gamma	1	0.98	0.323	0.36
grassland amount		1	0.10	0.751	
country		4	30.94	**< 0.001** ***	
grassland amount × habitat type		1	0.84	0.359	
grassland amount × country		4	0.20	0.995	
habitat type × country		4	4.39	0.356	
2000 m scale					
habitat type	Gamma	1	1.25	0.264	0.40
grassland amount		1	2.08	0.149	
country		4	30.95	**< 0.001** ***	
grassland amount × habitat type		1	0.01	0.913	
grassland amount × country		4	5.00	0.288	
habitat type × country		4	4.45	0.348	
**Carabids**					
1000 m scale					
habitat type	Gamma	1	3.09	**0.079 (*)**	0.79
grassland amount		1	1.60	0.206	
country		4	117.44	**< 0.001** ***	
grassland amount × habitat type		1	0.48	0.486	
grassland amount × country		4	5.23	0.264	
habitat type × country		4	28.04	**< 0.001** ***	
2000 m scale					
habitat type	Gamma	1	3.37	**0.067 (*)**	0.80
grassland amount		1	2.92	**0.088 (*)**	
country		4	106.34	**< 0.001** ***	
grassland amount × habitat type		1	4.23	**0.040 ***	
grassland amount × country		4	5.18	0.269	
habitat type × country		4	31.85	**< 0.001** ***	

Table S16: Type III Wald χ^2 tests for generalised linear mixed effects models of the effective number of species (i.e. the exponent of the Shannon–Wiener diversity index), standardised at equal sample completeness, in relation to habitat type (semi-natural grassland vs. oilseed rape field), country and landscape permanent grassland cover at the 1000 m and 2000 m scales (separate models for the two scales) as well as their interactions across the four taxonomic groups (vascular plants, butterflies, wild bees and carabids). (*) indicates p < 0.10, * indicates p < 0.05, ** indicates p < 0.01, *** indicates p < 0.001. Df = degrees of freedom (numerator, denominator), F = F-value, p = p-value, R^2_m = marginal R^2 value.

Response	residual distribution	Df	χ^2	p	R^2_m
**Plants**					
1000 m scale					
habitat type	Gamma	1	232.29	**< 0.001 *****	0.87
grassland amount		1	0.01	0.906	
country		2	67.71	**< 0.001 *****	
grassland amount × habitat type		1	0.23	0.635	
grassland amount × country		2	0.16	0.924	
habitat type × country		2	6.06	**0.048 ***	
2000 m scale					
habitat type	Gamma	1	233.15	**< 0.001 *****	0.87
grassland amount		1	0.31	0.579	
country		2	56.82	**< 0.001 *****	
grassland amount × habitat type		1	0.09	0.763	
grassland amount × country		2	0.67	0.714	
habitat type × country		2	7.25	**0.027 ***	
**Butterflies**					
1000 m scale					
habitat type	Gamma	1	10.73	**0.001 ****	0.46
grassland amount		1	3.14	**0.076 (*)**	
country		4	14.22	**0.007 ****	
grassland amount × habitat type		1	2.69	0.101	
grassland amount × country		4	8.64	**0.071 (*)**	
habitat type × country		4	18.89	**< 0.001 *****	
2000 m scale					
habitat type	Gamma	1	10.81	**0.001 ****	0.49
grassland amount		1	1.32	0.250	
country		4	18.96	**< 0.001 *****	
grassland amount × habitat type		1	1.30	0.254	
grassland amount × country		4	12.98	**0.011 ***	
habitat type × country		4	18.09	**0.001 ****	
**Wild bees**					
1000 m scale					
habitat type	Gamma	1	1.85	0.174	0.40
grassland amount		1	0.15	0.703	
country		4	39.37	**< 0.001 *****	
grassland amount × habitat type		1	1.25	0.263	
grassland amount × country		4	0.30	0.990	
habitat type × country		4	4.69	0.321	
2000 m scale					
habitat type	Gamma	1	2.33	0.127	0.42
grassland amount		1	2.09	0.148	
country		4	37.33	**< 0.001 *****	
grassland amount × habitat type		1	0.03	0.859	
grassland amount × country		4	3.63	0.458	
habitat type × country		4	3.76	0.439	
**Carabids**					
1000 m scale					
habitat type	Gamma	1	3.48	**0.062 (*)**	0.69
grassland amount		1	3.92	**0.048 ***	
country		4	69.60	**< 0.001 *****	
grassland amount × habitat type		1	1.27	0.260	
grassland amount × country		4	8.37	**0.079 (*)**	
habitat type × country		4	0.51	0.973	
2000 m scale					
habitat type	Gamma	1	4.69	**0.030 ***	0.70
grassland amount		1	7.05	**0.008 ****	
country		4	61.34	**< 0.001 *****	
grassland amount × habitat type		1	1.25	0.263	
grassland amount × country		4	8.18	**0.085 (*)**	
habitat type × country		4	0.68	0.954	

Table S17: Observed share of singleton observations (species with only one individual) across countries and habitats in the four taxonomic groups (average with range [minimum; maximum. All values in percent, rounded to one decimal.

country	plants	butterflies	wild bees	carabids
**Bulgaria**		40.9 [16.7; 66.7]	59.4 [0; 87.5]	35.5 [19.6; 50.0]
grassland		37.6 [16.7; 54.5]	62.1 [0; 87.5]	34.3 [26.1; 43.3]
oilseed rape		44.1 [26.7; 66.7]	56.8 [50.0; 63.6]	36.6 [19.6; 50.0]
**Germany**	38.1 [14.8; 60.0]	38.1 [18.8; 57.1]	64.1 [25.0; 100.0]	29.5 [11.4; 52.0]
grassland	30.2 [14.8; 53.1]	35.5 [18.8; 57.1]	59.1 [25.0; 100.0]	40.5 [25.0; 52.0]
oilseed rape	46.0 [28.6; 60.0]	40.7 [27.2; 54.5]	69.1 [57.9; 83.3]	18.5 [11.4; 25.0]
**Romania**	37.3 [23.3; 66.7]	31.0 [0; 69.2]	65.6 [20.0; 100.0]	40.4 [18.2; 66.7]
grassland	30.3 [23.3; 38.5]	28.1 [19.0; 48.0]	74.3 [40.0; 100.0]	45.0 [18.2; 66.7]
oilseed rape	44.3 [28.6; 66.7]	33.9 [0; 69.2]	56.9 [20.0; 81.8]	35.8 [21.7; 50.0]
**Sweden**	16.8 [0; 51.6]	28.5 [0; 100.0]	61.4 [12.5; 100.0]	28.9 [7.4; 46.7]
grassland	27.8 [5.9; 51.6]	33.9 [0; 100.0]	63.5 [33.3; 100.0]	35.4 [27.3; 46.7]
oilseed rape	5.8 [0; 25.0]	23.1 [0; 50.0]	59.3 [12.5; 80.0]	22.3 [7.4; 35.9]
**Switzerland**		35.3 [9.5; 57.1]	57.6 [25.0; 85.7]	32.9 [15.4; 66.7]
grassland		23.8 [9.5; 42.1]	63.9 [25.0; 85.7]	40.7 [25.0; 66.7]
oilseed rape		46.7 [23.1; 57.1]	51.2 [25.0; 63.6]	25.0 [15.4; 37.5]
**overall**	29.7 [0; 66.7]	34.5 [0; 100.0]	61.7 [0; 100.0]	33.2 [7.4; 66.7]
grassland	29.3 [5.9; 53.1]	31.9 [0; 100.0]	64.4 [0; 100.0]	39.0 [18.2; 66.7]
oilseed rape	30.1 [0; 66.7]	37.1 [0; 69.2]	58.9 [12.5; 83.3]	27.4 [7.4; 50.0]

Table S18: Type III Wald χ^2 tests for generalised linear mixed effects models of the species richness, standardised at equal abundance, in relation to habitat type (semi-natural grassland vs. oilseed rape field), country and landscape permanent grassland cover at the 1000 m and 2000 m scales (separate models for the two scales) as well as their interactions across the four taxonomic groups (vascular plants, butterflies, wild bees and carabids). (*) indicates p < 0.10, * indicates p < 0.05, ** indicates p < 0.01, *** indicates p < 0.001. Df = degrees of freedom (numerator, denominator), F = F-value, p = p-value, R^2_m = marginal R^2 value.

Response	residual distribution	Df	χ^2	p	R^2_m
**Butterflies**					
1000 m scale					
habitat type	Gamma	1	7.72	**0.005** **	0.57
grassland amount		1	1.20	0.273	
country		4	49.42	**< 0.001** ***	
grassland amount × habitat type		1	5.09	**0.024** *	
grassland amount × country		4	7.41	0.116	
habitat type × country		4	15.22	**0.004** **	
2000 m scale					
habitat type	Gamma	1	8.49	**0.004** **	0.59
grassland amount		1	1.93	0.165	
country		4	60.27	**< 0.001** ***	
grassland amount × habitat type		1	2.00	0.157	
grassland amount × country		4	11.20	**0.024** *	
habitat type × country		4	14.19	**0.007** **	
**Wild bees**					
1000 m scale					
habitat type	Gamma	1	0.10	0.757	0.28
grassland amount		1	0.01	0.919	
country		4	28.93	**< 0.001** ***	
grassland amount × habitat type		1	0.13	0.719	
grassland amount × country		4	0.56	0.967	
habitat type × country		4	1.87	0.760	
2000 m scale					
habitat type	Gamma	1	0.21	0.646	0.30
grassland amount		1	1.14	0.285	
country		4	28.32	**< 0.001** ***	
grassland amount × habitat type		1	0.22	0.640	
grassland amount × country		4	1.91	0.752	
habitat type × country		4	1.83	0.766	
**Carabids**					
1000 m scale					
habitat type	Gamma	1	0.25	0.620	0.77
grassland amount		1	0.42	0.519	
country		4	117.66	**< 0.001** ***	
grassland amount × habitat type		1	0.49	0.482	
grassland amount × country		4	3.35	0.501	
habitat type × country		4	23.98	**< 0.001** ***	
2000 m scale					
habitat type	Gamma	1	0.34	0.562	0.78
grassland amount		1	1.79	0.180	
country		4	106.93	**< 0.001** ***	
grassland amount × habitat type		1	2.21	0.137	
grassland amount × country		4	4.39	0.356	
habitat type × country		4	24.38	**< 0.001** ***	

Table S19: Type III Wald χ^2 tests for generalised linear mixed effects models of the effective number of species (i.e. the exponent of the Shannon–Wiener diversity index), standardised at equal abundance, in relation to habitat type (semi-natural grassland vs. oilseed rape field), country and landscape permanent grassland cover at the 1000 m and 2000 m scales (separate models for the two scales) as well as their interactions across the four taxonomic groups (vascular plants, butterflies, wild bees and carabids). (*) indicates p < 0.10, * indicates p < 0.05, ** indicates p < 0.01, *** indicates p < 0.001. Df = degrees of freedom (numerator, denominator), F = F-value, p = p-value, R^2_m = marginal R^2 value.

Response	residual distribution	Df	χ^2	p	R^2_m
**Butterflies**					
1000 m scale					
habitat type	Gamma	1	12.88	**< 0.001** ***	0.43
grassland amount		1	3.81	**0.051 (*)**	
country		4	9.14	**0.058 (*)**	
grassland amount × habitat type		1	1.30	0.254	
grassland amount × country		4	8.03	**0.091 (*)**	
habitat type × country		4	14.07	**0.007** **	
2000 m scale					
habitat type	Gamma	1	12.18	**< 0.001** ***	0.46
grassland amount		1	1.39	0.238	
country		4	11.75	**0.019** *	
grassland amount × habitat type		1	0.61	0.436	
grassland amount × country		4	12.98	**0.011** *	
habitat type × country		4	13.12	**0.011** *	
**Wild bees**					
1000 m scale					
habitat type	Gamma	1	1.72	0.190	0.38
grassland amount		1	0.02	0.877	
country		4	41.24	**< 0.001** ***	
grassland amount × habitat type		1	0.94	0.331	
grassland amount × country		4	0.58	0.965	
habitat type × country		4	3.81	0.432	
2000 m scale					
habitat type	Gamma	1	2.22	0.136	0.38
grassland amount		1	1.03	0.310	
country		4	38.28	**< 0.001** ***	
grassland amount × habitat type		1	0.79	0.373	
grassland amount × country		4	1.21	0.877	
habitat type × country		4	3.45	0.485	
**Carabids**					
1000 m scale					
habitat type	Gamma	1	3.32	**0.069 (*)**	0.67
grassland amount		1	3.70	**0.054 (*)**	
country		4	59.94	**< 0.001** ***	
grassland amount × habitat type		1	1.13	0.289	
grassland amount × country		4	8.44	**0.077 (*)**	
habitat type × country		4	0.59	0.964	
2000 m scale					
habitat type	Gamma	1	4.65	**0.031** *	0.67
grassland amount		1	6.64	**0.010** **	
country		4	53.23	**< 0.001** ***	
grassland amount × habitat type		1	0.78	0.378	
grassland amount × country		4	8.29	**0.081 (*)**	
habitat type × country		4	0.868	0.929	

Supplementary References

1 Kleyer, M. *et al.* The LEDA Traitbase: a database of life-history traits of the Northwest European flora. *J. Ecol.* **96**, 1266-1274 (2008). https://doi.org/https://doi.org/10.1111/j.1365-2745.2008.01430.x

2 Middleton-Welling, J. *et al.* A new comprehensive trait database of European and Maghreb butterflies, Papilionoidea. *Scientific Data* **7**, 351 (2020). https://doi.org/10.1038/s41597-020-00697-7

3 Westrich, P. *Die Wildbienen Deutschlands*. Vol. 2, aktualisierte Auflage 824 (Verlag Eugen Ulmer, 2019).

4 Lindroth, C. H. *Ground beetles (Carabidae) of Fennoscandia. A zoogeographic study. Part 1: Specific knowledge regarding the species. (Translation of: Die fennoskandischen Carabidae.)*. (Smithsonian Institution Libraries and The National Science Foundation, 1992).

5 Boetzl, F. A. *et al.* Distance functions of carabids in crop fields depend on functional traits, crop type and adjacent habitat: a synthesis. *Proceedings of the Royal Society B: Biological Sciences* **291**, 20232383 (2024). https://doi.org/https://doi.org/10.1098/rspb.2023.2383

6 Tschanz, P., Vogel, S., Walter, A., Keller, T. & Albrecht, M. Nesting of ground-nesting bees in arable fields is not associated with tillage system per se, but with distance to field edge, crop cover, soil and landscape context. *J. Appl. Ecol.* **60**, 158-169 (2023). https://doi.org/10.1111/1365-2664.14317

7 Hoffmann, H., Peter, F., Herrmann, J. D., Donath, T. W. & Diekötter, T. Benefits of wildflower areas as overwintering habitats for ground-dwelling arthropods depend on landscape structural complexity. *Agriculture, Ecosystems & Environment* **314**, 107421 (2021). https://doi.org/10.1016/j.agee.2021.107421

8 Heinen, J., Smith, M. E., Emery, S. E., Lundin, O. & Bommarco, R. Within-field overwintering contributes to arthropod predator assemblages in arable fields irrespective of tillage intensity. *Agriculture, Ecosystems & Environment* **393**, 109822 (2025). https://doi.org/https://doi.org/10.1016/j.agee.2025.109822

9 Boetzl, F. A., Krimmer, E., Holzschuh, A., Krauss, J. & Steffan-Dewenter, I. Arthropod overwintering in agri-environmental scheme flowering fields differs among pollinators and natural enemies. *Agriculture, Ecosystems & Environment* **330**, 107890 (2022). https://doi.org/10.1016/j.agee.2022.107890

10 Boetzl, F. A. & Knapp, M. On the ambivalence of granivorous carabids: Weed seed bank regulators, potential crop pests or both? *Agriculture, Ecosystems & Environment* **376**, 109226 (2024). https://doi.org/https://doi.org/10.1016/j.agee.2024.109226

11 Chao, A. *et al.* Measuring temporal change in alpha diversity: A framework integrating taxonomic, phylogenetic and functional diversity and the iNEXT.3D standardization. *Methods Ecol. Evol.* **12**, 1926-1940 (2021). https://doi.org/https://doi.org/10.1111/2041-210X.13682

12 Chao, A. *et al.* Quantifying sample completeness and comparing diversities among assemblages. *Ecological Research* **35**, 292-314 (2020). https://doi.org/https://doi.org/10.1111/1440-1703.12102

13 Chao, A. *et al.* Rarefaction and extrapolation with beta diversity under a framework of Hill numbers: The iNEXT.beta3D standardization. *Ecol. Monogr.* **93**, e1588 (2023). https://doi.org/https://doi.org/10.1002/ecm.1588

14 Chao, A. *et al.* Rarefaction and extrapolation with Hill numbers: a framework for sampling and estimation in species diversity studies. *Ecol. Monogr.* **84**, 45-67 (2014). https://doi.org/https://doi.org/10.1890/13-0133.1

15 Schneider, G., Krauss, J., Boetzl, F. A., Fritze, M.-A. & Steffan-Dewenter, I. Spillover from adjacent crop and forest habitats shapes carabid beetle assemblages in fragmented semi-natural grasslands. *Oecologia* **182**, 1141-1150 (2016). https://doi.org/10.1007/s00442-016-3710-6

16 Harman, R. R. & Kim, T. N. Differentiating spillover: an examination of cross-habitat movement in ecology. *Proceedings of the Royal Society B: Biological Sciences* **291**, 20232707 (2024). https://doi.org/doi:10.1098/rspb.2023.2707

17 Toivonen, M. *et al.* Effects of crop type and production method on arable biodiversity in boreal farmland. *Agriculture, Ecosystems & Environment* **337**, 108061 (2022). https://doi.org/10.1016/j.agee.2022.108061

18 Boetzl, F. A., Krimmer, E., Krauss, J. & Steffan-Dewenter, I. Agri-environmental schemes promote ground-dwelling predators in adjacent oilseed rape fields: Diversity, species traits and distance-decay functions. *J. Appl. Ecol.* **56**, 10-20 (2019). https://doi.org/10.1111/1365-2664.13162

19 Madeira, F. *et al.* Spillover of arthropods from cropland to protected calcareous grassland – the neighbouring habitat matters. *Agriculture, Ecosystems & Environment* **235**, 127-133 (2016). https://doi.org/http://dx.doi.org/10.1016/j.agee.2016.10.012

20 Boetzl, F. A., Schneider, G. & Krauss, J. Asymmetric carabid beetle spillover between calcareous grasslands and coniferous forests. *J. Insect Conserv.* **20**, 49-57 (2016). https://doi.org/10.1007/s10841-015-9838-6
