# Supplementary material for: Crop fields complement biodiversity in permanent grasslands across European landscapes
Source: Nat Commun. 2026 Jun 13;17:5263. doi: 10.1038/s41467-026-74356-7 (PMC13264617; doi:10.1038/s41467-026-74356-7)
Supplement: Supplementary file 2 — Reporting Summary [file 41467_2026_74356_MOESM2_ESM.pdf]

nature portfolio

Corresponding author(s): Fabian A. Boetzl

Last updated by author(s): May 4, 2026

Reporting Summary

Nature Portfolio wishes to improve the reproducibility of the work that we publish. This form provides structure for consistency and transparency in reporting. For further information on Nature Portfolio policies, see our Editorial Policies and the Editorial Policy Checklist.

Statistics

For all statistical analyses, confirm that the following items are present in the figure legend, table legend, main text, or Methods section.

n/a	Confirmed	
☐	☒	The exact sample size (*n*) for each experimental group/condition, given as a discrete number and unit of measurement
☐	☒	A statement on whether measurements were taken from distinct samples or whether the same sample was measured repeatedly
☐	☒	The statistical test(s) used AND whether they are one- or two-sided<br>*Only common tests should be described solely by name; describe more complex techniques in the Methods section.*
☐	☒	A description of all covariates tested
☐	☒	A description of any assumptions or corrections, such as tests of normality and adjustment for multiple comparisons
☐	☒	A full description of the statistical parameters including central tendency (e.g. means) or other basic estimates (e.g. regression coefficient) AND variation (e.g. standard deviation) or associated estimates of uncertainty (e.g. confidence intervals)
☐	☒	For null hypothesis testing, the test statistic (e.g. *F*, *t*, *r*) with confidence intervals, effect sizes, degrees of freedom and *P* value noted<br>*Give P values as exact values whenever suitable.*
☒	☐	For Bayesian analysis, information on the choice of priors and Markov chain Monte Carlo settings
☐	☒	For hierarchical and complex designs, identification of the appropriate level for tests and full reporting of outcomes
☐	☒	Estimates of effect sizes (e.g. Cohen's *d*, Pearson's *r*), indicating how they were calculated

Our web collection on statistics for biologists contains articles on many of the points above.

Software and code

Policy information about availability of computer code

Data collection	n/a
Data analysis	We solely used previously published code within the Software R (version 4.4.1) and the related packages 'glmmTMB' version 1.1.7), 'betapart' (version 1.6), 'vegan' (version 2.6-4),'DHARMa' (version 0.4.6), 'car' (version 3.1-2) and 'performace' (version 0.10.4).<br>No novel code was developed for this manuscript.

For manuscripts utilizing custom algorithms or software that are central to the research but not yet described in published literature, software must be made available to editors and reviewers. We strongly encourage code deposition in a community repository (e.g. GitHub). See the Nature Portfolio guidelines for submitting code & software for further information.

Data

Policy information about availability of data

All manuscripts must include a data availability statement. This statement should provide the following information, where applicable:

- Accession codes, unique identifiers, or web links for publicly available datasets
- A description of any restrictions on data availability
- For clinical datasets or third party data, please ensure that the statement adheres to our policy

All analyses were performed in R 4.4.1. The fully reproducible workflow, including an R markdown file with the code and all data files, will be available from Zenodo at https://doi.org/10.5281/zenodo.14044711

Research involving human participants, their data, or biological material

Policy information about studies with human participants or human data. See also policy information about sex, gender (identity/presentation), and sexual orientation and race, ethnicity and racism.

Reporting on sex and gender	n/a
Reporting on race, ethnicity, or other socially relevant groupings	n/a
Population characteristics	n/a
Recruitment	n/a
Ethics oversight	n/a

Note that full information on the approval of the study protocol must also be provided in the manuscript.

Field-specific reporting

Please select the one below that is the best fit for your research. If you are not sure, read the appropriate sections before making your selection.

☐ Life sciences ☐ Behavioural & social sciences ☒ Ecological, evolutionary & environmental sciences

For a reference copy of the document with all sections, see nature.com/documents/nr-reporting-summary-flat.pdf

Ecological, evolutionary & environmental sciences study design

All studies must disclose on these points even when the disclosure is negative.

Study description	Our design consisted of 86 paired permanent grasslands and oilseed rape fields (each pair nested within the same landscape) in five European countries. We assessed how habitat type (grassland vs. oilseed rape field) and the amount of grassland in the surrounding landscapes affected plant, butterfly, wild bee, and carabid beetle diversity and assemblages and tested whether increasing permanent grassland amount in the landscape can enrich biodiversity in crop field, i.e. the oilseed rape fields. Each of the taxonomic groups was sampled repeatedly throughout the season and with several samples in each habitat (detailed methods varied between taxonomic groups) but the data was pooled on the habitat level within each pair for analyses (i.e. 86 datapoints).
Research sample	We sampled the diversity of vascular plants, butterflies, wild bees and carabid beetles. These taxa differ in their ecology, e.g. in how easily they disperse throughout landscapes. Many species in all of these taxonomic groups are habitat specialists. These groups are thus commonly used as indicator taxa for biodiversity in comparable studies. By jointly analyzing trends across the four taxonomic groups, we can derive nuanced conclusions.
Sampling strategy	Sampling was performed using a standardized design across the five countries for each taxonomic group. The design is oriented on customary sampling designs and sample sizes that are standard in the field and was designed by experts prior to the start of the field campaign.
Data collection	The data was collected in a big field campaign within each of the participating countries with different local teams assessing the different taxonomic groups within each of the countries. These teams followed a standardised, pre-defined methodology for each of the taxonomic groups that is described in detail in the methods section.
Timing and spatial scale	Data was collected locally within two paired habitats in each landscape between April and August 2017 and 2018, depending on the country. Depending on the taxonomic group, data was collected repeatedly, two to four times, depending on an expert assessment of the sample size needed to make conclusions. These sampling intervals were standardized by oilseed rape phenology across countries and took place between oilseed rape flowering and after oilseed rape harvest.
Data exclusions	We did not exclude any data except for in carabid beetles, where some pitfall traps were lost due to management or wild animals. The selected exclusion of limited amounts of data restored equal sample sizes in pairs where traps had been lost in one of the habitats which assured the quality and reproducibility of our research. The exclusion and its criteria are described in detail in the methods section.
Reproducibility	We assured quality control by replicating a common, standardized study design with standardized field protocols within each country and we believe that the broad spatial extent of our data and conservative analyses choices ensure reproducibility. Our additional, second analyses approach presented in ESM2 that confirmed the main results of the analyses presented in the main text further indicates a high reproducibility of our analyses. We did neither intend nor attempt to reproduce our field experiment due to workload, monetary costs and ethical concerns.
Randomization	Randomization was not relevant to our study - we controlled for potential biases by establishing a standardized, replicated field design across five countries following methods used in comparable studies and the usual standards in the field.

Blinding	Blinding was not relevant to our study - all data for each taxonomic group and country was collected by the same researchers assuring standardisation within country and taxonomic group. The data was not analysed by the researchers that collected the data in the field to reduce potential biases.

Did the study involve field work? [x] Yes [ ] No

Field work, collection and transport

Field conditions	Field conditions varied across countries but were standardised within the customary boundaries for active pollinator observations across countries (observations taken between 9.00 and 17.00 on warm (> 17°C), sunny days (> 30% sun) with no strong winds and no rain during the previous hour)
Location	Five regions in five European countries: Central Bulgaria (Thracian lowland and foothills of the Sarnena Sredna Gora mountain range), Eastern Germany (northern Saxony around Leipzig), Central Romania (Transylvanian Plateau around Cluj), Southern Sweden (Skåne), Northern Switzerland (Swiss Jura northwest of Zürich). Precise GPS coordinates are available from the authors upon reasonable request.
Access & import/export	Access to the study sites was granted by the land owners and samples were gathered and processed by local experts in the participating countries. No further permissions were needed for data collection in Bulgaria, Germany, Romania, Sweden, and Switzerland. In Germany, the authority issuing permits for the collection of specimens in the respective region, the Landesamt für Umwelt, Landwirtschaft und Geologie (LfULG) in Saxony, was a project partner and directly involved in the planning and execution. this removed the necessity of a separate permit as our partner LfULG is free to collect samples in the field in the region administered by them.
Disturbance	As our fieldwork was done in agricultural landscapes and managed habitats, we believe that the sampling did not cause significant additional disturbance in addition to the usual management of these habitats. Wherever possible, we conducted non-detrimental data collection, identifying specimens in the field wherever possible and releasing specimens back into the habitats after identification. If we had to collect specimens for safe identiication, we made sure to take as few samples as possible while maintaining a good replication in order to achieve a good data quality.

Reporting for specific materials, systems and methods

We require information from authors about some types of materials, experimental systems and methods used in many studies. Here, indicate whether each material, system or method listed is relevant to your study. If you are not sure if a list item applies to your research, read the appropriate section before selecting a response.

Materials & experimental systems

n/a	Involved in the study	
x		Antibodies
x		Eukaryotic cell lines
x		Palaeontology and archaeology
	x	Animals and other organisms
x		Clinical data
x		Dual use research of concern
	x	Plants

Methods

n/a	Involved in the study	
x		ChIP-seq
x		Flow cytometry
x		MRI-based neuroimaging

Animals and other research organisms

Policy information about studies involving animals; ARRIVE guidelines recommended for reporting animal research, and Sex and Gender in Research

Laboratory animals	No laboratory animals were used.
Wild animals	We collected wild bees and carabid beetles using active (wild bees: transect walks) and passive (wild bees: pan traps; carabids: pitfall traps) methods. Specimens were only collected where no species identification was possible in the field (most wild bees and all carabid beetles) and otherwise released immediately after identification in the field. If identification in the lab was necessary, specimens were killed in the field, taken to the lab for identification and subsequently stored in 70% ethanol. All recorded species can be found in the data publicly available from Zenodo at https://doi.org/10.5281/zenodo.14044711
Reporting on sex	n/a
Field-collected samples	The collected wild bees and carabid beetles are stored in 70% ethanol in the collections of the following organisations:<br>Université de Neuchâtel, Neuchâtel, Switzerland (wild bees)<br>Institute of Biodiversity and Ecosystem Research (IBER), Bulgarian Academy of Sciences (BAS), Sofia, Bulgaria (wild bees & carabid beetles)

	Swedish University of Agricultural Sciences (SLU), Uppsala, Sweden (wild bees & carabid beetles)
Ethics oversight	No ethical approval was needed.

Note that full information on the approval of the study protocol must also be provided in the manuscript.

Dual use research of concern

Policy information about dual use research of concern

Hazards

Could the accidental, deliberate or reckless misuse of agents or technologies generated in the work, or the application of information presented in the manuscript, pose a threat to:

No	Yes	
x		Public health
x		National security
x		Crops and/or livestock
x		Ecosystems
x		Any other significant area

Experiments of concern

Does the work involve any of these experiments of concern:

No	Yes	
x		Demonstrate how to render a vaccine ineffective
x		Confer resistance to therapeutically useful antibiotics or antiviral agents
x		Enhance the virulence of a pathogen or render a nonpathogen virulent
x		Increase transmissibility of a pathogen
x		Alter the host range of a pathogen
x		Enable evasion of diagnostic/detection modalities
x		Enable the weaponization of a biological agent or toxin
x		Any other potentially harmful combination of experiments and agents

Plants

Seed stocks	n/a
Novel plant genotypes	No plants were modified in our study. We assessed plant species diversity in the field, without collecting samples.
Authentication	n/a
