# Supplementary material for: Crop fields complement biodiversity in permanent grasslands across European landscapes
Source: Nat Commun. 2026 Jun 13;17:5263. doi: 10.1038/s41467-026-74356-7 (PMC13264617; doi:10.1038/s41467-026-74356-7)
Supplement: Supplementary file 3 — Transparent Peer Review file [file 41467_2026_74356_MOESM3_ESM.pdf]

nature portfolio

Peer Review File

Crop fields complement biodiversity in permanent grasslands across European landscapes

Corresponding Author: Dr Fabian Boetzl

Parts of this Peer Review File have been redacted as indicated to remove third-party material.

This file contains all reviewer reports in order by version, followed by all author rebuttals in order by version.

Version 0:

Reviewer comments:

Reviewer #1

(Remarks to the Author)
The authors present a study on the diversity and community composition of four different taxonomic groups in pairs of oilseed rape fields and permanent grasslands sampled across a large number of landscapes and countries. The authors show that species richness and diversity is not generally higher in permanent grasslands compared to crop fields and that crop fields support a species assemblage that is complementary to the ones in permanent grasslands. While I'm not very familiar with the specific literature on arthropod and plant diversity in agricultural landscapes and can hence not fully confirm the novelty of the approach, I can still see that the overall study design is unique and that the specific approach of comparing paired habitat types is not used that often. Hence, the authors make a valuable contribution to the existing knowledge on biodiversity in agricultural landscapes.

Overall, the manuscript is well structured and written in a concise and easy-to-understand way. The chosen methods to sample species are well established and suitable for the studied taxa and habitat types. The sampling effort was also sufficient to achieve a good coverage of species with different phenologies. The analyses are of a high quality, appropriate for the type of data and the study design. The authors also report a very comprehensive description of the different data and model checks they performed. Not only the sampling methods but also the analytical approach are described in sufficient detail to be reproduced or applied in a different setting.

The choice of figures and summary tables to present the results is appropriate for the type of models and the data, the quality of the figures and tables is also very high. Hence, I have no suggestions on improvement regarding the method section and the presentation of the results.

I do however see potential to improve the manuscript in the way the results are described in the text and how that relates to the introduction and the hypotheses:

In its current form, the results section reads like a long list of combinations from indices, predictor variables and taxa without any clear pattern or structuring. Why is it useful to look at species richness, effective number of species, richness measures based on sample completeness and beta diversity for all questions/hypotheses? The result section hence reads more like an exploratory exercise in the differences between indices and the influence of different scales. The manuscript would strongly profit from a clear choice of analysis for each of the hypotheses. Ideally, the authors would explain which index, spatial scale and model is the most useful to answer each hypothesis for each taxon. Then they can focus on those results, telling a story that is easier to follow and based on clear ecological mechanisms or principles. The results of the additional analyses (with other indices, etc.) can be shown in the appendix and ideally, one sentence on the robustness (or variability) in the results will suffice. If the results are depending strongly on the choice of index, this can then be discussed and related to other potential mechanisms at play. One idea could also be to restructure the results based on the four hypotheses from the introduction, rather than separating only the analyses for species richness and beta diversity. Maybe it would already help to have a separate section on "Robustness of the analyses" where for instance lines 138 to 146 can be moved to.

In line 172, it was also not clear if the effect of country should be considered as an important result or if this statement is only showing how robust or variable the results are. In fact, differences between countries are not very surprising given the

differences in many abiotic and biotic characteristics between countries.

In addition to establishing a stronger link between the hypotheses and the respective main models, the authors should also make sure to provide a clear explanation/justification for each hypothesis. In particular, the lack of justification was apparent to me with hypotheses II and III. Ideally, the authors would link each hypothesis to an ecological mechanism or known pattern, e.g. the classic species-area-relationship could explain why a positive effect of grassland amount on biodiversity is expected.

The abstract also reflects the exploratory nature of the manuscript: Is this manuscript about the difference between crops and non-crops or about grassland amount? If the former, why only look at grasslands as non-crop habitat? If the latter, it should be made clear what the gradient of grassland cover was. I am also missing a clear motivation to the study approach. The fact that something is poorly studied, does not mean that is interesting to investigate alone.

Another point where I see a clear mismatch between the introduction, the results and the discussion is the statement that "a quantification of the relative contribution of crop habitats to biodiversity" has not been done yet (e.g. line 261). This argument was already raised in the Introduction, but I don’t really see a quantification of the relative contribution in this manuscript as well. The results mainly show that there are differences in species richness and that species assemblages differ between crop types due to species turnover. The only part of the manuscript where one can see tangible quantitative differences in species richness between habitat types is the Supplementary Material. If there is not enough space in the main part of the manuscript, this is fine. However, if the authors consider this an important message of their manuscript, it needs to be given more consideration and description in the results.

Minor points:
* at several instances in the text, a closing parenthesis is missing after the version number of an R package
* in the supplement, it is not clear what 1000k means as a measure of habitat amount

Reviewer #2

(Remarks to the Author)
This study contributes to our understanding of how local habitat conditions and landscape composition and configuration affect the diversity of insects. The study mpresses by having sampled a large number of plots, 86 pairs of a grassland and a crop field (oilseed rape) each, in 43 landscapes. Studies on the relative effects of local vs. landscape-scale conditions are by now quite common and many have found that both the local habitat conditions as well as landscape composition and configuration affect local diversity in the plot. Many studies have also found that the spatial scale of the landscape relevant to species differs among taxa. So any new study on these topics needs to go beyond what we already know. As pointed out by the authors, there is a set of species that can occur in crop fields not all of which can live in grasslands. In addition, there are species that use both habitats, to varying degrees. Overall, the general expectation would be that species richness of the taxa investigated is higher in grasslands than in arable fields, so this would not be surprising.

Given that there are so many studies already, I would expect additional analyses to be more in-depth than other analyses. This is where the current study falls a short of expectations, as detailed below. In addition, your discussion section is really disappointing as I found it to be very superficial, trying to paint a very broad picture and ignoring all the interesting results you have that have to do with details of your results. In my view, all of this can be cured with a little additional analysis and a more thorough discussion. In my view, this paper would be a very important addition to Nature Communications if those additional analyses are done. At present the results are not wrong, but also not so exiting.

Here my main general points:

First, you find that species richness effects, and effects on alpha diversity measures are not so strong (but see below), while ‘species composition’ differs. This is shown by your beta-analysis results, and by your NMDS analysis. However, these measures are really abstract, and, I believe, not very interesting if left on their own. Fig. 2 is really interesting and I think your paper could make a real contribution if you go into more detail. What species (traits) are driving the distinctness? What are those carabids in the bottom right corner of 2d?
It would be particularly interesting to explore the species using both habitats, are those known to occur in both grasslands and crop area (eg among the carabids), or visitors to both habitats (i.e. foragers exploiting flowering resources among wild bee).

Second: do you really believe that rape crops support many wild bee species and hence contribute more to landscape-level diversity than grasslands? The same is true for Lepidoptera. I think it is absolutely necessary to distinguish between species that at least theoretically can complete their lifecycle within either of these habitats, and those that cannot nest there. For the butterflies and potentially for the wild bees, you could check whether the grasslands (or arable fields) host the plants that the species needs. What wild bee actually breed in any of two habitats?

Third: you could exploit the temporal (several observation times for wild bees and butterflies) and spatial variation (transect) for your data, to understand the species overlap between habitats. Are there species that you find at all times in the same habitat, plus (or not) in a different habitats? Do they only occur close to the margin?

Methods:

326: if I see this correctly, ‘country’ is ‘confounded’ (the same) as ‘landscape’ as there is one landscape only in each country? Otherwise, you would also need to control for ‘landscape’.

Results:
126: what do you mean by ‘positively related’? Correlated? How did you test this in a paired design?

135: while species richness of wild bees and carabids was higher in oilseed rape fields than in

140: your results change if you correct for sample completeness. What does this mean and how did this come about? It has something to do with your sampling effort, and also with the distribution of singletons and doubletons. You do not really discuss this even though you have a good discussion in Supp 2. However, there you point out that the sample completeness leads to ‘dubious’ results. Your data are real, however, and iNext is based on a theoretical distribution of species which may not reflect the nature of spill-over or other possibilities. So instead of reporting results with and without sample completeness and arguing that they basically show the same thing (they don’t) I would use the chance to tackle this issue. Maybe this should be done more extensively in a separate paper, but I think you need to understand yourself what correcting for sample completeness does to your results, and whether the assumption of iNext really apply to your case.
On the other hand, your sampling effort really differs among countries, for some reason, and your mixture of methods (e.g. pan trap plus observations) makes it difficult to standardize. I would carry out individual analyses for each method and with standardized sampling effort, to see if your patterns hold up to the current ‘everthing together’ analysis.

Version 1:

Reviewer comments:

Reviewer #1

(Remarks to the Author)
In their first version, the authors already provided a manuscript with high quality study design, methods and analyses. My main suggestions for improvement were related to the structure and focus of the Introduction and results section. The authors have taken those suggestions to heart and greatly improved the clarity, focus and main messages of the manuscript:

The motivation and ecological theory behind the hypotheses are now much clearer. It's also now much easier to understand why it is interesting to look at both a crop/non-crop comparison and at the effect of non-crop habitat amount in the Introduction.

Now that the results are clearly linked to the three hypotheses and focus on the main indicators and scales they are much easier to follow. With all the analyses being shown in the Supplementary Materials, the message is still both convincing and nuanced enough.

Overall, I have only minor comments which can easily addressed. Therefore, I suggest to accept the manuscript.

Line 127: "declining" should be "declines" or "reduces"
line 232: one "neither" can be removed
line 241 to 245: try splitting the sentences in parts as it is very long right now
line 254: remove "a" before "twelve"

Reviewer #2

(Remarks to the Author)
This revised study now more extensively analyses differences between oilseed rape fields and permanent grasslands of diversity and community composition of four different taxonomic groups in pairs of sampled across a large number of landscapes and countries. The authors show that species richness and diversity is not generally higher in permanent grasslands compared to crop fields and that crop fields support a species assemblage that is complementary to the ones in permanent grasslands. In addition, the manuscript now contains more information at the species level that helps to put the results into perspective. This manuscript is vastly improved and should be eventually be published in Nature Communications, but I think it can be made even stronger with some more revision. In particular, some of the analyses shown in the cover letter should be integrated into the paper/OSM. In addition, I do suggest some extra work. The points raised in my last review that were addressed satisfactorily (most points) will not not be mentioned below.

One information that is missing from your paper is the intensity with which your grasslands were managed – there is a huge difference between grasslands in diversity depending on whether they are grazed or mown. While you state that your grasslands were not fertilized (which is surprising), they could still be cut 1-3 times per year, or grazed at different intensities. My prediction would be that the difference between crop field and grassland increases with decreasing land use intensity in the grasslands, which you can test.

Re-reading the paper I thought it would be useful if you could refer to gamma-diversity of your landscapes (as you discuss

alpha and beta), and could also analyse how much beta contributes to gamma.

Additional analysis
I appreciate the extra text provided with explanations and detailed discussion of individual species. However, you do not really refer to this in your paper, and you omit some of the explanations and extra figures you provide within the cover letter, e.g. Figure R1 (only in the letter).
I suggest to re-structure the supplementary material into logical sections, instead of only labelling each figure and table consecutively. This allows you to group tables and figures together and allows you to add extra text.
For example, “Supplementary Material 1” could be the extended methods, with figures S1-1, S1-2 (instead of Fig. S1, S2) You could then put ‘Detailed discussion: Species driving habitat differences across countries’ as a separate ‘Supplementary material x’. This more detailed discussion could go along with Fig. S5. At present, your extra text gets lost.

Trait analysis
I am not really convinced by your argument that traits vary too much between countries and within species, so that an analysis is not possible. There are many global analyses that succeed doing such analysis. I would find it extremely interesting if you could say something about the differences between the species that prefer the different habitats, or occur in both. You hint at this when you say that some carabids are ‘agrophilous’, yet this is just a description of where they have been observed, mainly. For example, plants in rape fields are probably more annuals rather than perennials. More interesting is however, what traits allow the species to persist in the temporary (crop) vs. permanent fields – what butterfly traits make it possible that they can survive in the habitats? Phenology of the larvae? Overwintering strategy? I do not insist on such an analysis, you could do this in a separate paper, but it would make the current paper even stronger.

Temporal patterns
Again, very interesting (Fig R2) and should be added to paper/supplement. In fact, you could also calculate, for each species, a relative specialisation measure, as the number of times it was observed in one habitat vs. total number of observations. This would go beyond your current analysis. Analysing the temporal variation, and also the strength of specialisation in a little more detail would help us to understand better why those habitats complement each other to increase gamma diversity at the landscape scale.

Open Access This Peer Review File is licensed under a Creative Commons Attribution 4.0 International License, which permits use, sharing, adaptation, distribution and reproduction in any medium or format, as long as you give appropriate credit to the original author(s) and the source, provide a link to the Creative Commons license, and indicate if changes were made.
In cases where reviewers are anonymous, credit should be given to 'Anonymous Referee' and the source.
The images or other third party material in this Peer Review File are included in the article’s Creative Commons license, unless indicated otherwise in a credit line to the material. If material is not included in the article’s Creative Commons license and your intended use is not permitted by statutory regulation or exceeds the permitted use, you will need to obtain permission directly from the copyright holder.
To view a copy of this license, visit https://creativecommons.org/licenses/by/4.0/

Reviewer #1 (Remarks to the Author):

Comment 1: The authors present a study on the diversity and community composition of four different taxonomic groups in pairs of oilseed rape fields and permanent grasslands sampled across a large number of landscapes and countries. The authors show that species richness and diversity is not generally higher in permanent grasslands compared to crop fields and that crop fields support a species assemblage that is complementary to the ones in permanent grasslands. While I'm not very familiar with the specific literature on arthropod and plant diversity in agricultural landscapes and can hence not fully confirm the novelty of the approach, I can still see that the overall study design is unique and that the specific approach of comparing paired habitat types is not used that often. Hence, the authors make a valuable contribution to the existing knowledge on biodiversity in agricultural landscapes.

Overall, the manuscript is well structured and written in a concise and easy-to-understand way. The chosen methods to sample species are well established and suitable for the studied taxa and habitat types. The sampling effort was also sufficient to achieve a good coverage of species with different phenologies. The analyses are of a high quality, appropriate for the type of data and the study design. The authors also report a very comprehensive description of the different data and model checks they performed. Not only the sampling methods but also the analytical approach are described in sufficient detail to be reproduced or applied in a different setting.

The choice of figures and summary tables to present the results is appropriate for the type of models and the data, the quality of the figures and tables is also very high. Hence, I have no suggestions on improvement regarding the method section and the presentation of the results.

Response 1: We thank the reviewer for this positive feedback on our manuscript.

Comment 2: I do however see potential to improve the manuscript in the way the results are described in the text and how that relates to the introduction and the hypotheses:

In its current form, the results section reads like a long list of combinations from indices, predictor variables and taxa without any clear pattern or structuring. Why is it useful to look at species richness, effective number of species, richness measures based on sample completeness and beta diversity for all questions/hypotheses? The result section hence reads more like an exploratory exercise in the differences between indices and the influence of different scales. The manuscript would strongly profit from a clear choice of analysis for each of the hypotheses. Ideally, the authors would explain which index, spatial scale and model is the most useful to answer each hypothesis for each taxon. Then they can focus on those results, telling a story that is easier to follow and based on clear ecological mechanisms or principles. The results of the additional analyses (with other indices, etc.) can be shown in the appendix and ideally, one sentence on the robustness (or variability) in the results will suffice. If the results are depending strongly on the choice of index, this can then be discussed and related to other potential mechanisms at play. One idea could also be to restructure the results based on the four hypotheses from the introduction, rather than separating only the analyses for species richness and beta diversity. Maybe it would already help to have a separate section on "Robustness of the analyses" where for instance lines 138 to 146 can be moved to.

Response 2: We rephrased the results section based on the suggestions by the reviewer. The results section now also follows more closely our three hypotheses indicated by separate headlines. We now also only present the results for the 2000 m scale in the main text and moved the results for the 1000 m scale and the previous figures to Supplementary Material I in order to make the results section more concise and easier to follow.

Both species richness and the effective number of species are used as they represent different aspects of diversity. Species richness is inflated by singleton observations, i.e. stochastic encounters that can represent a majority of the species observed in a habitat. In contrast, the effective number of species is derived from the Shannon entropy and thus mainly based on the common species defining communities and can be interpreted as the number of equally abundant species necessary to obtain a community with the identical average proportional abundances. Both indices thus address diversity but from slightly different angles, focussing on the rare (species richness) or the common species (effective number of species) which also explains the differences in our results. We have added further context to clarify why both indices are informative here (L 141ff). The alternative analyses based on sample completeness uses the same two indices but tries to correct for varying detectability of species. This may solve some problems but also introduces novel problems which we have discussed in Supplementary Material II. The additional analyses are not needed but presented in addition for matters of transparency. We have removed the emphasis on this additional analysis in the main text.

Comment 3: In line 172, it was also not clear if the effect of country should be considered as an important result or if this statement is only showing how robust or variable the results are. In fact, differences between countries are not very surprising given the differences in many abiotic and biotic characteristics between countries.

Response 3: We agree that differences between countries are not surprising given the many differences in abiotic and biotic characteristics. Consequently, we do not think the effect of the country is an important result, but nevertheless something that should be reported. We have rephrased the sentence accordingly and removed the word ‘important’ (L195ff).

Comment 4: In addition to establishing a stronger link between the hypotheses and the respective main models, the authors should also make sure to provide a clear explanation/justification for each hypothesis. In particular, the lack of justification was apparent to me with hypotheses II and III. Ideally, the authors would link each hypothesis to an ecological mechanism or known pattern, e.g. the classic species-area-relationship could explain why a positive effect of grassland amount on biodiversity is expected.

Response 4: We linked our hypotheses more clearly now to assumed ecological mechanisms and theory, in particular the ‘habitat amount hypothesis’ (Fahrig, 2013) suggesting that increasing habitat amount increases local biodiversity (an adaptation of the classical species-area relationship) and the ‘landscape-moderated insurance hypothesis’ (Tscharntke et al., 2012), suggesting that semi-natural habitats supply and ensure biodiversity to and in crop fields via spillover (L 120ff). While Hypothesis 1 is generally based on the assumption that semi-natural habitats act as refuges for biodiversity and are thus more biodiverse as disturbed agricultural habitats, Hypothesis 2 results from the habitat amount hypothesis and the landscape-moderated insurance hypothesis while Hypothesis 3 is a conclusion of Hypotheses 1 and 2.

Comment 5: The abstract also reflects the exploratory nature of the manuscript: Is this manuscript about the difference between crops and non-crops or about grassland amount? If the former, why only look at grasslands as non-crop habitat? If the latter, it should be made clear what the gradient of grassland cover was. I am also missing a clear motivation to the study approach. The fact that something is poorly studied, does not mean that is interesting to investigate alone.

Response 5: The manuscript is about both factors mentioned by the reviewer, the difference between crop and non-crop habitats and especially on how grassland amount in the surrounding landscape affects or modulates this difference. The hypothesis is that grasslands host a higher biodiversity across taxa than crop fields but that this difference decreases with increasing grassland amount in the surrounding landscape as more individuals and species spill over into crop habitats with increasing amount of the grassland (source) habitat. We used grasslands as these are the predominant open semi-natural habitat habitats in the agricultural landscapes of Europe, and they are regarded as refuges and source habitats for the biodiversity that could potentially also be found in crop fields.

We have rephrased the abstract to better justify the aim of our study and better reflect the findings (L 69ff). While space is highly limited in the abstract, we rephrased it slightly to clarify that both habitat type and grassland amount are part of our research question and to point out that the motivation for our study was to inform biodiversity conservation in agricultural landscapes.

Comment 6: Another point where I see a clear mismatch between the introduction, the results and the discussion is the statement that "a quantification of the relative contribution of crop habitats to biodiversity" has not been done yet (e.g. line 261). This argument was already raised in the Introduction, but I don’t really see a quantification of the relative contribution in this manuscript as well. The results mainly show that there are differences in species richness and that species assemblages differ between crop types due to species turnover. The only part of the manuscript where one can see tangible quantitative differences in species richness between habitat types is the Supplementary Material. If there is not enough space in the main part of the manuscript, this is fine. However, if the authors consider this an important message of their manuscript, it needs to be given more consideration and description in the results.

Response 6: While the reviewer is correct that we do not provide a quantification in the classical way, the line referenced here is part of the conclusion and is indeed a call for further research to do such a quantification which would be direly needed (see L 317ff: ‘We call for a wider quantification of the relative contribution of different crop types under different management regimes to biodiversity as data is, for most crop types and especially crop-management combinations, largely missing.’). As the reviewer is correct and we did not really quantify the relative contribution, in the main text, we rephrased the mentioned sections in the introduction to make this clearer (e.g. L 110).

Minor points:

Comment 7: * at several instances in the text, a closing parenthesis is missing after the version number of an R package

Response 7: Corrected.

Comment 8: * in the supplement, it is not clear what 1000k means as a measure of habitat amount

Response 8: Clarified and corrected.

Reviewer #2 (Remarks to the Author):

This study contributes to our understanding of how local habitat conditions and landscape composition and configuration affect the diversity of insects. The study impresses by having sampled a large number of plots, 86 pairs of a grassland and a crop field (oilseed rape) each, in 43 landscapes. Studies on the relative effects of local vs. landscape-scale conditions are by now quite common and many have found that both the local habitat conditions as well as landscape composition and configuration affect local diversity in the plot. Many studies have also found that the spatial scale of the landscape relevant to species differs among taxa. So any new study on these topics needs to go beyond what we already know. As pointed out by the authors, there is a set of species that can occur in crop fields not all of which can live in grasslands. In addition, there are species that use both habitats, to varying degrees. Overall, the general expectation would be that species richness of the taxa investigated is higher in grasslands than in arable fields, so this would not be surprising.

Comment 9: Given that there are so many studies already, I would expect additional analyses to be more in-depth than other analyses. This is where the current study falls a short of expectations, as detailed below. In addition, your discussion section is really disappointing as I found it to be very superficial, trying to paint a very broad picture and ignoring all the interesting results you have that have to do with details of your results. In my view, all of this can be cured with a little additional analysis and a more thorough discussion. In my view, this paper would be a very important addition to Nature Communications if those additional analyses are done. At present the results are not wrong, but also not so exiting.

Response 9: We are very grateful to the reviewer for the positive feedback and the fruitful input that helped us add more depth to our manuscript. While the reviewer is correct that many studies have assessed biodiversity in crop fields in relation to landscape context, studies directly comparing biodiversity in different habitat types, especially biodiversity in crop fields to biodiversity in semi-natural habitats in close proximity, are lacking. Most studies addressing biodiversity in crop fields assume that semi-natural habitats act as source habitats for the species found in the crop fields (i.e. the ‘landscape-moderated insurance hypothesis’ (Tscharntke et al., 2012)) but do not actually assess this presumed relationship. Results from different studies assessing biodiversity in different habitat types are not directly comparable due to the heterogeneity in methodology, phenology and climate, and our results thus are a first step towards filling this important knowledge gap.

In response to the comments below, we have added a new species-by-species analysis of habitat preferences and enriched the discussion with more detailed results concerning the defining

characteristics of the species that drive the distinctness and complementarity of the habitats to strengthen our manuscript.

Here my main general points:

Comment 10: First, you find that species richness effects, and effects on alpha diversity measures are not so strong (but see below), while ‘species composition’ differs. This is shown by your beta-analysis results, and by your NMDS analysis. However, these measures are really abstract, and, I believe, not very interesting if left on their own. Fig. 2 is really interesting and I think your paper could make a real contribution if you go into more detail. What species (traits) are driving the distinctness? What are those carabids in the bottom right corner of 2d? It would be particularly interesting to explore the species using both habitats, are those known to occur in both grasslands and crop area (eg among the carabids), or visitors to both habitats (i.e. foragers exploiting flowering resources among wild bee).

Response 10: We thank the reviewer for this constructive input! We agree that informing our results with more specific information sheds more light on the filters shaping local biodiversity.

Detailed knowledge about functional traits and habitat preferences needed for such an analysis is limited and habitat preferences of species can and do vary across regions and countries (see e.g. habitat preference variation in carabids even within Germany summarised in Gesellschaft für Angewandte Carabidologie 2009) which raises questions about the transferability and applicability of literature records. Following the suggestion by the reviewer, we identified species with a preference for one of the two habitats that drive the distinctness of species assemblages. As habitat preferences of species and species pools vary across countries, we decided to perform a separate species-by-species analysis of habitat preferences for each of the five countries. Within each country and for each species, we used paired tests to assess whether a species was found significantly more often in one of the two paired habitats, identifying these species as species associated with this habitat type (automatically rendering all other species as species using both habitats equally). This approach using paired tests simultaneously reduces the impact of singletons / rare species occasionally encountered as they are usually only found in low numbers and on few sites. These species could easily be identified as habitat specialists but could equally likely resemble species spilling over from adjacent habitats. Using this approach, we identified 146 of the 837 species to be associated with either grasslands (82 species; 49 plant species, 21 butterfly species and twelve carabid species) or oilseed rape fields (63 species; seven plant species, six butterfly species, ten wild bee species and 40 carabid species) in any of the countries, with a large variation in associated species across the countries but also some species that were consistently associated with one habitat across several or even all countries (L 161ff; Supplementary Material I, Tables S4 to S7). We now discuss these species driving the distinctness of habitats as well as the traits they share in Supplementary Material I and added a summary of the overall patterns and the communal features of these species to the discussion (L 221ff). While the reviewer also raised an interesting point about the common characteristics of species using both habitat types, we believe that the large share of species using both habitats in our assessment makes such an analysis complicated and less informative (see the considerable overlap in Supplementary Material I, Tables S4 to S7; for more analyses concerning the temporal change of species overlap, see Response 12 below). We believe that

our analysis focussed on the species specialised to one of the two habitats does provide better insights into the drivers shaping species assemblages in both habitats.

Concerning the NMDS analysis, the large number of species involved in all taxa but butterflies complicates interpretations (Fig. R1). Our NMDS / PERMANOVA analyses are calculated based on relative abundances, and assemblage similarity is driven by both overlaps in species composition but also by similar abundance structure in the assemblages. Differences are thus not driven by species alone. As an example, the reviewer asked about the commonalities of the carabid species that push assemblages into the bottom right corner of the NMDS plot. When the species are plotted, we see that almost half of all carabid species accumulate towards the bottom right corner of the NMDS ordination (Fig. R1D). These species comprise typical agrophilous species like *Poecilus cupreus*, *Pterostichus melanarius*, *Amara similata* or *Harpalus affinis* that push oilseed rape assemblages in this direction but also species of the temperate, more moist grasslands like *Chlaenius nigricornis*, *Amara plebeja* and *Agonum versutum*. These species are all common in Northern and Central Europe and thus pull the sites from Germany and Sweden into this part of the plot. In contrast, the bottom left corner has many species restricted to the Balkans like *Carterus spp.*, *Poecilus cursorius* or *Laemostenus cimmerius* while the top right corner has many xerophilic species that can be found on grasslands, depending on local habitat conditions, but are less likely to occur in oilseed rape fields like *Harpalus politus* or *Pterostichus ovoideus*. The NMDS of the carabid assemblages thus closely resembles local climatic and habitat characteristics, separating countries by local species pools and / or separating species based on the climatic conditions limiting their distribution across countries. For example, *Pterostichus melanarius*, a carabid species common and often dominant in croplands across northern Europe is restricted to mountainous regions in Romania and Bulgaria and consequently largely absent from oilseed rape fields in Romania and completely absent from oilseed rape fields in Bulgaria (Supplementary Material I, Table S7), among other species shaping the NMDS ordination and causing the observed effect of country. Country, acting as proxy for climatic differences, differences in grassland management and differences in the composition of carabid species pools was also consistently identified as the most important predictor in these multivariate analyses (L 157f). These interwoven effects make the interpretation of this figure on the species level complicated. As we believe that differences across countries were to a large part caused by the distributional restrictions of individual species within Europe, the potential for interpretations of functional characteristics is limited. Nevertheless, we see a clear overall pattern of habitat shaping assemblages across countries, i.e. within each country, which, in our opinion, is the most interesting result here. In plants (Fig. R1A) and butterflies (Fig R1B), the patterns are much clearer as the assemblages are more separated across countries and the species associated with the centres of grassland or oilseed rape assemblage clusters are largely also the species identified as associated with the habitat in our new analyses presented above (L 157ff and Supplementary Material I, Tables S4 & S5). We thus believe that our new analysis of species-by-species habitat preferences described above covers the species defining the complementarity of the habitat types and we added nothing further here.

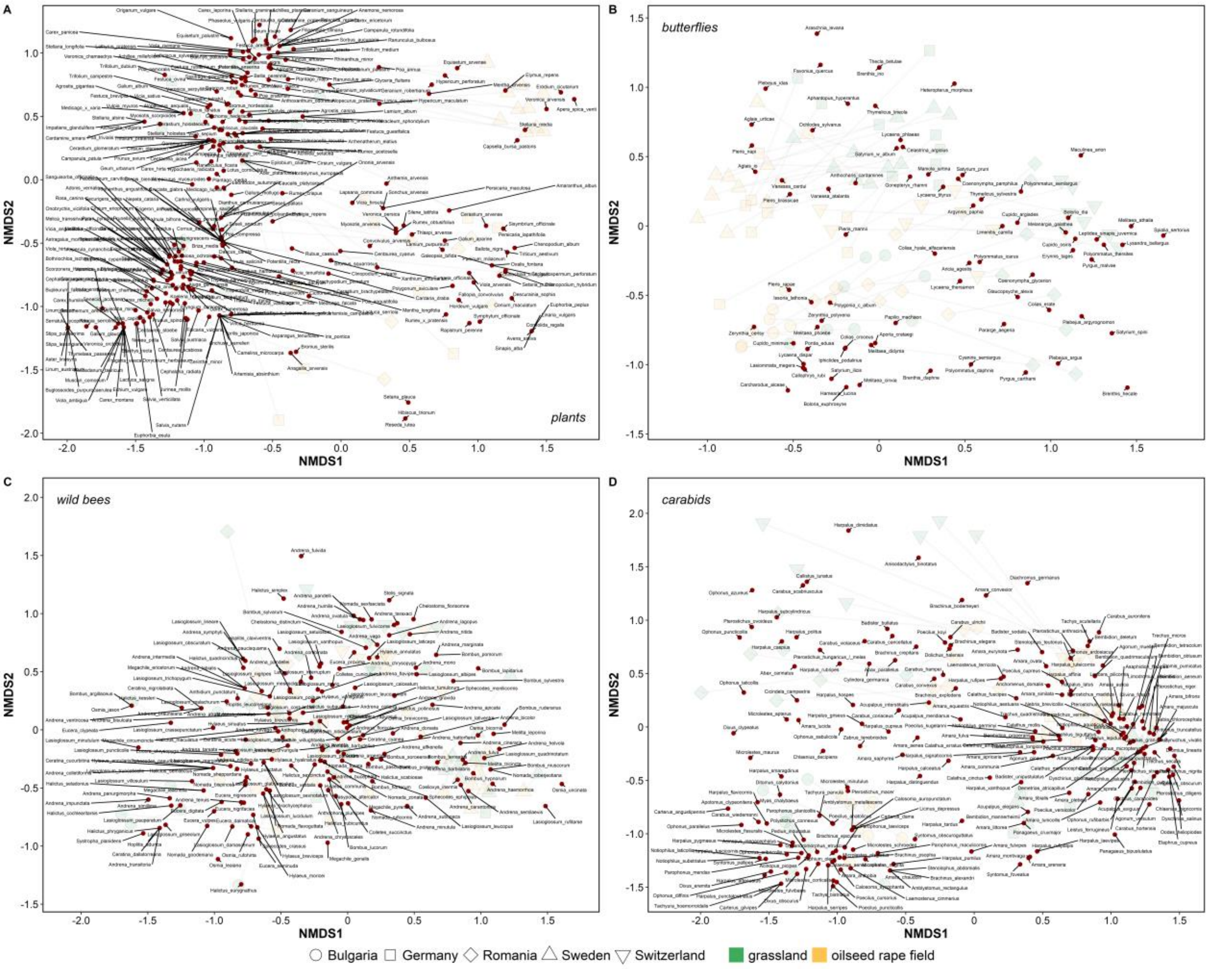

Fig. R1: Species assemblages of four taxonomic groups (NMDS ordination) in the two sampled habitat types (oilseed rape and grassland) across five European countries (Bulgaria, Germany, Romania, Sweden and Switzerland). The taxonomic groups comprised plants (A), butterflies (B) wild bees (C) and carabids (D). Red points indicate the individual species. The NMDS used Bray-Curtis distances on proportional abundances or proportions of cover (plants). For statistics, see text and Supplementary Material I, Table S10.

Comment 11: Second: do you really believe that rape crops support many wild bee species and hence contribute more to landscape-level diversity than grasslands? The same is true for Lepidoptera. I think it is absolutely necessary to distinguish between species that at least theoretically can complete their lifecycle within either of these habitats, and those that cannot nest there. For the butterflies and potentially for the wild bees, you could check whether the grasslands (or arable fields) host the plants that the species needs. What wild bee actually breed in any of two habitats?

Response 11: This is an important point. We want to clarify that we did not intend to claim that '[oilseed] rape crops [...] contribute more to landscape-level [wild bee] diversity than grasslands'. We further clarified this in L 241ff with a more nuanced discussion on the interpretation of our results. We observed more wild bee species foraging in oilseed rape fields than in grasslands. While observing foraging wild bees is an indication of their habitat use, it does not mean that the respective habitat is sufficient to complete the life cycle of the observed species. Most of the wild bee species observed foraging in oilseed rape fields will likely not reproduce in these oilseed rape fields (but some might, see e.g. Tschanz et al. 2023). As we did not record the actual nesting sites, we cannot make a statement about the suitability of any of

both habitats for wild bee nesting or reproduction (but see Tschanz et al. 2023 for wild bees reproducing in cereal fields). We agree with the reviewer that detailed life-history trait knowledge would be needed to identify all the habitats necessary for wild bee and butterfly conservation. This information is unfortunately limited for many species present in our dataset and it is unknown whether and how nesting preferences change within the geographic range of a species. In case of butterflies, we can assure that the species detected in large numbers in oilseed rape fields and identified as associated with the habitat (see Response 10 above) can and do reproduce on oilseed rape and other Brassicaceae (e.g. *Pieris rapae* or *Pieris napi*) or common arable weeds (e.g. *Aglais io*, *Aglais urticae, issoria lathonia, Vanessa cardui*; this information was added to the discussion in L 243f). Their larval host plants were commonly found in oilseed rape fields but not in grasslands in our assessment (Supplementary Material I, Table S4), indicating that grasslands do not facilitate their populations and that the habitat types act complementary for biodiversity.

To shed more light on which species are actually favoured by and driving the differences between the two habitat types, we have added a species-by-species analysis of abundance patterns as described above. We included the results of this analysis in Supplementary material I, Tables S4 to S7 alongside a discussion of the main patterns in Supplementary material I. We now also discuss the functional groups and the life-history traits defining the species assemblages that drive differences between the habitat types to the main text including a nuanced discussion of the role of oilseed rape fields for the reproduction of these species (L 221ff).

Comment 12: Third: you could exploit the temporal (several observation times for wild bees and butterflies) and spatial variation (transect) for your data, to understand the species overlap between habitats. Are there species that you find at all times in the same habitat, plus (or not) in a different habitat? Do they only occur close to the margin?

Response 12: Investigating the complementarity of habitats across the season for biodiversity is an excellent next step towards understanding patterns of biodiversity complementarity in agricultural landscapes and needed for efficient conservation.

Plant and insect communities change over the growing season as different species have different phenological niches. Especially in butterflies and wild bees, many species were only active during the first sampling interval (oilseed rape flowering) and filtered out by phenology in subsequent assessments, with a considerable temporal turnover between sampling intervals. Based on the reviewer's comment, and to give a better overview of temporal changes in insect assemblages between the paired habitats, we complemented our previous analysis of the difference in species richness of butterflies, wild bees and carabids between the paired habitats for each of the timesteps with the difference in effective number of species and the overall Bray-Curtis similarity between the paired habitats in each sampling interval (Supplementary Material Fig. S4). In this analysis, we see that neither the difference in species richness nor the difference in effective number of species or assemblage similarity between grasslands and oilseed rape fields changed noticeably across the season, indicating that the value for biodiversity and the complementarity of the habitats remained constant across time. We also investigated the species overlap between the habitats by calculating the proportion of shared species at the level of

individual habitat pairs. We calculated these values only for the insect taxa as plants could not move between paired habitats across the sampling season. In butterflies, most habitat pairs had a considerable overlap in species with the percentage of shared species ranging between 27 and 44 % in grasslands and between 40 and 54 % in oilseed rape fields and remaining fairly constant across sampling intervals (Fig. R2). In wild bees, while 42% and 34% of all species observed in grasslands and oilseed rape fields were shared between the habitats across the whole season, only 4 to 29% and 4 to 30% of the species observed in grasslands and oilseed rape fields in the individual sampling intervals were shared between the paired habitats (Fig. R2). This indicates that some of the species shared across the whole season used the different habitats at different points in time. The largest overlap in species across the whole season was detected in carabids, with 54% and 39% of the species in grasslands and oilseed rape fields shared between the habitats with percentages being rather high also in the individual intervals with 41 to 53% and 27 to 32% in grasslands and oilseed rape fields, respectively (Fig. R2). Here, few habitat pairs shared no species in any of the sampling intervals, and none shared no species across the whole season (Fig. R2). We added this information to the discussion and included Fig. R2 in the supplementary material (L 217; Supplementary Material Fig. S4 & S5). Splitting of our data into separate intervals, however, also reduces the replication available at each point in time. Our sampling was designed to cover the constantly changing plant and insect communities across the season, delivering a good overall coverage of the diversity in the habitats, but not to compare between different points in the phenology. We believe that more replicates / a better temporal resolution would be needed at the individual sampling intervals to be able to investigate temporal patterns meaningfully. While the aspect of temporal changes in species turnover and habitat use is interesting, we find it to be out of the scope of our current study. We are nevertheless happy to expand on this in case the reviewer and editor think that it will make a valuable contribution to our manuscript.

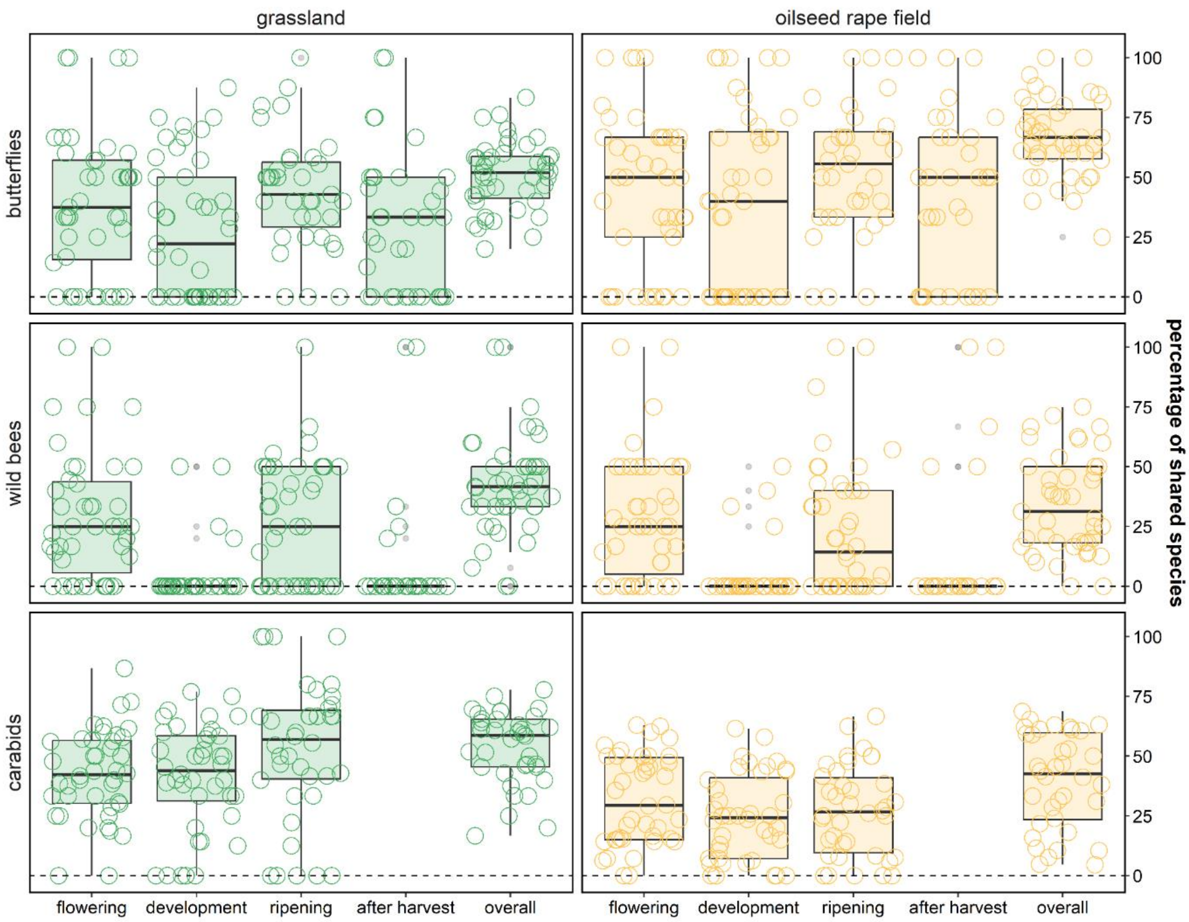

Fig. R2: Percentage of species shared between the paired permanent grasslands (left) and oilseed rape fields (right) of the species assemblages of butterflies (top), wild bees (middle) and carabids (bottom) across the different sampling intervals and across the whole sampling period ('overall').

We were not able to investigate whether species found in one or the other habitat only occurred close to the margins of these habitats as we predominantly sampled at a constant distance to the crop field and grassland margins. Only carabids were collected along a within-habitat distance gradient. While we agree that within habitat distributions of species are interesting and worth investigating, our sampling protocol was not designed with this aspect in mind and thus unfortunately does not have the spatial resolution to investigate within habitat distributions. There is a growing number of studies that address within field distributions of plants (e.g. Yvoz et al. 2021), butterflies and wild bees (e.g. Marja et al., submitted) and carabid beetles (e.g. Boetzl et al. 2024) in crop fields across larger gradients (up to hundreds of meters from the field edges). As we believe that our data is not suited for answering these questions, we did not add anything further here.

Methods:

Comment 13: 326: if I see this correctly, 'country' is 'confounded' (the same) as 'landscape' as there is one landscape only in each country? Otherwise, you would also need to control for 'landscape'.

Response 13: 'Landscape' here refers to the landscape surrounding one habitat or, in our case, a habitat pair, i.e. we assessed a permanent grassland and paired oilseed rape field in each landscape (L 332f). In each country we hence had between eight and ten landscapes (L 333f). We indeed account for this nestedness by including a random intercept in our models which was previously referred to as 'habitat pair'. We now added 'landscape ID' here to make this clearer (L 389).

Results:

Comment 14: 126: what do you mean by 'positively related'? Correlated? How did you test this in a paired design?

Response 14: Now reads 'correlated'. We tested this with simple Pearson correlations correlating the observed species richness in the grassland to the observed species richness in the paired oilseed rape field.

Comment 15: 135: while species richness of wild bees and carabids was higher in oilseed rape fields than in

Response 15: No action taken as the sentence was identical with the version proposed by the reviewer.

Comment 16: 140: your results change if you correct for sample completeness. What does this mean and how did this come about? It has something to do with your sampling effort, and also with the distribution of singletons and doubletons. You do not really discuss this even though you have a good discussion in Supp 2. However, there you point out that the sample completeness leads to 'dubious' results. Your data are real, however, and iNext is based on a theoretical distribution of species which may not reflect the nature of spill-over or other possibilities. So instead of reporting results with and without sample completeness and arguing that they basically show the same thing (they don't) I would use the chance to tackle this issue. Maybe this should be done more extensively in a separate paper, but I think you need to understand yourself what correcting for sample completeness does to your results, and whether the assumption of iNext really apply to your case.

Response 16: While the results do change slightly if we correct for sample completeness as the reviewer states, their interpretation does not. our hypothesis was that permanent grasslands were more diverse than oilseed rape fields which is the case for plants and butterflies but not for wild bees and carabids. Whether wild bee richness is indeed lower or equally high in permanent grasslands than in oilseed rape fields does not affect the rejection of this hypothesis. For transparency, we provide both results and discuss their limitations. We expanded on this discussion and nuanced the interpretation of the lower richness observed in wild bees (L 247ff).

We agree with the reviewer that the use of the iNext (specifically: INext3D) estimation methods is not unproblematic and introduces new pitfalls that require careful interpretation. We have provided these analyses in addition to our analyses based on observed richness for transparency but agree with the reviewer that the nature of our study system, diverse agricultural landscapes where species compositions found in one habitat are not necessarily finite and clearly defined but are affected and enriched by stochastic interactions with their spatial surroundings via

spillover (as the reviewer also stated), likely does not comply with the initial assumptions of the iNext approach. We also agree that due to the continuous spatial nature of our design and the repeated sampling in time that introduces species assemblage changes along the phenology of individual species, the basic assumption of the iNext estimation, i.e. that diversity in a place can be fully assessed, might be violated (as is the case in many other comparable studies). The iNext analyses thus likely overestimate diversity while our field observations surely underestimated (or better: under-recorded) diversity. We agree with the reviewer that a more critical discussion of the iNext approach is warranted and that this would make a worthwhile contribution. But we see ourselves not in the position to include this discussion in this manuscript. We rephrased the wording in the main text (L 149ff, L 213ff) and expanded on the discussion in Supplementary material 2 to better reflect the limitations of this approach. If the reviewer and editor think that it would improve our manuscript, we are ready to drop this alternative analysis and Supplementary Material II altogether.

Comment 17: On the other hand, your sampling effort really differs among countries, for some reason, and your mixture of methods (e.g. pan trap plus observations) makes it difficult to standardize. I would carry out individual analyses for each method and with standardized sampling effort, to see if your patterns hold up to the current 'everthing together' analysis.

Response 17: We want to point out that the sampling effort and methods did overall not differ between countries. Only butterflies in Bulgaria and wild bees in Bulgaria and Sweden were collected less often than in the other countries due to manpower and financial constraints. The sampling effort within each country was standardised (except for carabid beetles where unpredictable trap losses due to wild animals and management made additional standardisation on the habitat pair level necessary which we believe is rather an advantage of our study as it is rarely done in comparable studies). This heterogeneity is covered by the inclusion of 'country' in our models and its interactions with 'grassland amount'. Further the reviewer refers to a 'mixture of methods' which can only refer to wild bees where we combined transect walk and pan trap samples - all other taxa were solely sampled with one method. All sampling methods come with individual biases and different sampling methods are often complementary. For wild bees, the complementarity of pan traps and sweep netting has been demonstrated previously and various authors have proposed to combine these methods in biodiversity assessments of wild bees which we followed here (see e.g. Westphal et al. 2008, Pendergast et al. 2020).

Based on the reviewer's remarks, we analysed the wild bee data separately for pan trap and sweep net samples and summarised the results and differences in Table R1 below. While the majority of effects and all strong effects remain consistent between the combined sample and the separate pan trap and sweep net samples, there are some minor differences. The pan trap samples collect a higher effective number of species in oilseed rape fields and find both species richness as well as effective number of species to be positively related, albeit weakly, to grassland amount at the 1000 m scale. The sweep net sample shows the effective number of species weakly negatively related to grassland amount at the 2000 m scale. In the assemblage-based analyses (PERMANOVA & beta diversity), many effects are lost and the overall effects are more influenced by the pan trap sample than by the sweep net samples which could be expected as these analyses are abundance-based and 77.5 % of the individuals were collected using pan traps (Supplementary Material I). We conclude that combining the methods delivers

a more complete picture of how wild bee assemblages are shaped by habitat type and landscape-level grassland amount. Of the 195 wild bee species collected in total, 137 were found in pan traps and 132 in the sweep net transects. This shows that many species present in the habitats were not covered when only one of the methods was used, which likely biases predictions for their total diversity more than a combination of different methods. We thus decided to maintain our combined sample in the analyses. Nevertheless, while not the focus of our study, the observed differences between methods may be of methodological interest and could be explored more extensively in a dedicated paper.

Table R1: Additional analyses performed for bee pan trap and sweep net samples in comparison to the combined analyses presented in the main text. Significant effects are highlighted according to their strength ((*) indicates $p < 0.10$, * indicates $p < 0.05$, ** indicates $p < 0.01$, *** indicates $p < 0.001$), for continuous predictors '[+]' indicates a positive relationship, '[-]' indicates a negative relationship. Effects that differ from the combined models presented in the main text are highlighted in light red.

	combined		pan trap samples		sweep net samples	
	1000 m	2000 m	1000 m	1000 m	2000 m	1000 m
**species richness**						
habitat type	***	***	***	***	***	***
grassland amount			* [+]			
country	*	(*)	**	(*)	***	***
grassland amount × habitat type						
grassland amount × country			*	*		
habitat type × country	*	*	**	*		
**effective number of species**						
habitat type			**	**		
grassland amount			* [+]			* [-]
country	***	***	***	**	***	***
grassland amount × habitat type						
grassland amount × country						
habitat type × country						
**assemblages (PERMANOVA)**						
grassland amount	**		**	(*)		
habitat type	(*)	*	*	*		
country					(*)	
distance between paired habitats						
grassland amount × habitat type					(*)	
grassland amount × country						
habitat type × country			(*)	(*)		
**beta diversity (overall)**						
grassland amount	(*)	(*)	(*)	*		
country			*	(*)		
distance between paired habitats						
grassland amount × country		*			**	**
**beta diversity (turnover)**						
grassland amount	(*)	*	(*)	(*)		
country			*	*		
distance between paired habitats						
grassland amount × country	*	*		(*)	**	*
**beta diversity (nestedness)**						
grassland amount		*				
country			(*)	**		
distance between paired habitats						
grassland amount × country		*				

References

Boetzl, F. A., D. Sponsler, M. Albrecht, P. Batáry, K. Birkhofer, M. Knapp, J. Krauss, B. Maas, E. A. Martin, C. Sirami, L. Sutter, C. Bertrand, A. B. Baillod, G. Bota, V. Bretagnolle, L. Brotons, T. Frank, M. Fusser, D. Giralt, E. González, A. R. Hof, H. Luka, R. Marrec, M. A. Nash, K. Ng, M. Plantegenest, B. Poulin, G. M. Siriwardena, T. Tscharntke, M. Tschumi, A. Vialatte, L. Van Vooren, M. Zubair-Anjum, M. H. Entling, I. Steffan-Dewenter, and J. Schirmel. 2024. Distance functions of carabids in crop fields depend on functional traits, crop type and adjacent habitat: a synthesis. *Proceedings of the Royal Society B*: Biological Sciences 291: 20232383.

Fahrig, L. 2013. Rethinking patch size and isolation effects: the habitat amount hypothesis. *Journal of Biogeography* 40: 1649-1663.

Gesellschaft für Angewandte Carabidologie. 2009. Lebensraumpräferenzen der Laufkäfer Deutschlands -Wissensbasierter Katalog. Page 48. Angewandte Carabidologie Supplement.

Prendergast, K. S., M. H. M. Menz, K. W. Dixon, and P. W. Bateman. 2020. The relative performance of sampling methods for native bees: an empirical test and review of the literature. *Ecosphere* 11: e03076. 10.1002/ecs2.3076

Tscharntke, T., J. M. Tylianakis, T. A. Rand, R. K. Didham, L. Fahrig, P. Batáry, J. Bengtsson, Y. Clough, T. O. Crist, C. F. Dormann, R. M. Ewers, J. Fründ, R. D. Holt, A. Holzschuh, A. M. Klein, D. Kleijn, C. Kremen, D. A. Landis, W. Laurance, D. Lindenmayer, C. Scherber, N. Sodhi, I. Steffan-Dewenter, C. Thies, W. H. van der Putten, and C. Westphal. 2012. Landscape moderation of biodiversity patterns and processes - eight hypotheses. *Biological Reviews* 87: 661-685.

Westphal, C., R. Bommarco, G. Carré, E. Lamborn, N. Morison, T. Petanidou, S. G. Potts, S. P. M. Roberts, H. Szentgyörgyi, T. Tscheulin, B. E. Vaissière, M. Woyciechowski, J. C. Biesmeijer, W. E. Kunin, J. Settele, and I. Steffan-Dewenter. 2008. Measuring bee diversity in different European habitats and biogeographical regions. *Ecological Monographs* 78: 653-671.

Yvoz, S., S. Petit, E. Cadet, F. Dessaint, and S. Cordeau. 2021. Taxonomic and functional characteristics of field edge weed communities along a gradient of crop management

intensity. *Basic and Applied Ecology* 57: 14-27.

Reviewer #1 (Remarks to the Author):

In their first version, the authors already provided a manuscript with high quality study design, methods and analyses. My main suggestions for improvement were related to the structure and focus of the Introduction and results section. The authors have taken those suggestions to heart and greatly improved the clarity, focus and main messages of the manuscript:

The motivation and ecological theory behind the hypotheses are now much clearer. It's also now much easier to understand why it is interesting to look at both a crop/non-crop comparison and at the effect of non-crop habitat amount in the Introduction.

Now that the results are clearly linked to the three hypotheses and focus on the main indicators and scales they are much easier to follow. With all the analyses being shown in the Supplementary Materials, the message is still both convincing and nuanced enough.

Overall, I have only minor comments which can easily be addressed. Therefore, I suggest to accept the manuscript.

Response: We are very grateful for the input the reviewer provided that helped strengthen the hypothesis-driven focus and the message of our manuscript. We thank the reviewer for their kind comments and support!

Line 127: "declining" should be "declines" or "reduces"

Response: Corrected as suggested (‘reduces’).

line 232: one "neither" can be removed

Response: Corrected as suggested (first ’neither’ removed)

line 241 to 245: try splitting the sentences in parts as it is very long right now

Response: We split the sentences to enhance the text flow.

line 254: remove "a" before "twelve"

Response: Corrected as suggested.

Reviewer #2 (Remarks to the Author):

This revised study now more extensively analyses differences between oilseed rape fields and permanent grasslands of diversity and community composition of four different taxonomic groups in pairs of sampled across a large number of landscapes and countries. The authors show that species richness and diversity is not generally higher in permanent grasslands compared to crop fields and that crop fields support a species assemblage that is complementary to the ones in permanent grasslands. In addition, the manuscript now contains more information at the species level that helps to put the results into perspective. This manuscript is vastly improved and should eventually be published in Nature Communications, but I think it can be made even stronger with some more revision. In particular, some of the analyses shown in the cover letter should be integrated into the paper/OSM. In addition, I do suggest some extra work. The points raised in my last review that were addressed satisfactorily (most points) will not not be mentioned below.

Response: We are very grateful for the constructive input and have addressed the suggestions in detail below. In particular, we added an updated and polished version of the requested previous analyses, added information and analyses on grassland management, brought into the manuscript a re-worked version of a figure previously only shown in the response letter and restructured the Supplementary Material as suggested by the reviewer.

One information that is missing from your paper is the intensity with which your grasslands were managed – there is a huge difference between grasslands in diversity depending on whether they are grazed or mown. While you state that your grasslands were not fertilized (which is surprising), they could still be cut 1-3 times per year, or grazed at different intensities. My prediction would be that the difference between crop field and grassland increases with decreasing land use intensity in the grasslands, which you can test.

Response: As stated before, we unfortunately lack the detailed management information for both habitats. We have reconstructed coarse management information for the grasslands either derived from farmer questionnaires or from our own observations of the sites during fieldwork and in preceding and subsequent years. Of the 43 grasslands, 30 were grazed, 20 thereof extensively (< 1 livestock units) and 10 at moderate intensity (< 5 livestock units). Grazing events were predominantly limited in time and often performed by vagrant shepherds. 16 grasslands were mown, all but two only once per year in the end of July or early August (we have no information whether the two grasslands where farmers stated that they were mown 'up to two times per year' were actually mown twice in the respective year). Four grasslands were

mown and / or grazed depending on the respective year, with two of them being mown in the end of July and used for grazing by shepherds afterwards in autumn and the remaining two being either mown once or twice per year or grazed at low intensity (< 1 livestock units). Due to a lack of detailed management information, we can unfortunately not say which management and at which intensity was actually taking place in the respective year. We thus classified grassland management coarsely into five categories: unmanaged, mown, mown & extensively grazed, extensively grazed, moderately grazed. These categories were not equally distributed across regions and sample sizes for some categories (unmanaged, mown and / or extensively grazed) were very low which in combination made it impossible to reasonably include this factor into the models (Fig. R2-1). To assess whether grassland management may have shaped diversity, we plotted species richness, effective number of species and the beta diversity between both paired habitats in relation to grassland management and provide this as a supplementary figure (Supplementary Fig. S2-14). Although the low sample size made it impossible to test differences statistically, the plots indicate that management had little to no effect on the diversity measures in the grasslands with ‘country’ clearly shaping diversity more than grassland management.

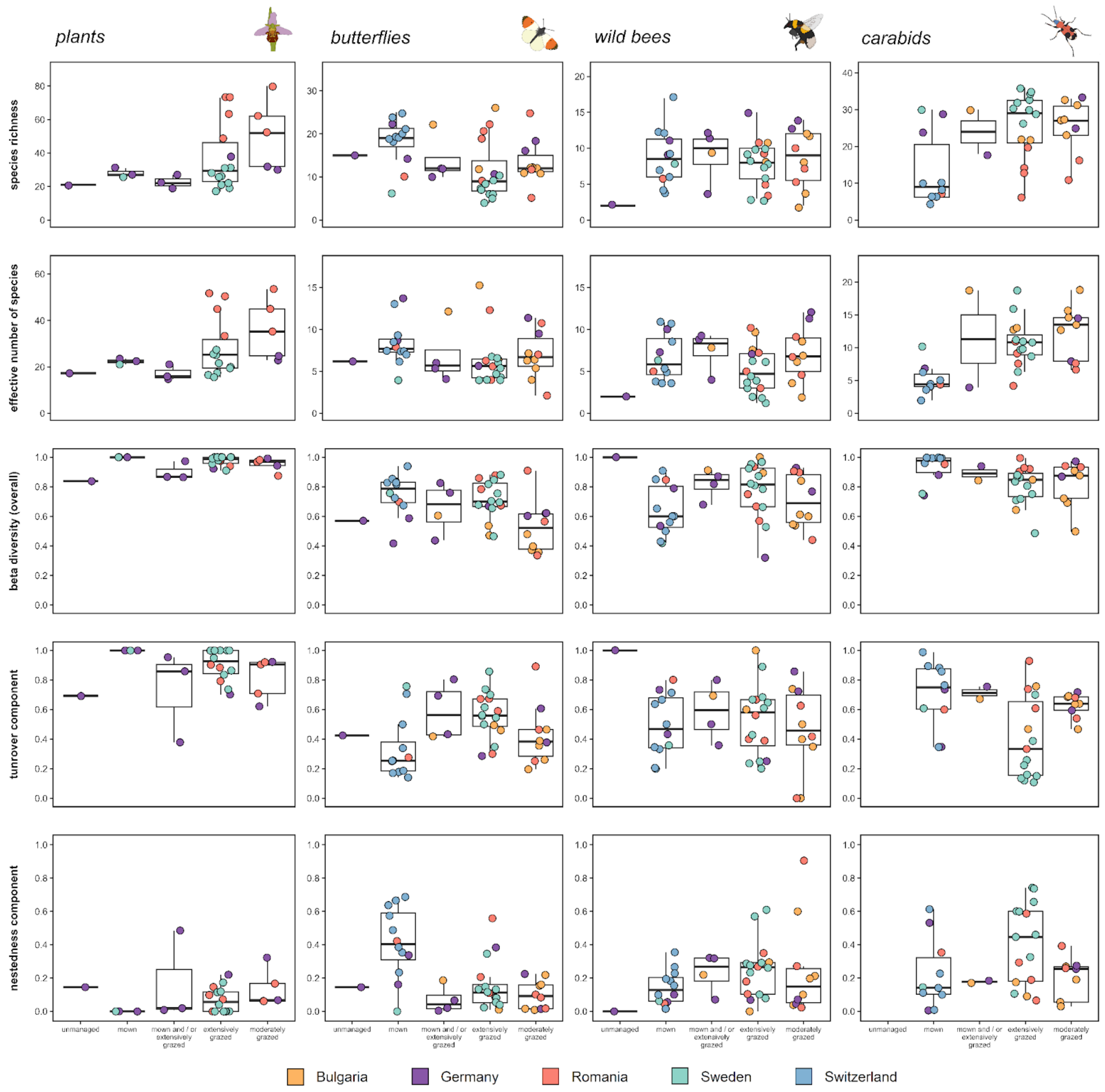

Fig. R2-1: Species richness and effective number of species of plants, butterflies, wild bees and carabids recorded in grasslands as well as their overall beta diversity and its turnover and nestedness components between grasslands and paired oilseed rape fields in relation to grassland management. Management categories are coarse estimations as no detailed management information was available. This figure is also included in Supplementary Fig. S2-14.

We believe that this coarse classification and the lack of detailed management information does unfortunately not allow the calculation of a land-use intensity gradient as proposed by the reviewer. We can thus unfortunately not analyse whether a decline in grassland management intensity indeed increased the complementarity of the habitats. We highlighted this now more clearly as a possible limitation in the discussion (L 317ff). However, while management intensity of the grasslands likely varied as suspected by the reviewer, we want to point out that the same is likely the case for oilseed rape fields. Here farmers could have used inversion tillage

or not and could have used a varying number of insecticide applications or not, with concrete management decisions being made based on local conditions and the presence and abundance of crop pests in the respective year and decisions being influenced by the experience of the respective farmers. Grassland management intensity alone therefore likely does not define the degree of complementarity between the paired habitats.

In addition, we want to point out that in Sweden, where all grasslands were managed at low intensity with extensive grazing (apart from one site that was mown once per year) and in Switzerland where all sites were mown at low intensity once per year in late July / early August, patterns were overall not noticeably different from the other countries where grassland management intensities were likely more mixed. In our opinion, the high plant species richness encountered in grasslands across countries indicates that all grasslands had a semi-natural character and considerable value for biodiversity.

Re-reading the paper I thought it would be useful if you could refer to gamma-diversity of your landscapes (as you discuss alpha and beta), and could also analyse how much beta contributes to gamma.

Response: We thank the reviewer for this suggestion. Because our design includes exactly two paired habitats per landscape, landscape-level gamma diversity is conceptually determined by the alpha diversity of each habitat and the beta diversity between them. As we already quantify alpha diversity in both habitats and show consistently high between-habitat beta diversity that is predominantly driven by species turnover rather than nestedness, our current results already imply that habitat complementarity makes a major contribution to landscape-level gamma diversity. We therefore consider an explicit alpha–beta–gamma partitioning largely redundant and unlikely to provide additional ecological insight beyond our existing analyses of alpha and beta diversity. We added a passage highlighting this to the discussion (L 284ff).

Additional analysis

I appreciate the extra text provided with explanations and detailed discussion of individual species. However, you do not really refer to this in your paper, and you omit some of the explanations and extra figures you provide within the cover letter, e.g. Figure R1 (only in the letter).

I suggest to re-structure the supplementary material into logical sections, instead of only labelling each figure and table consecutively. This allows you to group tables and figures together and allows you to add extra text.

For example, “Supplementary Material 1” could be the extended methods, with figures S1-1, S1-2 (instead of Fig. S1, S2)

You could then put ‘Detailed discussion: Species driving habitat differences across countries’ as a separate ‘Supplementary material x’. This more detailed discussion could go along with Fig. S5. At present, your extra text gets lost.

Response: We restructured the supplementary material based on the suggestions provided by reviewer 2 into 4 chapters:

Chapter 1 - Extended methodology

Chapter 2 - Extended results with additional figures and tables

Chapter 3 - Detailed discussion on species driving habitat differences across countries

Chapter 4 - Alternative, sample coverage-standardised analyses with discussion

We renamed all Figures and Tables accordingly, as suggested by the reviewer. The Supplementary Information is still provided in a single, combined file following the journal submission guidelines (‘Please submit the Supplementary Information as a single Word file, if possible, otherwise a single PDF.’).

Figure R1 from the previous response letter was intended as information for the reviewer and is not very illustrative in the form presented there. We re-worked this figure highlighting species that are only found in one of the respective countries and highlighting species that were identified as habitat-associated in at least one of the countries and thus likely drive assemblage differences between the habitats. The figure is found in (the new) Supplementary Fig. S3-3 and displayed below as Fig. R2-2 for convenience.

[REDACTED]

Fig. R2-2: Species assemblages of plants (A), butterflies (B), wild bees (C) and carabids (D; NMDS ordinations) in the two sampled habitat types (oilseed rape and grassland) across five European countries (Bulgaria, Germany, Romania, Sweden and Switzerland). Points indicate the individual species in the assemblages; numbers refer to the rows in the species lists provided in Supplementary Tables S3-1 to S3-4. Coloured cores indicate that the respective species was exclusively recorded in the respective country and likely to drive assemblage differences between countries, coloured outer rings indicate that the respective species was determined as habitat-associated species that is likely to drive assemblage differences between habitats (Supplementary Tables S3-1 to S3-4). The NMDS used Bray-Curtis distances on proportional abundances or proportions of cover (plants). For statistics, see text and Supplementary Table S2-4. This figure is also included in Supplementary Table S3-3.

Trait analysis

I am not really convinced by your argument that traits vary too much between countries and within species, so that an analysis is not possible. There are many global analyses that succeed doing such analysis. I would find it extremely interesting if you could say something about the

differences between the species that prefer the different habitats, or occur in both. You hint at this when you say that some carabids are 'agrophilous', yet this is just a description of where they have been observed, mainly. For example, plants in rape fields are probably more annuals rather than perennials. More interesting is however, what traits allow the species to persist in the temporary (crop) vs. permanent fields – what butterfly traits make it possible that they can survive in the habitats? Phenology of the larvae? Overwintering strategy? I do not insist on such an analysis, you could do this in a separate paper, but it would make the current paper even stronger.

Response: We agree that exploring traits that shape species assemblages in response to varying management or habitat continuity is a very interesting topic and thank the reviewer for their input! Detailed analyses, however, go beyond the scope of this manuscript. For many of the species covered here, traits are not readily available in repositories, especially for those exclusively found on the Balkan peninsula. Large-scale syntheses usually solve this problem by simply neglecting species with missing trait information which is not a feasible strategy for identifying traits or trait-syndromes that shape assemblages as trait availability is likely strongly biased by the distribution and the degree of ubiquitism of a species. In response to the reviewer's request, we provided some additional information on selected traits of the species found associated with one of the two habitat types in the new Supplementary Fig. S3-4; displayed for convenience as Fig. R2-3 below). Note that, while plant family is not per se a trait, many functionally important traits are determined at the family level in plants. Trait availability for this selected subset of species was high, albeit some species are already missing information here (especially in carabids). Following the advice of the reviewer, we intend to revisit this question in more detail in a future manuscript.

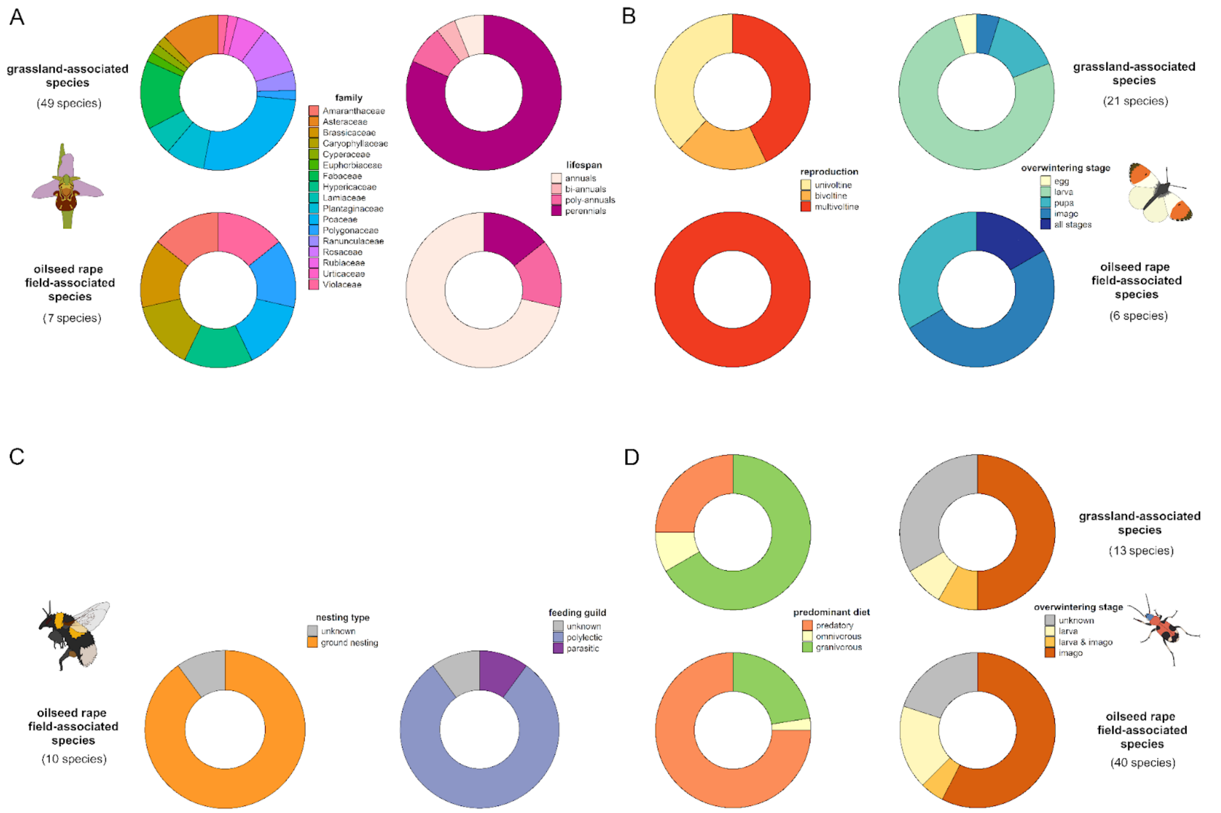

Fig. R2-3: Distribution of selected traits among species identified as associated with one of the two habitat types. Donut charts indicate the proportional representation of the respective traits among the habitat-associated species in both habitat types. Traits include family and lifespan in plants (A; taken from Kleyer et al. 2008), reproduction strategy and overwintering stage in butterflies (B; taken from Middleton-Welling et al. 2020), nesting type and feeding guild in wild bees (C; taken from Westrich 2019) and predominant diet and overwintering stage in carabids (D; taken from Lindroth 1992 and Boetzl et al. 2024).

Temporal patterns

Again, very interesting (Fig R2) and should be added to paper/supplement. In fact, you could also calculate, for each species, a relative specialisation measure, as the number of times it was observed in one habitat vs. total number of observations. This would go beyond your current analysis. Analysing the temporal variation, and also the strength of specialisation in a little more detail would help us to understand better why those habitats complement each other to increase gamma diversity at the landscape scale.

Response: Figure R2 from the previous response letter had already been included as Figure S5 in (the previous) Supplementary Information I (now Supplementary Fig. S3-2) following the previous round of revisions. From this Figure, we see that the percentage of shared species in the butterfly, wild bee and carabid assemblages of both grasslands and oilseed rape fields

remained fairly stable across time (and consequently, so did the shares of unique species recorded in both habitats). Based on this, we see little potential for additional analyses of potential temporal changes. We sampled repeatedly across the growing season to obtain a good snapshot of the overall biodiversity in the habitats across plant and insect phenology, but the study was not designed to obtain data for analysing species assemblages along the phenology. We believe that much higher sampling frequency would have been needed for a detailed analysis of potential temporal changes.

In addition, the reviewer suggests to calculate habitat specialisations at the species level as the proportion of abundances of the respective species in a habitat type. While this might seem as an attractive option, we see this as problematic: All species recorded as singletons would be returned as absolute habitat specialists as their proportion would necessarily be 100% in the respective habitat. Similarly, a species occurring in all grasslands of a country at low abundances but in only one of the oilseed rape fields at a high abundance could be returned as oilseed rape-specialised although this is likely not true and caused by spillover from an adjacent habitat. We believe that our statistical analyses of species-habitat associations added in response to the previous comments by reviewer 2 in the previous round of revisions and discussed in (the new) Supplementary Tables S3-1 to S3-4 and Figs S3-3 and S3-4 already provides the information requested here. This analysis is done at the species level and based on abundance distributions but less sensitive to singletons and species that are by chance abundant in single sites as they are based on averages and, more importantly, their respective standard errors. We deem these analyses thus more trustworthy than the analyses proposed above. We hence believe that we have already delivered what the reviewer is asking for. We now further illustrate these results in the NMDS ordination in Supplementary Fig. S3-3.

References

Boetzl, F. A., D. Sponsler, M. Albrecht, P. Batáry, K. Birkhofer, M. Knapp, J. Krauss, B. Maas, E. A. Martin, C. Sirami, L. Sutter, C. Bertrand, A. B. Baillod, G. Bota, V. Bretagnolle, L. Brotons, T. Frank, M. Fusser, D. Giralt, E. González, A. R. Hof, H. Luka, R. Marrec, M. A. Nash, K. Ng, M. Plantegenest, B. Poulin, G. M. Siriwardena, T. Tscharntke, M. Tschumi, A. Vialatte, L. Van Vooren, M. Zubair-Anjum, M. H. Entling, I. Steffan-Dewenter, and J. Schirmel. 2024. Distance functions of carabids in crop fields depend on functional traits, crop type and adjacent habitat: a synthesis. Proceedings of the Royal Society B: Biological Sciences **291**:20232383.

Kleyer, M., R. M. Bekker, I. C. Knevel, J. P. Bakker, K. Thompson, M. Sonnenschein, P. Poschlod, J. M. Van Groenendael, L. Klimeš, J. Klimešová, S. Klotz, G. M. Rusch, M. Hermy, D. Adriaens, G. Boedeltje, B. Bossuyt, A. Dannemann, P. Endels, L. Götzenberger, J. G. Hodgson, A.-K. Jackel, I. Kühn, D. Kunzmann, W. A. Ozinga, C. Römermann, M. Stadler, J. Schlegelmilch, H. J. Steendam, O. Tackenberg, B. Wilmann, J. H. C. Cornelissen, O. Eriksson, E. Garnier, and B. Peco. 2008. The LEDA Traitbase: a database of life-history traits of the Northwest European flora. Journal of Ecology **96**:1266-1274.

Lindroth, C. H. 1992. Ground beetles (Carabidae) of Fennoscandia. A zoogeographic study. Part 1: Specific knowledge regarding the species. (Translation of: Die fennoskandischen Carabidae.). Smithsonian Institution Libraries and The National Science Foundation, Washington, D.C.

Middleton-Welling, J., L. Dapporto, E. García-Barros, M. Wiemers, P. Nowicki, E. Plazio, S. Bonelli, M. Zaccagno, M. Šašić, J. Liparova, O. Schweiger, A. Harpke, M. Musche, J. Settele, R. Schmucki, and T. Shreeve. 2020. A new comprehensive trait database of European and Maghreb butterflies, Papilionoidea. Scientific Data **7**:351.

Westrich, P. 2019. Die Wildbienen Deutschlands. Verlag Eugen Ulmer, Stuttgart.
